# Hierarchical optimization for the efficient parametrization of ODE models

**DOI:** 10.1093/bioinformatics/bty514

**Published:** 2018-07-13

**Authors:** Carolin Loos, Sabrina Krause, Jan Hasenauer

**Affiliations:** 1 Helmholtz Zentrum München—German Research Center for Environmental Health, Institute of Computational Biology, Neuherberg, Germany; 2 Chair of Mathematical Modeling of Biological Systems, Center for Mathematics, Technische Universität München, Garching, Germany

## Abstract

**Motivation:**

Mathematical models are nowadays important tools for analyzing dynamics of cellular processes. The unknown model parameters are usually estimated from experimental data. These data often only provide information about the relative changes between conditions, hence, the observables contain scaling parameters. The unknown scaling parameters and corresponding noise parameters have to be inferred along with the dynamic parameters. The nuisance parameters often increase the dimensionality of the estimation problem substantially and cause convergence problems.

**Results:**

In this manuscript, we propose a hierarchical optimization approach for estimating the parameters for ordinary differential equation (ODE) models from relative data. Our approach restructures the optimization problem into an inner and outer subproblem. These subproblems possess lower dimensions than the original optimization problem, and the inner problem can be solved analytically. We evaluated accuracy, robustness and computational efficiency of the hierarchical approach by studying three signaling pathways. The proposed approach achieved better convergence than the standard approach and required a lower computation time. As the hierarchical optimization approach is widely applicable, it provides a powerful alternative to established approaches.

**Availability and implementation:**

The code is included in the MATLAB toolbox PESTO which is available at http://github.com/ICB-DCM/PESTO

**Supplementary information:**

Supplementary data are available at *Bioinformatics* online.

## 1 Introduction

Mechanistic mathematical models are used in systems biology to improve the understanding of biological processes. The mathematical models most frequently used in systems biology are probably ordinary differential equations (ODEs). ODE models are, among others, used to describe the dynamics of biochemical reaction networks (Kitano, 2002; Klipp *et al.*, 2005; Schoeberl *et al.*, 2009) and proliferation/differentiation processes (De Boer *et al.*, 2006). The parameters of the underlying processes, e.g. rate constants and initial conditions, are often unknown and need to be inferred from available experimental data. The inference provides information about the plausibility of the model topology, and the inferred parameters might for instance be used to predict latent variables or the response of the process to perturbations (Molinelli *et al.*, 2013).

The experimental data used for parameter estimation are produced by various experimental techniques. Most of these techniques provide relative data, meaning that the observation is proportional to a variable of interest, e.g. the concentration of a chemical species. This is for instance the case for Western blotting (Renart *et al.*, 1979) and flow and mass cytometry (Herzenberg *et al.*, 2006). If calibration curves are generated, the measured intensities can be converted to concentrations, however, in most studies this is not done due to increased resource demands.

In the literature, two methods are employed to link relative data to mathematical models: (i) evaluation of relative changes (Degasperi *et al.*, 2017) and (ii) introduction of scaling parameters (Raue *et al.*, 2013). In (i), relative changes between conditions are compared, and the differences between observed and simulated relative changes are minimized. While this approach is intuitive and does not alter the dimension of the fitting problem, the noise distribution is non-trivial and the residuals are not uncorrelated (Thomaseth and Radde, 2016), which is often disregarded (see, e.g. Degasperi *et al.*, 2017). This can in principle result in incorrect confidence intervals (see Supplementary Section S6). In (ii), scaling parameters are introduced to replace the calibration curves. The scaling parameters are unknown and have to be inferred along with the remaining parameters of the model, which we refer to as dynamic parameters throughout this manuscript (although they do not change over time). While this increases the dimensionality of the optimization problem [see (Bachmann *et al.*, 2011) for an example in which the number of parameters is doubled], the noise distribution is simple and the confidence intervals consistent. To address the dimensionality increase, Weber *et al.* (2011) proposed an approach for estimating the conditionally optimal scaling parameters given the dynamic parameters. This approach eliminated the scaling parameters, however, it is only applicable in the special case of additive Gaussian noise with known standard deviation. Estimating the noise parameters instead of providing the standard deviations has been shown to yield a statistically more accurate assessment of the model (Raue *et al.*, 2013). Unknown noise parameters and outlier-corrupted data (Maier *et al.*, 2017)—as found in many applications—cannot be handled by the approach of Weber *et al.* (2011).

In this study, we propose a hierachical optimization approach which generalizes the idea of Weber *et al.* (2011). The proposed hierarchical approach allows for arbitrary noise distributions, with known and unknown noise parameters. In this manuscript, we focus on Gaussian noise, which is most commonly used, and Laplace noise, which has shown to be beneficial in the presence of outliers (Maier *et al.*, 2017). For the two noise distributions, Gaussian and Laplace noise, we provide analytic solutions for the inner optimization problem, which boosts the computational efficiency. To illustrate the properties of the proposed approach, we present results for two models of JAK-STAT signaling and a model of RAF/MEK/ERK signaling.

## 2 Materials and methods

In this section, we describe the considered class of parameter estimation problems and introduce a hierarchical optimization method for estimating the parameters of ODE models from relative data under different measurement noise assumptions.

### 2.1 Mechanistic modeling of biological systems

We considered ODE models of biological processes,

(1) $$\dot{x}=f(x(t,\theta ),\theta ),\;x(t_{0},\theta )=x_{0}(\theta ),$$

in which the time- and parameter-dependent state vector $x(t,\theta )\in {\mathbb{R}}^{n_{x}}$ represents the concentrations of the species involved in the process and the vector field $f:{\mathbb{R}}^{n_{x}}\times {\mathbb{R}}^{n_{\theta }}\to {\mathbb{R}}^{n_{x}}$ determines how the concentrations evolve over time. The vector $\theta \in {\mathbb{R}}^{n_{\theta }}$ denotes the parameters of the system, e.g. rate constants. The initial conditions at time point *t*_0_ are given by the parameter-dependent function $x_{0}:{\mathbb{R}}^{n_{\theta }}\to {\mathbb{R}}^{n_{x}}$.

Experimental data provide information about observables $y(t,\theta )\in {\mathbb{R}}^{n_{y}}$. These are obtained by the observation function $h:{\mathbb{R}}^{n_{x}}\times {\mathbb{R}}^{n_{\theta }}\to {\mathbb{R}}^{n_{y}}$, which maps the states and parameters to the observables via

(2) y(t,θ)=h(x(t,θ),θ).

Due to experimental limitations the experimental data is noise corrupted,

(3) $${\bar{y}}_{i,k}=h_{i}(x(t_{k},\theta ),\theta )+\varepsilon_{i,k},$$

with *h_i_* denoting the *i*th component of the observation function **h**, and indices *k* for the time point. In most applications, Gaussian noise is assumed, $\varepsilon_{i,k}\;\;˜\;\;N(0,\sigma_{i,k}{}^{2})$. For outlier-corrupted data, it was shown that the assumption of Laplace noise, $\varepsilon_{i,k}∼\text{Laplace}(0,\sigma_{i,k})$, yields more robust results (see (Maier *et al.*, 2017) and references therein).

The measurements are collected in a dataset $D={\left\{{\bar{y}}_{k},t_{k}\right\}}_{k}$. The vector ${\bar{y}}_{k}={\left({\bar{y}}_{1,k},\ldots ,{\bar{y}}_{n_{y},k}\right)}^{T}$ comprises the measurements for the different observables. For the general case including different experiments and conditions, we refer to the Supplementary Section S1.

### 2.2 Relative experimental data

Many experimental techniques provide data which are proportional to the measured concentrations. The scaling parameters are usually incorporated in **h**, defined in (2). Here, for simplicity and without loss of generality, we factored-out the scaling parameters from the function **h** and write

$${\bar{y}}_{i,k}=s_{i,k}\cdot \;h_{i}(x(t_{k},\theta ),\theta )+\varepsilon_{i,k}.$$

The scaling parameters *s_i_*_,__*k*_ and the noise parameters *σ_i_*_,__*k*_ are in the following combined in the matrices **s** and σ, respectively. To distinguish the different parameter types, we refer to the parameters θ further as dynamic parameters. In the following, we present results for the case that the scaling *s_i_* and noise parameters *σ_i_* are the same for each time point, but differ between observables. The general case is presented in the Supplementary Section S1.

### 2.3 Formulation of parameter estimation problem from relative data

We used maximum likelihood methods, a commonly used approach to calibrate mathematical model, to estimate the parameters from experimental data. The likelihood function is given by

(4) $$L(\theta ,s,\sigma )=\prod_{i,k}p({\bar{y}}_{i,k}|s_{i}\cdot h_{i}(x(t_{k},\theta ),\theta ),\sigma_{i})$$

with *p* denoting the conditional probability of ${\bar{y}}_{i,k}$ given the observable $y_{i,k}=s_{i}\cdot h_{i}(x(t_{k},\theta ),\theta )$. This probability is for Gaussian noise

$$p({\bar{y}}_{i,k}|y_{i,k},\sigma_{i})=\frac{1}{\sqrt{2\pi }\sigma_{i}}\text{exp}\;\left(-\frac{{\left({\bar{y}}_{i,k}-y_{i,k}\right)}^{2}}{2\sigma_{i}^{2}}\right)$$

with standard deviation *σ_i_* > 0, and for Laplace noise

$$p({\bar{y}}_{i,k}|y_{i,k},\sigma_{i})=\frac{1}{2\sigma_{i}}\text{exp}\;\left(-\frac{\left|{\bar{y}}_{i,k}-y_{i,k}\right|}{\sigma_{i}}\right).$$

with scale parameter *σ_i_* > 0.

#### 2.3.1 Standard approach to parameter estimation

For the standard approach, the dynamic parameters θ, the scaling parameters **s**, and the noise parameters σ are estimated simultaneously. For numerical reasons, this is mostly done by minimizing the negative log-likelihood function,

(5) $$\underset{\theta ,s,\sigma }{\min}J(\theta ,s,\sigma )\;\text{with}\;J(\theta ,s,\sigma )=-\text{log}\;L(\theta ,s,\sigma )\;.$$

**Fig. 1.**
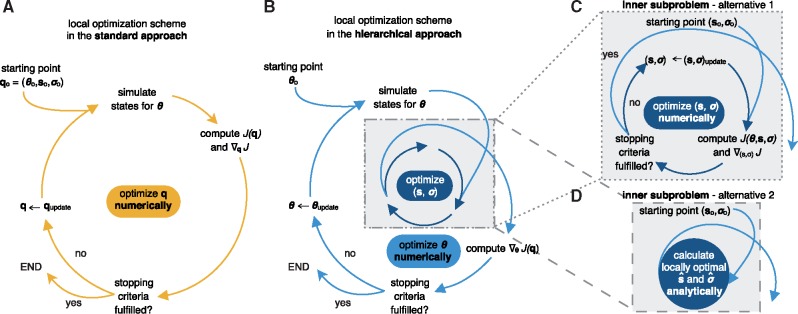
Visualization of standard and hierarchical optimization schemes. (**A**) Local optimization in the standard approach with parameters q=(θ,s,σ). A single iteration includes the numerical simulation of the ODE model for θ, the evaluation of the objective function and its gradient, the evaluation of stopping criteria, e.g. local optimality, and the termination of the local optimization or the updating of the parameters. (**B**) Outer local optimization in the hierarchical approach with parameters θ. A single iteration includes the numerical simulation of the ODE model for θ. the evaluation of the objective function and its gradient with respect to θ using the results of the inner optimization problem. The iteration also includes the evaluation of stopping criteria, and the termination of the local optimization or the updating of parameters. (**C, D**) Inner (local) optimization in the hierarchical approach to find the optimal scaling and noise parameter $\hat{s}$ and $\hat{\sigma }$ for given dynamic parameters θ. (**C**) Iterative local optimization to determine $\hat{s}$ and $\hat{\sigma }$. This does not require the numerical simulation of the model. (**D**) Calculating optimal parameters $\hat{s}$ and $\hat{\sigma }$ using analytic expressions for common noise distributions

The parameters were combined as q=(θ,s,σ) and the optimization problem has the dimension: number of dynamic parameters *n_θ_* + number of scaling parameters *n_s_* + number of noise parameters *n_σ_*. We solved the optimization problem using multi-start local optimization (see, e.g. Raue *et al.* 2009). In each iteration the objective function and its gradient were computed. If the objective function for this parameters fulfills certain criteria, e.g. the norm of the gradient was below a certain threshold, the optimization was stopped, otherwise the parameter was updated and the procedure was continued (Fig. 1A).

#### 2.3.2 Hierarchical approach to parameter estimation

Since the optimization problem (5) often possess a large number of optimization variables and can be difficult to solve, we exploited its structure. Instead of solving simultaneously for θ,s, and σ, we considered the hierarchical optimization problem (Fig. 1B–D)

(6) $$\underset{\theta }{\min}\;J(\theta ,\hat{s}(\theta ),\hat{\text{σ}}(\theta ))$$

(7) $$\text{with }(\hat{s}(\text{θ}),\hat{\sigma }(\text{θ}))=\underset{s,\text{σ}}{\mathrm{argmin}}\;J(\text{θ},s,\sigma ).$$

The inner problem (7) provides the optimal values $\hat{s}(\theta )$ and $\hat{\sigma }(\theta )$ of **s** and σ given θ. These optimal values were used in the outer subproblem to determine the optimal value for θ denoted by $\hat{\theta }$. It is apparent that a locally optimal point of the standard optimization problem (5) is also locally optimal for the hierarchical optimization problem (6, 7), if the point is within the allowed parameter boundaries for the optimization.

The formulation (6) might appear more involved, however, it possesses several properties which might be advantageous:

1. The individual dimensions of the inner and outer subproblems (6, 7) are lower than the dimension of the original problem (5).
2. The optimization of the inner subproblem does not require the repeated numerical simulation of the ODE model.
3. For several noise models, e.g. Gaussian and Laplace noise, the inner subproblem can be solved analytically.

If an analytical solution for the inner subproblem is available, the scaling parameters **s** and also the noise parameters σ can be calculated directly and the amount of parameters that need to be optimized iteratively reduces to *n_θ_*, which corresponds to alternative 2 in Figure 1D. In the following two sections, the analytic expressions for the Gaussian and Laplace noise are derived. For this, let observable index *i* be arbitrary but fixed.

**Analytic expressions for the optimal scaling and noise parameters for Gaussian noise**

In this study, we evaluated the scaling and noise parameters for Gaussian noise analytically. To derive the analytic expression for the optimal parameters, we exploited that the objective function for Gaussian noise,

$$J(\theta ,s,\sigma )=\frac{1}{2}\sum_{i,k}\;\text{log}\;(2\pi \sigma_{i}^{2})+{\left(\frac{{\bar{y}}_{i,k}-s_{i}\cdot h_{i}(x(t_{k},\theta ),\theta )}{\sigma_{i}}\right)}^{2}.$$

is continuously differentiable, and that the gradient of *J* at a local minimum is zero. For the inner subproblem this implies

$$\nabla_{s}J(\theta ,s,\sigma ){|}_{\hat{s},\hat{\text{σ}}}=0\;\text{and}\;\nabla_{\sigma }J(\theta ,s,\sigma ){|}_{\hat{s},\hat{\text{σ}}}=0.$$

These equations can be solved analytically (see Supplementary Section S1), which yields the unique optimal values

$$\begin{array}{c} {\hat{s}}_{i}(\theta )=\frac{\sum_{k}{\bar{y}}_{i,k}\cdot h_{i}(x(t_{k},\theta ),\theta )}{\sum_{k}h_{i}{\left(x(t_{k},\theta ),\theta \right)}^{2}}\;\\ {\hat{\sigma }}_{i}^{2}(\theta )=\frac{1}{n_{k}}\sum_{k}{\left({\bar{y}}_{i,k}-{\hat{s}}_{i}(\theta )\cdot h_{i}(x(t_{k},\theta ),\theta )\right)}^{2}\; \end{array}$$

with number of time points *n_k_*. Consistent with the structure of the hierarchical problem (6), both formulas depend only on the dynamic parameters θ.

In many studies (e.g. Bachmann *et al.*, 2011), observation functions of the form $\text{log}\;({\bar{y}}_{i,k})=\text{log}\;(s_{i}h_{i}(x(t_{k},\theta ),\theta ))+\epsilon_{i}$ are used. In the Supplementary Section S2, we provide the derivation of the corresponding optimal parameters.

**Analytic expressions for the optimal scaling and noise parameters for Laplace noise**

For Laplace noise the negative log-likelihood function is

(8) $$J(\theta ,s,\sigma )=\sum_{i,k}\;\text{log}\;(2\sigma_{i})+\frac{\left|{\bar{y}}_{i,k}-s_{i}\cdot h_{i}(x(t_{k},\theta ),\theta )\right|}{\sigma_{i}}.$$

This objective function is continuous but not continuously differentiable. In this case, a sufficient condition for a local minimum is that the right limit value of the derivative is negative and the left limit value is positive. The derivative of (8) with respect to *s_i_* can be written as

$$\frac{\partial J}{\partial s_{i}}=-\frac{1}{\sigma_{i}}\cdot \sum_{k}(|h_{i}(x(t_{k},\theta ),\theta )|\cdot \text{sgn}\left(\frac{{\bar{y}}_{i,k}}{h_{i}(x(t_{k},\theta ),\theta )}-s_{i}\right))\;,$$

**Fig. 2.**
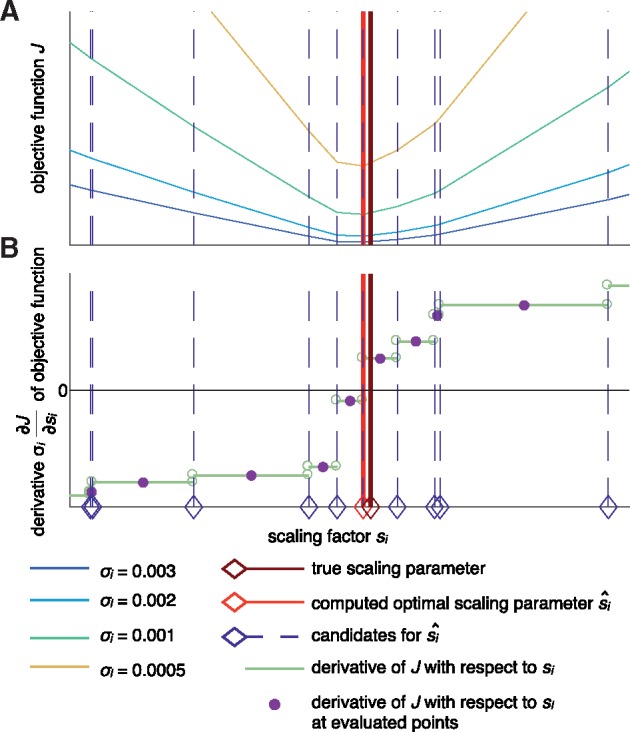
Illustration of the computation of an optimal scaling parameter ${\hat{s}}_{i}$ for Laplace noise. (**A**) Objective function *J* for fixed θ and different values of *σ_i_*, showing that the kinks, i.e. the points of non-differentiability, indicated by the dashed lines are independent of *σ_i_*. (**B**) Derivative of the objective function with respect to the scaling parameter which is not defined at the kinks. The light red and dark red lines indicate the computed scaling parameter and the true optimal scaling parameter, respectively

As *σ_i_* is positive, the locations of kinks in the objective function and the corresponding jumps in the derivative are independent of *σ_i_* (Fig. 2). Accordingly, the problem of finding ${\hat{s}}_{i}$ reduced to checking the signs of the derivative before and after the jump points $s_{i,k}={\bar{y}}_{i,k}/h_{i}(x(t_{k},\theta ),\theta )$. We sorted *s_i_*_,__*k*_ in increasing order and evaluated the derivatives at the midpoints between adjacent jumps, a procedure which is highly efficient as the ODE model does not have to be simulated. Given ${\hat{s}}_{i}$, the unique optimal noise parameter ${\hat{\sigma }}_{i}$ follows from the work of Norton (1984) as

$${\hat{\sigma }}_{i}(\theta )=\frac{1}{n_{k}}\sum_{k}(|h_{i}(x(t_{k},\theta ),\theta )|\;\cdot \left|\frac{{\bar{y}}_{i,k}}{h_{i}(x(t_{k},\theta ),\theta )}-{\hat{s}}_{i}(\theta )\right|).$$

Both derived formulas depend only on the dynamic parameters θ, in consistence with the structure of the hierarchical problem (6). In summary, we reformulated the original optimization problem (5) as a hierarchical optimization problem (6, 7), and provided an analytic solution to the inner subproblem (7) for several relevant cases. Using the analytic solutions, the kinetic parameters can be inferred by solving a lower-dimensional problem.

## 3 Results

To study and compare the performance of parameter estimation from relative data using the standard approach and our hierarchical approach, we applied both to three published estimation problems.

### 3.1 Models and experimental data

The considered models describe biological signaling pathways, namely, the JAK-STAT (Bachmann *et al.*, 2011; Swameye *et al.*, 2003) and the RAF/MEK/ERK signaling pathway (Fiedler *et al.*, 2016).

#### 3.1.1 JAK-STAT signaling I

**Fig. 3.**
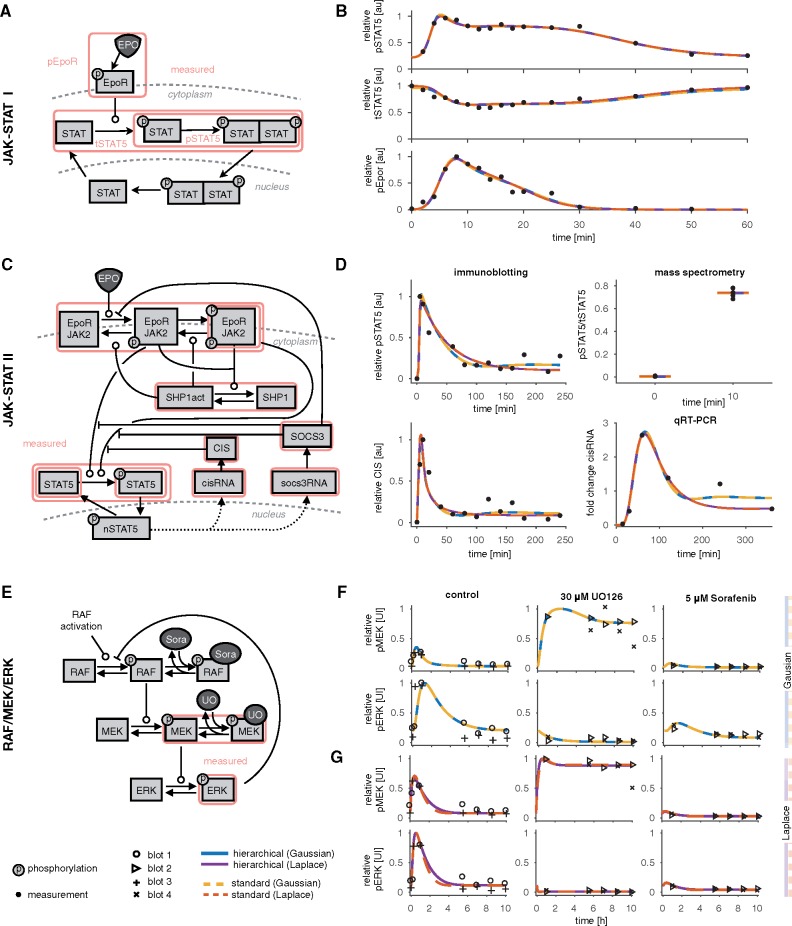
Models and experimental data. (**A, B**) JAK-STAT I. (**A**) Illustration of the model according to Swameye *et al.* (2003). Arrows represent biochemical reactions, and the observables of the model used are highlighted by boxes. (**B**) Experimental data and fitted trajectories for the best parameter found with multi-start local optimization with 100 starts. The results are shown for the standard (dotted lines) and hierarchical (solid lines) approach for optimization for Gaussian and Laplace noise. (**C, D**) JAK-STAT II. (**C**) Illustration of the model according to Bachmann *et al.* (2011). (**D**) Experimental data and fitted trajectories for the best parameter found with multi-start local optimization for 100 and 200 starts for Gaussian and Laplace noise, respectively. 33 out of 541 data points are shown. (**E**–**G**) RAF/MEK/ERK. (**E**) Illustration of the model according to Fiedler *et al.* (2016). (**F, G**) Experimental data and fitted trajectories for the best parameter found with multi-start local optimization for 500 and 1000 starts for Gaussian and Laplace noise, respectively. Different markers indicate the different blots. The data is scaled according to the estimated scaling parameters, yielding different visualizations for different parameters, as obtained with the Gaussian and the Laplace noise assumption. (**F**) Fitted trajectories for Gaussian noise for the standard (dotted line) and hierarchical (solid line) approach for optimization. (**G**) Fitted trajectories for Laplace noise

The first application example we considered is the model of Epo-induced JAK-STAT signaling introduced by Swameye *et al.* (2003) (Fig. 3A). Epo yields the phosphorylation of signal transducer and activator of transcription 5 (STAT5), which dimerizes, enters the nucleus to trigger the transcription of target genes, gets dephosphorylated, and is transported to the cytoplasm. We implemented the model which describes the phosphorylated Epo receptor concentration as a time-dependent spline (Schelker *et al.*, 2012). For further details on the model, we refer to Supplementary Section S5.1.

The model parameters were estimated using immunoblotting data for the phosphorylated Epo receptor (pEpoR), phosphorylated STAT5 (pSTAT5) and the total amount of STAT5 in the cytoplasm (tSTAT5) (Fig. 3B). In total 46 data points are available for 16 different time points. Since immunoblotting only provides relative data, the scaling parameters for the observables need to be estimated from the data. As proposed by Schelker *et al.* (2012), the scaling parameter for pEpoR has been fixed to avoid structural non-identifiabilities (Raue *et al.*, 2009). The model with the reduced parameter vector is structurally identifiable. This yields in total 16 parameters, which comprise *n_θ_* = 11 dynamic parameters (see Supplementary Section S5.1), *n_s_* = 2 scaling parameters and *n_σ_* = 3 noise parameters.

#### 3.1.2 JAK-STAT signaling II

The second application example is the model of JAK-STAT signaling introduced by Bachmann *et al.* (2011). This model provides more details compared to the previous one. It includes, for instance, gene expression of cytokine-inducible SH2-containing protein (CIS) and suppressor of cytokine signaling 3 (SOCS3), and possesses more state variables and parameters (Fig. 3C).

The model parameters were estimated using 541 data points collected by immunoblotting, qRT-PCR and quantitative mass spectrometry (Fig. 3D and Supplementary Fig. S4). To model the observables Bachmann *et al.* (2011) used *n_s_* = 43 scaling parameters, and *n_σ_* = 11 noise parameters, yielding *n_θ_* = 58 parameters of the outer subproblem of in total 112 parameters. Some scaling and noise parameters are shared between experiments and some are shared between observables. For this model, most of the observables were compared at the log_10_ scale (see Supplementary Section S5.2).

#### 3.1.3 RAF/MEK/ERK signaling

The third application example we considered is the model of RAF/MEK/ERK signaling introduced by Fiedler *et al.* (2016). The model describes the phosphorylation cascade and a negative feedback of phosphorylated ERK on RAF phosphorylation (Fig. 3E).

**Fig. 4.**
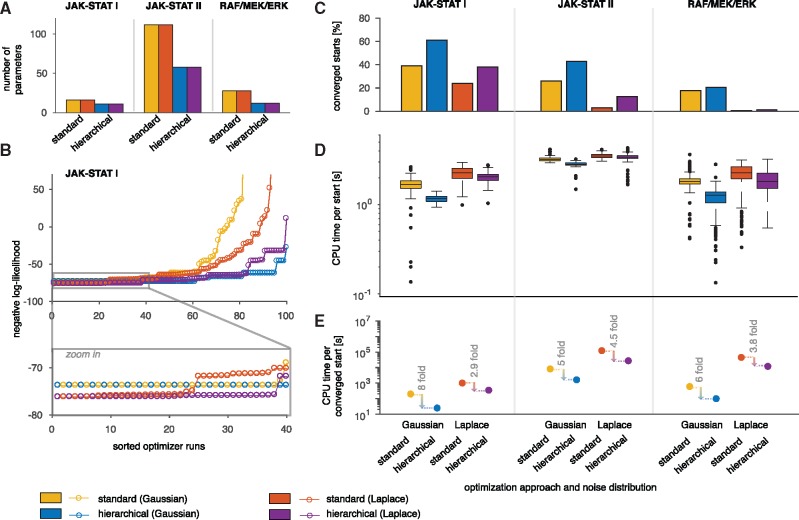
Evaluation of the standard and hierarchical approach for three application examples. (**A**) Number of optimization variables in the outer subproblem. (**B**) Likelihood waterfall plot for the JAK-STAT model I. The ascendingly sorted negative log-likelihood values are shown for both approaches (standard and hierarchical) and noise distributions (Gaussian and Laplace). (**C–E**) Comparison of the two optimization approaches and two noise distribution for the three models. (**C**) Percentage of converged starts over all performed local optimizations. (**E**) CPU time needed per converged start

Fiedler *et al.* (2016) collected Western blot data for HeLa cells for two observables, phosphorylated MEK and phosphorylated ERK, with four replicates at seven time points giving 72 data points (Fig. 3F and G). Each observable and replicate was assumed to have different scaling and noise parameters, yielding 16 additional parameters and in total 28 parameters in the standard approach (Fig. 4A).

### 3.2 Evaluation of the approaches

We performed parameter estimation for the application examples using the standard and the hierarchical approach. For each example, the case of Gaussian and Laplace noise was considered. The resulting optimization problems were solved with the MATLAB toolbox PESTO (Stapor *et al.*, 2018), using multi-start local optimization, an approach which was previously found to be computationally efficient and reliable (Raue *et al.*, 2013). Initial points were sampled uniformly within their parameter boundaries and local optimization was performed using the interior point method implemented in the MATLAB function fmincon.m for both noise distributions. However, alternatively other optimization methods can easily be employed. Numerical simulation and forward sensitivity analysis for gradient evaluation was performed using the MATLAB toolbox AMICI (Fröhlich *et al.*, 2017), which provides an interface to CVODES (Serban and Hindmarsh, 2005). To improve convergence and computational efficiency, log_10_-transformed parameters were used for the optimization.

#### 3.2.1 Qualitative comparison of optimization approaches for different noise distributions

As the standard and hierarchical approach should in principle be able to achieve the same fit, we first studied the agreement of trajectories for the optimal parameters. We found that they coincide for the JAK-STAT model I and II, for both noise distributions, and the RAF/MEK/ERK using Gaussian noise. This indicates that the hierarchical approach is able to find the same optimal likelihood value as the standard approach (Fig. 3B and D). Also the best likelihood values which were found by the two approaches are the same (Fig. 4B and Supplementary Fig. S5). For the RAF/MEK/ERK model with the assumption of Laplace distributed measurement noise, the fitted trajectories between the experimental data slightly deviate (Fig. 3F), which can be explained by convergence issues and broad confidence intervals of the parameters (Supplementary Fig. S8). As expected, there are differences between the results obtained with Gaussian and Laplace noise, which is visible in the trajectories and the corresponding likelihood values.

#### 3.2.2 Convergence of optimizers

As the performance of multi-start local methods depends directly on the convergence of the local optimizers, we assessed for how many starting points the local optimizer reached the best objective function value found across all runs. This was done by studying the likelihood waterfall plots (Fig. 4B). The number of converged starts is the number of starts for which the final objective function value is close to the best found objective function value (across all starts and optimization methods). The statistical threshold is defined according to a likelihood ratio test (Hross and Hasenauer, 2016). We found that the proposed hierarchical approach achieved consistently a higher fraction of converged starts than the standard approach (Fig. 4C). Local optimization using the hierarchical approach converged on average in 29.3% of the runs while the standard approach converged on average in 18.4% of the runs.

The application examples vary with respect to the total number of parameters and in the number of parameters which correspond to scaling or noise parameters (Fig. 4A). While for the JAK-STAT model I only five parameters could be optimized analytically, for the JAK-STAT model II almost half of the parameters correspond to scaling or noise parameters. Interestingly, even when the dimension of the outer optimization problem was only reduced by few parameters by solving the inner problem analytically, we observed a substantial increase of the percentage of converged multi-starts (Fig. 4C).

#### 3.2.3 Computational efficiency

As computation resources are often limiting, we finally analyzed the computation time per converged start. We found that on average the computation time per start was lower for the hierarchical approach than for the standard approach (Fig. 4D). The hierarchical approach is faster than the standard approach for a high fraction of the starts (Supplementary Fig. S1C). In combination with the improved convergence rate, this resulted in a substantially reduced computation time per converged start, aka a start which reached the minimal value observed across all starts (Fig. 4E). Given a fixed computational budget, the hierarchical approach achieved on average 5.06 times more optimization runs which reached the best objective function values than the standard approach. The expected improvement in terms of CPU time per converged start when using the hierarchical approach is in average $3.4\times 10^{3},\;5.8\times 10^{2}$ and $6.5\times 10^{4}$ seconds for JAK-STAT I, JAK-STAT II and RAF/MEK/ERK, respectively.

In summary, the application of our hierarchical approach to parameter estimation from relative data to the models shows consistently that our approach yields parameter values of the same quality as the standard method, while achieving better convergence and reducing the computation time substantially.

## 4 Conclusion

The statistically rigorous estimation of model parameters from relative data requires non-standard statistical models (Thomaseth and Radde, 2016) or scaling parameters (Raue *et al.*, 2013). Unfortunately, the former is not supported by established toolboxes and the latter increases the dimensionality of the estimation problem. In this manuscript, we introduced a hierarchical approach which avoids the increase of dimensionality and is applicable to a broad range of noise distributions. For Gaussian and Laplace noise we provided analytic expressions. The approach can be used for combinations of relative and absolute data, and for different optimization methods, including least-squares methods or global optimization methods such as particle swarm optimization (Vaz and Vicente, 2009) (see Supplementary Fig. S3) or GLSDC (Kimura and Konagaya, 2003). While the method effectively reduces the dimensionality of the optimization problem, optimal parameter values and parameter identifiability remains unchanged. Accordingly, it has to be kept in mind that the presence of scaling factors often results in structural non-identifiabilities and this problem is not solved by the hierarchical approach for optimization.

We evaluated the performance of our hierarchical approach and compared it to the standard approach for three models, which vary in their complexity. For all applications, we found that our hierarchical approach yielded fits of the same or better quality. In addition, convergence was improved and the computation time was shortened substantially. We demonstrated that our approach can also be used when relative and absolute data are modeled together in an experiment, and when several observables or experiments share scaling and/or noise parameters. This renders our approach applicable to a wide range of mathematical models studied in systems and computational biology. We provided a generic implementation of the objective function for the hierarchical approach for Gaussian and Laplace noise. The objective function is provided in the Supplementary Information (along with the rest of the code) and included in the MATLAB toolbox PESTO (Stapor *et al.*, 2018). As the hierarchical approach proposed in this study can easily be integrated in existing toolboxes, not only optimization but also profile calculation can be improved (Supplementary Figs S4 and S8).

For the considered models, we observed that the fraction of converged local optimization runs decreases as the model dimension increases. Potential reasons are that for larger models the region of attraction of the global optimum might be smaller and there might be more local minima. We also observed that fraction of converged starts is lower for Laplace noise than for Gaussian noise. This most probably occurs due to non-differentiabilities in the objective function, which complicate the optimization procedure. When using Laplace priors for parameters, the optimization routine can be adapted (Steiert *et al.*, 2016), however, this approach is not easily transferable to the use of Laplace noise as the switching points depend on the numerical solution of the ODE. Thus, further work should be directed towards implementing and testing appropriate optimization routines. Amongst others, local direct search optimizers (De La Maza and Yuret, 1994; Nelder and Mead, 1965), which are not gradient-based and therefore do not require differentiability, should be considered.

In addition to the scaling and noise parameters, also other parameters which only contribute to the mapping from the states to the observables, could be optimized analytically. This includes offset parameters, which are used to model background intensities or unspecific binding. Extending our approach to also calculate these parameters analytically would decrease the number of parameters in the outer optimization even more.

When using gradient-based optimization, further improvements could be achieved by extending the approach to scalable approaches to calculate the objective function gradient. In this manuscript, we employed forward sensitivities for the calculation of the objective function gradient. However, it has been shown that for large-scale models with a high number of parameters, adjoint sensitivities can reduce the computation time needed for simulation (Fröhlich *et al.*, 2017). Thus, a further promising approach would be the combination of both complementary approaches for the handling of large-scale models.

To summarize, employing our hierarchical approach for optimization yielded more robust results and speed up the computation time. This renders the approach valuable for estimating parameters from relative data. The proposed approach might facilitate the handling of large-scale models, which possess many measurement parameters.

## Funding

This work was supported by the European Union’s Horizon 2020 research and innovation program under Grant Agreement No. 686282.

*Conflict of Interest*: none declared.

## Supplementary Material

Supplementary Data
