# Supplementary material for: Hierarchical optimization for the efficient parametrization of ODE models
Source: Bioinformatics. 2018 Jul 13;34(24):4266–73. doi: 10.1093/bioinformatics/bty514 (PMC6289139; doi:10.1093/bioinformatics/bty514)
Supplement: Supplementary Data [file bty514_supp.zip › bty514-suppl_data/bty514_Supplementary_Information.pdf]

Supplementary Information

Hierarchical optimization for the efficient parametrization of ODE models

Carolin Loos [1,*], Sabrina Krause [1,*], and Jan Hasenauer [1,†]

[1]Helmholtz Zentrum München - German Research Center for Environmental Health, Institute of Computational Biology, 85764 Neuherberg, Germany, and Technische Universität München, Center for Mathematics, Chair of Mathematical Modeling of Biological Systems, 85748 Garching, Germany,

[*]C.L. and S.K. contributed equally to this work.

[†]To whom correspondence should be addressed.

Contents

1 General formula for analytic scaling and noise parameters **2**

1.1 Gaussian noise 2

1.2 Laplace noise 5

2 Comparison of data and simulation at a logarithmic scale **6**

2.1 Gaussian noise 6

2.2 Laplace noise 7

3 Profile likelihood calculation **8**

4 Implementation **8**

5 Models and experimental data **9**

5.1 JAK-STAT signaling I 9

5.2 JAK-STAT signaling II 13

5.3 RAF/MEK/ERK signaling 22

6 Normalization of relative data **24**

1 General formula for analytic scaling and noise parameters

In the main manuscript, we covered experimental data sets which have different time points. Here, we provide the derivation of the expressions for the general case, in which the experimental data also comprise different replicates, experiments, and conditions, e.g., varying drug doses. We considered that the ODE system also depends on an input $\mathbf{u} \in \mathbb{R}^{n_u}$,

$$\dot{\mathbf{x}} = \mathbf{f}(\mathbf{x}(t,\boldsymbol{\theta}),\boldsymbol{\theta},\mathbf{u}), \quad \mathbf{x}(t_0,\boldsymbol{\theta},\mathbf{u}) = x_0(\boldsymbol{\theta},\mathbf{u})\,, \tag{1}$$

thus, $f\colon \mathbb{R}^{n_x} \times \mathbb{R}^{n_\theta} \times \mathbb{R}^{n_u} \to \mathbb{R}^{n_x}$, which also affects the mapping to the observables

$$\mathbf{y}(t,\boldsymbol{\theta},\mathbf{u}) = \mathbf{h}(\mathbf{x}(t,\boldsymbol{\theta}),\boldsymbol{\theta},\mathbf{u})\,. \tag{2}$$

The experimental data is then given by

$$\mathcal{D} = \left\{ \left\{ \left\{ \{ \bar{\mathbf{y}}_{k,r,c_e}, t_{k,c_e}, \mathbf{u}_{c_e} \}_k \right\}_r \right\}_{c_e \in I_e} \right\}_e\,, \tag{3}$$

including all indices for time point k, replicate r, experiment-specific condition c_e, and experiment e. The indices I_e indicate which conditions correspond to a certain experiment. The measurements are mapped to the states by

$$\bar{y}_{i,k,r,c_e} = s_{i,r,c_e} \cdot h_i^e(\mathbf{x}(t_{k,c_e},\boldsymbol{\theta}),\boldsymbol{\theta},\mathbf{u}_{c_e}) + \varepsilon_{i,k,r,c_e}\,,$$

with $\varepsilon_{i,k,r,c_e} \sim \mathcal{N}(0,\sigma_{i,r,c_e}^2)$ or $\varepsilon_{i,k,r,c_e} \sim \text{Laplace}(0,\sigma_{i,r,c_e})$, and $s_{i,r,c_e} = 1$ for absolute measurements. Also, the structure of the mapping from states to observables might be experiment-specific. The negative log-likelihood is given by

$$J(\boldsymbol{\theta},\mathbf{s},\boldsymbol{\sigma}) = -\sum_{e,i,k,r} \sum_{c_e \in I_e} \log p(\bar{y}_{i,k,r,c_e} | s_{i,r,c_e} \cdot h_i^e(\mathbf{x}(t_{k,c_e},\boldsymbol{\theta}),\boldsymbol{\theta},\mathbf{u}_{c_e}), \sigma_{i,r,c_e})\,. \tag{4}$$

In the main manuscript, we presented the analytic formulas for the case that each observable and corresponding replicate has different scaling and noise parameters, but that these parameters do not change between conditions and time points. A more general formula is provided in the following, covering, e.g., the case that replicates share the same scaling parameters, but observables do not. This can be easily generalized to also include variability between time points.

1.1 Gaussian noise

The general objective function under Gaussian noise is given by

$$J(\boldsymbol{\theta},\mathbf{s},\boldsymbol{\sigma}) = \frac{1}{2} \sum_{i,r,k,e} \sum_{c_e \in I_e} \log(2\pi {\sigma_{i,r,c_e}}^2) + \left(\frac{\bar{y}_{i,k,r,c_e} - s_{i,r,c_e} \cdot h_i^e(\mathbf{x}(t_{k,c_e},\boldsymbol{\theta}),\boldsymbol{\theta},\mathbf{u}_{c_e})}{\sigma_{i,r,c_e}} \right)^2\,. \tag{5}$$

To define which replicates, observables, and experiments share a scaling or noise parameter, we define

$$I_s^{i_s}, I_\sigma^{i_\sigma} \subset \mathbb{N}_+^{n_y} \times \mathbb{N}_+^{n_r} \times \mathbb{N}_+^{n_e}\,,$$

for $i_s = 1, \dots, n_s$ and $i_\sigma = 1, \dots, n_\sigma$. The number of replicates is denoted by n_r and the number of experiments by n_e. This means, all scaling parameters s_{i^*,r^*,c_e^*} for which the indices (i^*, r^*, e^*) are part of the same group I_s share the same scaling parameters. This yields n_s different scaling parameters that are estimated from the data. For this we denote $I_s^{i_s}(i^*, r^*, c_e^*)$ the group which includes the indices (i^*, r^*, c_e^*). This is analogously for the noise parameters. The derivative of the objective function with respect to a scaling parameter thus reads

$$\frac{\partial J}{\partial s_{i^*,r^*,c_e^*}} = \frac{1}{2} \sum_{k,e} \sum_{\substack{(i,r,c_e) \in \\ I_s^{i_s}(i^*,r^*,c_e^*)}} \frac{2}{\sigma^2_{i,r,c_e}} (\bar{y}_{i,k,r,c_e} - s_{i^*,r^*,c_e^*} \cdot h_i^e(\mathbf{x}(t_{k,c_e}, \boldsymbol{\theta}), \boldsymbol{\theta}, \mathbf{u}_{c_e})) \cdot (-h_i^e(\mathbf{x}(t_{k,c_e}, \boldsymbol{\theta}), \boldsymbol{\theta}, \mathbf{u}_{c_e})) \stackrel{!}{=} 0\,, \tag{6}$$

and was set to zero to obtain the analytic expression for the optimal scaling parameter. The solution does not depend on the noise parameters if $I_s^{i_s} \subset I_\sigma^{i_\sigma}$ $\forall i_s$, and we solve the equation with respect to s_{i^*,r^*,c_e^*} to obtain the optimal value

$$\hat{s}_{i^*,r^*,c_e^*} = \frac{\sum\limits_{k,e} \sum\limits_{\substack{(i,r,c_e) \in \\ I_s^{i_s}(i^*,r^*,c_e^*)}} \bar{y}_{i,k,r,c_e} \cdot h_i^e(\mathbf{x}(t_{k,c_e}, \boldsymbol{\theta}), \boldsymbol{\theta}, \mathbf{u}_{c_e})}{\sum\limits_{k,e} \sum\limits_{\substack{(i,r,c_e) \in \\ I_s^{i_s}(i^*,r^*,c_e^*)}} h_i^e(\mathbf{x}(t_{k,c_e}, \boldsymbol{\theta}), \boldsymbol{\theta}, \mathbf{u}_{c_e})^2}\,.$$

If there exists some $h_i^e(\mathbf{x}(t_{k,c_e}, \boldsymbol{\theta}), \boldsymbol{\theta}, \mathbf{u}_{c_e}) \neq 0$, then

$$\frac{\partial^2 J}{\partial^2 s_{i^*,r^*,c_e^*}} = \sum_{k,e} \sum_{\substack{(i,r,c_e) \in \\ I_s^{i_s}(i^*,r^*,c_e^*)}} \frac{h_i^e(\mathbf{x}(t_{k,c_e}, \boldsymbol{\theta}), \boldsymbol{\theta}, \mathbf{u}_{c_e})^2}{\sigma^2_{i,r,c_e}} > 0.$$

This yields that $\hat{s}_{i^*,r^*,c_e^*}$ is the unique optimal scaling parameter, which minimizes (5), for a given set of dynamic parameters $\boldsymbol{\theta}$. If $\forall i, k, c_e : h_i^e(\mathbf{x}(t_{k,c_e}, \boldsymbol{\theta}), \boldsymbol{\theta}, \mathbf{u}_{c_e}) = 0$, the scaling parameter does not have an effect on the objective function.

For the noise parameters, we need

$$\frac{\partial J}{\partial \sigma^2_{i^*,r^*,c_e^*}} = \frac{1}{\sigma^2_{i^*,r^*,c_e^*}} \cdot \sum_{k,e} \sum_{\substack{(i,r,c_e) \in \\ I_\sigma^{i_\sigma}(i^*,r^*,c_e^*)}} 1 - \left(\frac{\bar{y}_{i,k,r,c_e} - s_{i,r,c_e} \cdot h_i^e(\mathbf{x}(t_{k,c_e}, \boldsymbol{\theta}), \boldsymbol{\theta}, \mathbf{u}_{c_e})}{\sigma_{i^*,r^*,c_e^*}} \right)^2 \stackrel{!}{=} 0\,. \tag{7}$$

We write

$$
\begin{aligned}
&\frac{1}{\sigma^2_{i^*,r^*,c^*_e}} \cdot \sum_{k,e} \sum_{\substack{(i,r,c_e)\in\\ I^{i\sigma}_{\sigma}(i^*,r^*,c^*_e)}} 1 - \left(\frac{\bar{y}_{i,k,r,c_e} - s_{i,r,c_e} h^e_i(\mathbf{x}(t_{k,c_e},\boldsymbol{\theta}),\boldsymbol{\theta},\mathbf{u}_{c_e})}{\sigma_{i^*,r^*,c^*_e}}\right)^2 = 0 \\
\Leftrightarrow\quad & \sigma^2_{i^*,r^*,c^*_e} \cdot \sum_{k,e} \sum_{\substack{(i,r,c_e)\in\\ I^{i\sigma}_{\sigma}(i^*,r^*,c^*_e)}} 1 = \sum_{k,e} \sum_{\substack{(i,r,c_e)\in\\ I^{i\sigma}_{\sigma}(i^*,r^*,c^*_e)}} \left(\bar{y}_{i,k,r,c_e} - s_{i,r,c_e}\cdot h^e_i(\mathbf{x}(t_{k,c_e},\boldsymbol{\theta}),\boldsymbol{\theta},\mathbf{u}_{c_e})\right)^2 \\
\Rightarrow\quad & \hat{\sigma}^2_{i^*,r^*c^*_e} = \frac{1}{\underbrace{\sum_{k,e} \sum_{\substack{(i,r,c_e)\in\\ I^{i\sigma}_{\sigma}(i^*,r^*,c^*_e)}} 1}_{(\dagger)}} \sum_{k,e} \sum_{\substack{(i,r,c_e)\in\\ I^{i\sigma}_{\sigma}(i^*,r^*,c^*_e)}} \left(\bar{y}_{i,k,r,c_e} - s_{i,r,c_e}\cdot h^e_i(\mathbf{x}(t_{k,c_e},\boldsymbol{\theta}),\boldsymbol{\theta},\mathbf{u}_{c_e})\right)^2 ,
\end{aligned}
$$

in which (†), the nominator, is simply the number of observations in which σ_{i^*,r^*,c^*_e} appears. In some cases, for instance if all experiments share the same scaling parameter, we neglected the superscript e.

Since

$$
\begin{aligned}
\frac{\partial^2 J(\boldsymbol{\theta},\mathbf{s},\hat{\boldsymbol{\sigma}})}{\partial^2\sigma^2_{i^*,r^*,c^*_e}} &= \sum_{k,e} \sum_{\substack{(i,r,c_e)\in\\ I^{i\sigma}_{\sigma}(i^*,r^*,c^*_e)}} \frac{2\left(\bar{y}_{i,k,r,c_e} - s_{i,r,c_e}\cdot h^e_i(\mathbf{x}(t_{k,c_e},\boldsymbol{\theta}),\boldsymbol{\theta},\mathbf{u}_{c_e})\right)^2 - \hat{\sigma}^2_{i^*,r^*,c^*_e}}{\hat{\sigma}^6_{i^*,r^*,c^*_e}} \\
&= \frac{1}{\hat{\sigma}^6_{i^*,r^*,c^*_e}} \left(\sum_{k,e} \sum_{\substack{(i,r,c_e)\in\\ I^{i\sigma}_{\sigma}(i^*,r^*,c^*_e)}} 2\left(\bar{y}_{i,k,r,c_e} - s_{i,r,c_e}\cdot h^e_i(\mathbf{x}(t_{k,c_e},\boldsymbol{\theta}),\boldsymbol{\theta},\mathbf{u}_{c_e})\right)^2 \right. \\
&\qquad \left. - \hat{\sigma}^2_{i^*,r^*,c^*_e} \cdot \sum_{k,e} \sum_{\substack{(i,r,c_e)\in\\ I^{i\sigma}_{\sigma}(i^*,r^*,c^*_e)}} 1 \right) \\
&= \frac{1}{\hat{\sigma}^6_{i^*,r^*,c^*_e}} \left(2\hat{\sigma}^2_{i^*,r^*,c^*_e} \cdot \sum_{k,e} \sum_{\substack{(i,r,c_e)\in\\ I^{i\sigma}_{\sigma}(i^*,r^*,c^*_e)}} 1 - \hat{\sigma}^2_{i^*,r^*,c^*_e} \cdot \sum_{k,e} \sum_{\substack{(i,r,c_e)\in\\ I^{i\sigma}_{\sigma}(i^*,r^*,c^*_e)}} 1 \right) \\
&= \frac{2}{\hat{\sigma}^6_{i^*,r^*,c^*_e}} > 0,
\end{aligned}
$$

the noise parameter $\hat{\sigma}^2_{i^*,r^*,c^*_e}$ is the unique parameter minimizing (5).

The gradient used for optimization is given by

$$
\frac{\partial J}{\partial \theta} = \sum_{i,r,k,e} \sum_{c_e \in I_e} \frac{\bar{y}_{i,k,r,c_e} - \hat{s}_{i,r,c_e}\cdot h^e_i(\mathbf{x}(t_{k,c_e},\boldsymbol{\theta}),\boldsymbol{\theta},\mathbf{u}_{c_e})}{\hat{\sigma}^2_{i,r,c_e}} \cdot \hat{s}_{i,r,c_e} \cdot \frac{\partial h^e_i(\mathbf{x}(t_{k,c_e},\boldsymbol{\theta}),\boldsymbol{\theta},\mathbf{u}_{c_e})}{\partial \theta},
$$

for which $\frac{\partial h^e_i(\mathbf{x}(t_{k,c_e},\boldsymbol{\theta}),\boldsymbol{\theta},\mathbf{u}_{c_e})}{\partial \theta}$ is obtained by forward sensitivity equations employed in AMICI. Moreover,

$$
\frac{\partial J}{\partial s} = 0, \quad \frac{\partial J}{\partial \sigma} = 0\,,
$$

because of (6) and (7). The Hessian with respect to the dynamic parameters is

$$\frac{\partial^2 J}{\partial\theta_j\partial\theta_l} = \sum_{i,r,k,e}\sum_{c_e\in I_e}\left(\frac{\hat{s}_{i,r,c_e}}{\hat{\sigma}_{i,r,c_e}}\right)^2 \cdot \frac{\partial h_i^e(\mathbf{x}(t_{k,c_e},\boldsymbol{\theta}),\boldsymbol{\theta},\mathbf{u}_{c_e})}{\partial\theta_j}\cdot\frac{\partial h_i^e(\mathbf{x}(t_{k,c_e},\boldsymbol{\theta}),\boldsymbol{\theta},\mathbf{u}_{c_e})}{\partial\theta_l} + \underbrace{\frac{\bar{y}_{i,k,r,c_e} - \hat{s}_{i,r,c_e}\cdot h_i^e(\mathbf{x}(t_{k,c_e},\boldsymbol{\theta}),\boldsymbol{\theta},\mathbf{u}_{c_e})}{\hat{\sigma}^2_{i,r,c_e}}\cdot\hat{s}_{i,r,c_e}\cdot\frac{\partial^2 h_i^e(\mathbf{x}(t_{k,c_e},\boldsymbol{\theta}),\boldsymbol{\theta},\mathbf{u}_{c_e})}{\partial\theta_j\partial\theta_l}}_{(*)}.$$

For the remaining parameters, the Hessian is zero. We implemented an approximation of the Hessian neglecting the terms $(*)$ that include higher-order sensitivities.

1.2 Laplace noise

Laplace noise provides more reliable parameter estimates in the case of outlier-corrupted data, without having problems of overfitting the data in the case of limited amount of data, as, e.g., Student's t noise (Maier et al., 2017). For Laplace noise, the expression for the optimal scaling and noise parameters can be generalized analogously to Section 1.1. The objective function for the general case is

$$J(\boldsymbol{\theta},\mathbf{s},\boldsymbol{\sigma}) = \sum_{i,k,r,e}\sum_{c_e\in I_e}\log(2\sigma_{i,r,c_e}) + \frac{|\bar{y}_{i,k,r,c_e} - s_{i,r,c_e}\cdot h_i^e(\mathbf{x}(t_{k,c_e},\boldsymbol{\theta}),\boldsymbol{\theta},\mathbf{u}_{c_e})|}{\sigma_{i,r,c_e}}. \tag{8}$$

The derivative with respect to a scaling parameter is

$$\frac{\partial J}{\partial s_{i^*,r^*,c_e^*}} = -\sum_{k,e}\sum_{\substack{(i,r,c_e)\in\\ I_s^{is}(i^*,r^*,c_e^*)}}\frac{1}{\sigma_{i,r,c_e}}\cdot\left(|h_i^e(\mathbf{x}(t_{k,c_e},\boldsymbol{\theta}),\boldsymbol{\theta},\mathbf{u}_{c_e})|\cdot\operatorname{sgn}\left(\frac{\bar{y}_{i,k,r,c_e}}{h_i^e(\mathbf{x}(t_{k,c_e},\boldsymbol{\theta}),\boldsymbol{\theta},\mathbf{u}_{c_e})} - s_{i^*,r^*,c_e^*}\right)\right)$$

with jump points

$$\left\{\left\{s_{i,k,r,c_e} = \frac{\bar{y}_{i,k,r,c_e}}{h_i^e(\mathbf{x}(t_{k,c_e},\boldsymbol{\theta}),\boldsymbol{\theta},\mathbf{u}_{c_e})}\right\}_{(i,r,c_e)\in I_s^{is}|_{(i^*,r^*,c_e^*)\in I_s^{is}}}\right\}_{k,e}. \tag{9}$$

These jump points are the candidates for the optimal scaling parameter and the candidate for which the sign of the derivative changes is chosen. For the optimal noise parameter we have

$$\frac{\partial J}{\partial\sigma_{i^*,r^*,c_e^*}} = \frac{1}{\sigma_{i^*,r^*,c_e^*}}\cdot\sum_{k,e}\sum_{\substack{(i,r,c_e)\in\\ I_\sigma^{i\sigma}(i^*,r^*,c_e^*)}}\left(1 - \frac{|\bar{y}_{i,k,r,c_e} - \hat{s}_{i,r,c_e}\cdot h_i^e(\mathbf{x}(t_{k,c_e},\boldsymbol{\theta}),\boldsymbol{\theta},\mathbf{u}_{c_e})|}{\sigma_{i^*,r^*,c_e^*}}\right) \stackrel{!}{=} 0 \tag{10}$$

$$\hat{\sigma}_{i^*,r^*,c_e^*} = \frac{1}{\sum_{k,e}\sum_{\substack{(i,r,c_e)\in\\ I_\sigma^{i\sigma}(i^*,r^*,c_e^*)}} 1}\cdot \sum_{k,e}\sum_{\substack{(i,r,c_e)\in\\ I_\sigma^{i\sigma}(i^*,r^*,c_e^*)}}\left(|h_i^e(\mathbf{x}(t_{k,c_e},\boldsymbol{\theta}),\boldsymbol{\theta},\mathbf{u}_{c_e})|\cdot\left|\frac{\bar{y}_{i,k,r,c_e}}{h_i^e(\mathbf{x}(t_{k,c_e},\boldsymbol{\theta}),\boldsymbol{\theta},\mathbf{u}_{c_e})} - \hat{s}_{i^*,r^*,c_e^*}\right|\right).$$

Analogously to Section 1.1 it can be shown that $\hat{\sigma}^2_{i^*,r^*,c_e^*}$ minimizes (8).

The gradient used for optimization is given by

$$\frac{\partial J}{\partial \theta} = -\sum_{i,r,k,e}\sum_{c_e \in I_e} \frac{\operatorname{sgn}\left(\bar{y}_{i,k,r,c_e} - \hat{s}_{i,r,c_e} \cdot h_i^e(\mathbf{x}(t_{k,c_e},\boldsymbol{\theta}),\boldsymbol{\theta},\mathbf{u}_{c_e})\right)}{\hat{\sigma}_{i,r,c_e}} \cdot \left(\hat{s}_{i,r,c_e} \cdot \frac{\partial h_i^e(\mathbf{x}(t_{k,c_e},\boldsymbol{\theta}),\boldsymbol{\theta},\mathbf{u}_{c_e})}{\partial \theta} + h_i^e(\mathbf{x}(t_{k,c_e},\boldsymbol{\theta}),\boldsymbol{\theta},\mathbf{u}_{c_e}) \cdot \frac{\partial \hat{s}_{i,r,c_e}}{\partial \theta}\right),$$

for which $\frac{\partial h_i^e(\mathbf{x}(t_{k,c_e},\boldsymbol{\theta}),\boldsymbol{\theta},\mathbf{u}_{c_e})}{\partial \theta}$ is obtained by forward sensitivity equations employed in AMICI. Moreover, $\frac{\partial J}{\partial \sigma} = 0$, because of (10).

2 Comparison of data and simulation at a logarithmic scale

In the main manuscript and Supplementary Information, Section 1, we provided the formulas for the comparison of data and simulation on a linear scale. However, sometimes it might be more appropriate to compare experimental data and simulation on a logarithmic scale.

2.1 Gaussian noise

For Gaussian noise, the objective function for the comparison on the logarithmic scale is given by

$$J(\boldsymbol{\theta},\mathrm{s},\boldsymbol{\sigma}) = \frac{1}{2}\sum_{i,k,r,e}\sum_{c_e \in I_e} \log(2\pi{\sigma_{i,r,c_e}}^2) + \left(\frac{\log(\bar{y}_{i,k,r,c_e}) - \log\left(s_{i,r,c_e} \cdot h_i^e\left(\mathbf{x}\left(t_{k,c_e},\boldsymbol{\theta}\right),\boldsymbol{\theta},\mathbf{u}_{c_e}\right)\right)}{\sigma_{i,r,c_e}}\right)^2$$

Thus, the derivative with respect to the scaling parameters is

$$\frac{\partial J}{\partial s_{i^*,r^*,c_e^*}} = \frac{1}{2}\sum_{k,e}\sum_{\substack{(i,r,c_e)\in \\ I_s^{is}(i^*,r^*,c_e^*)}} \frac{2\left(\log(\bar{y}_{i,k,r,c_e}) - \log(s_{i^*,r^*,c_e^*}) - \log(h_i^e\left(\mathbf{x}\left(t_{k,c_e},\boldsymbol{\theta}\right),\boldsymbol{\theta},\mathbf{u}_{c_e}\right))\right)\frac{1}{s_{i^*,r^*,c_e^*}}}{{\sigma_{i,r}^e}^2}.$$

This yields the formula for the optimal scaling parameters

$$\hat{s}_{i^*,r^*,c_e^*} = \exp\left(\frac{\sum\limits_{k,e}\sum\limits_{\substack{(i,r,c_e)\in \\ I_s^{is}(i^*,r^*,c_e^*)}} \log(\bar{y}_{i,k,r,c_e}) - \log(h_i^e\left(\mathbf{x}\left(t_{k,c_e},\boldsymbol{\theta}\right),\boldsymbol{\theta},\mathbf{u}_{c_e}\right))}{\sum\limits_{k,e}\sum\limits_{\substack{(i,r,c_e)\in \\ I_s^{is}(i^*,r^*,c_e^*)}} 1}\right) \tag{11}$$

and

$$\hat{\sigma}^2_{i^*,r^*,c_e^*} = \frac{1}{\sum\limits_{k,e}\sum\limits_{\substack{(i,r,c_e)\in \\ I_\sigma^{i\sigma}(i^*,r^*,c_e^*)}} 1} \cdot \sum_{k,e}\sum_{\substack{(i,r,c_e)\in \\ I_\sigma^{i\sigma}(i^*,r^*,c_e^*)}} \left(\log(\bar{y}_{i,k,r,c_e}) - \log(\hat{s}_{i^*,r^*,c_e^*} \cdot h_i^e\left(\mathbf{x}\left(t_{k,c_e},\boldsymbol{\theta}\right),\boldsymbol{\theta},\mathbf{u}_{c_e}\right)\right)^2. \tag{12}$$

The gradient used for optimization is given by

$$\frac{\partial J}{\partial \theta} = \sum_{i,r,k,e} \sum_{c_e \in I_e} 2 \cdot \frac{\log(\bar{y}_{i,k,r,c_e}) - \log(\hat{s}_{i,r,c_e} \cdot h_i^e(\mathbf{x}(t_{k,c_e}, \boldsymbol{\theta}), \boldsymbol{\theta}, \mathbf{u}_{c_e}))}{\hat{\sigma}^2_{i,r,c_e}} \cdot \frac{1}{h_i^e(\mathbf{x}(t_{k,c_e}, \boldsymbol{\theta}), \boldsymbol{\theta}, \mathbf{u}_{c_e})} \cdot \frac{\partial h_i^e(\mathbf{x}(t_{k,c_e}, \boldsymbol{\theta}), \boldsymbol{\theta}, \mathbf{u}_{c_e})}{\partial \theta} .$$

If the data is compared at $\log_{10}$ scale, as, e.g., for the JAK-STAT signaling model proposed by Bachmann et al. (2011), the negative log-likelihood function reads

$$J(\boldsymbol{\theta}, \mathbf{s}, \boldsymbol{\sigma}) = \frac{1}{2} \sum_{i,k,r,e} \sum_{c_e \in I_e} \log(2\pi {\sigma_{i,r,c_e}}^2) + \left(\frac{\log_{10}(\bar{y}_{i,k,r,c_e}) - \log_{10}\left(s_{i,r,c_e} \cdot h_i^e\left(\mathbf{x}\left(t_{k,c_e}, \boldsymbol{\theta}\right), \boldsymbol{\theta}, \mathbf{u}_{c_e}\right)\right)}{\sigma_{i,r,c_e}} \right)^2 .$$

The optimal scaling parameters here are the same as when using the natural logarithm (11). For the optimal noise parameters the log is replaced by $\log_{10}$ in (12).

2.2 Laplace noise

For the Laplace distribution including the logarithmic comparison

$$J(\boldsymbol{\theta}, \mathbf{s}, \boldsymbol{\sigma}) = \sum_{i,k,r,e} \sum_{c_e \in I_e} \log(2\sigma_{i,r,c_e}) + \frac{\left|\log(\bar{y}_{i,k,r,c_e}) - \log\left(s_{i,r,c_e} \cdot h_i^e\left(\mathbf{x}\left(t_{k,c_e}, \boldsymbol{\theta}\right), \boldsymbol{\theta}, \mathbf{u}_{c_e}\right)\right)\right|}{\sigma^e_{i,r}}$$

the same procedure can be applied for the logarithmic scale as for the linear scale, with the same set of candidate scaling parameters (9) as for the linear scale. However, one has to pay attention to adapt the derivative properly, for which the change of signs is checked. The optimal noise parameters then is given by

$$\hat{\sigma}_{i^*,r^*,c_e^*} = \frac{1}{\sum\limits_{k,e} \sum\limits_{\substack{(i,r,c_e) \in \\ I_\sigma^{i\sigma}(i^*,r^*,c_e^*)}} 1} \cdot \sum_{k,e} \sum_{\substack{(i,r,c_e) \in \\ I_\sigma^{i\sigma}(i^*,r^*,c_e^*)}} \left(\left|\log\left(h_i^e\left(\mathbf{x}\left(t_{k,c_e}, \boldsymbol{\theta}\right), \boldsymbol{\theta}, \mathbf{u}_{c_e}\right)\right)\right| \cdot \left| \frac{\log(\bar{y}_{i,k,r,c_e})}{\log(h_i^e(\mathbf{x}(t_{k,c_e}, \boldsymbol{\theta}), \boldsymbol{\theta}, \mathbf{u}_{c_e}))} - \hat{s}_{i^*,r^*,c_e^*} \right| \right).$$

The gradient used for optimization is given by

$$\frac{\partial J}{\partial \theta} = - \sum_{i,r,k,e} \sum_{c_e \in I_e} \frac{\operatorname{sgn}\left(\log(\bar{y}_{i,k,r,c_e}) - \log(\hat{s}_{i,r,c_e} \cdot h_i^e(\mathbf{x}(t_{k,c_e}, \boldsymbol{\theta}), \boldsymbol{\theta}, \mathbf{u}_{c_e}))\right)}{\hat{\sigma}_{i,r,c_e}} \cdot \left(\frac{1}{h_i^e(\mathbf{x}(t_{k,c_e}, \boldsymbol{\theta}), \boldsymbol{\theta}, \mathbf{u}_{c_e})} \cdot \frac{\partial h_i^e(\mathbf{x}(t_{k,c_e}, \boldsymbol{\theta}), \boldsymbol{\theta}, \mathbf{u}_{c_e})}{\partial \theta} + \frac{1}{\hat{s}_{i,r,c_e}} \cdot \frac{\partial \hat{s}_{i,r,c_e}}{\partial \theta} \right) .$$

3 Profile likelihood calculation

The uncertainty of parameter estimates was evaluated using profile likelihoods (Raue et al., 2009). The profile likelihood for parameter θ_i is given by

$$\begin{aligned}\mathrm{PL}(\theta_i) &= \max_{\theta_{j\neq i}} \mathcal{L}(\boldsymbol{\theta}, \mathrm{s}, \boldsymbol{\sigma}) \\ &= \exp\left(-\min_{\theta_{j\neq i},\mathbf{s},\boldsymbol{\sigma}} J(\boldsymbol{\theta}, \mathrm{s}, \boldsymbol{\sigma})\right).\end{aligned}$$

Employing the hierarchical approach for optimization for the calculation of the profile likelihood, we obtain

$$\begin{aligned}\mathrm{PL}(\theta_i) =& \exp\left(-\min_{\theta_{j\neq i}} J(\boldsymbol{\theta}, \hat{\mathrm{s}}(\boldsymbol{\theta}), \hat{\boldsymbol{\sigma}}(\boldsymbol{\theta}))\right) \\ &\text{with } (\hat{\mathrm{s}}(\boldsymbol{\theta}), \hat{\boldsymbol{\sigma}}(\boldsymbol{\theta})) = \operatorname*{argmin}_{\mathbf{s},\boldsymbol{\sigma}} J(\boldsymbol{\theta}, \mathrm{s}, \boldsymbol{\sigma}).\end{aligned}$$

The profile likelihoods employing the hierarchical approach are the same as for the standard approach if the scaling and noise parameters are unconstrained, since the profile comprises only objective function values of optimal scaling and noise parameter. As shown before, the optimal values are the same for the standard and the hierarchical approach and thus also the objective function and the resulting profile likelihoods are the same. If the gradient in the standard approach is zero, also the gradient in the hierarchical approach is zero due to the uniqueness of the optimal values calculated in the hierarchical approach.

4 Implementation

We implemented the log-likelihood function and the analytic calculation of the scaling and noise parameters in easy-to-use MATLAB functions. The log-likelihood function is provided in `loglikelihoodHierarchical.m`, which provides the log-likelihood value, the gradient of the log-likelihood function with respect to the dynamic parameters, and in the case of Gaussian noise also an approximation to the Hessian by neglecting second-order derivatives. The functions and examples are incorporated in the toolbox PESTO (Stapor et al., 2018b) and can be found on GitHub: `http://github.com/ICB-DCM/PESTO`. This toolbox employs the MATLAB function `fmincon`. We use the interior-point algorithm, which stops the optimization if a threshold for the norm of the gradient, the size of a step, the change in the objective function value or a maximal number of iterations reached. For the profile calculation we used the interior-point and trust-region-reflective algorithm as proposed by Stapor et al. (2018a). The simulated observables, their sensitivities, the experimental data, and the specification of measurement noise, scale of comparison between simulation and data, and shared parameters needs to be supplied by the user.

For our analysis, we employed the toolbox AMICI (Fröhlich et al., 2017) for the simulation of the system and the simulation of the sensitivities, and the toolbox PESTO (Stapor et al., 2018b) for the estimation of the parameters. All calculations were performed on a server with 2 AMD Opteron 6234 processors, each 2.4 GHz, and 96GB memory.

5 Models and experimental data

In the following, we provide the details of the mathematical models. The considered models vary in their number of parameters (Figure 6A), number of data points that are used to calibrate the models (Figure S1A), and number of states of the underlying ODE system (Figure S1B).

5.1 JAK-STAT signaling I

For the first model, we used the model introduced by Schelker et al. (2012), which is defined by the ODE system

$$\begin{aligned}
\frac{\partial[\mathrm{STAT}]}{\partial t} &= \frac{1}{\Omega_{\mathrm{cyt}}}(\Omega_{\mathrm{nuc}}\,[\mathrm{nSTAT5}]\,p_4 - \Omega_{\mathrm{cyt}}\,[\mathrm{STAT}]\,p_1\,g)\\
\frac{\partial[\mathrm{pSTAT}]}{\partial t} &= -2\,p_2\,[\mathrm{pSTAT}]^2 - [\mathrm{STAT}]\,p_1\,g\\
\frac{\partial[\mathrm{pSTAT_pSTAT}]}{\partial t} &= p_2\,[\mathrm{pSTAT}]^2 - p_3\,[\mathrm{pSTAT_pSTAT}]\\
\frac{\partial[\mathrm{nSTAT1}]}{\partial t} &= -\frac{p_4}{\Omega_{\mathrm{nuc}}}(\Omega_{\mathrm{cyt}}\,[\mathrm{STAT}] - \Omega_{\mathrm{cyt}}\,[\mathrm{STAT}]_0 + 2\,\Omega_{\mathrm{nuc}}\,[\mathrm{nSTAT1}]\\
&\quad + \Omega_{\mathrm{nuc}}\,[\mathrm{nSTAT2}] + \Omega_{\mathrm{nuc}}\,[\mathrm{nSTAT3}] + \Omega_{\mathrm{nuc}}\,[\mathrm{nSTAT4}]\\
&\quad + \Omega_{\mathrm{nuc}}\,[\mathrm{nSTAT5}] + \Omega_{\mathrm{cyt}}\,[\mathrm{pSTAT}] + 2\,\Omega_{\mathrm{cyt}}\,[\mathrm{pSTAT_pSTAT}])\\
\frac{\partial[\mathrm{nSTAT2}]}{\partial t} &= p_4\,([\mathrm{nSTAT1}] - [\mathrm{nSTAT2}])\\
\frac{\partial[\mathrm{nSTAT3}]}{\partial t} &= p_4\,([\mathrm{nSTAT2}] - [\mathrm{nSTAT3}])\\
\frac{\partial[\mathrm{nSTAT4}]}{\partial t} &= p_4\,([\mathrm{nSTAT3}] - [\mathrm{nSTAT4}])\\
\frac{\partial[\mathrm{nSTAT5}]}{\partial t} &= p_4\,([\mathrm{nSTAT4}] - [\mathrm{nSTAT5}]),
\end{aligned}$$

with kinetic parameters $p_1, ..., p_4$. The brackets indicate the concentrations of the corresponding species. The initial conditions are given by

$$\mathbf{x}(t_0) = (1, [\mathrm{pSTAT}]_0, [\mathrm{pSTAT\_pSTAT}]_0, [\mathrm{nSTAT1}]_0, [\mathrm{nSTAT2}]_0, [\mathrm{nSTAT3}]_0, [\mathrm{nSTAT4}]_0, [\mathrm{nSTAT5}]_0)^T,$$

for which the initial condition for STAT is set to 1 in order to remove structural non-identifiabilities (Schelker et al., 2012). The states nSTAT1, . . . , nSTAT5 are intermediate steps, resulting from a linear chain approximation to model the delay of STAT binding to the DNA in the nucleus. The volumes of the cytoplasm and nucleus are denoted by $\Omega_{\mathrm{cyt}} = 1.4\,\mathrm{pl}$ and $\Omega_{\mathrm{nuc}} = 0.45\,\mathrm{pl}$, respectively (Raue et al., 2009).

The observables are defined by y_1 for total concentration of phosphorylated STAT in the cytoplasm (pSTAT), y_2 for the total concentration of STAT in the cytoplasm (tSTAT), and y_3 for the phosphorylated Epo receptors

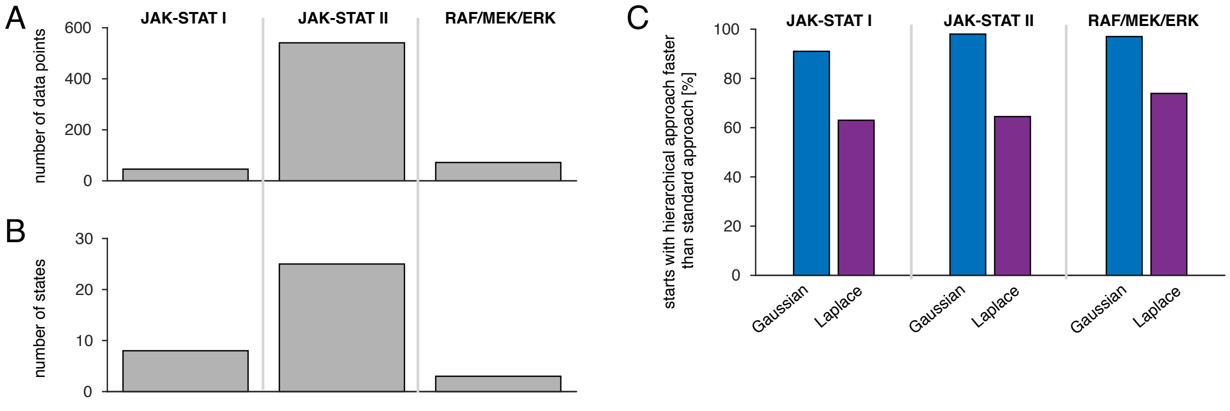

Supplementary Figure S1: Comparison of the models and optimization approaches. (A) Number of experimental data points used to calibrate the models. (B) Number of states n_x. (C) Percentage of starts for which the hierarchical approach was faster than the standard approach.

(pEpoR) (see Figure 3A in the main manuscript). They are linked to the states of the system via

$$\begin{aligned} y_1 &= s_1\left(o_1 + [\text{pSTAT}] + 2[\text{pSTAT\_pSTAT}]\right) \\ y_2 &= s_2\left(o_2 + [\text{STAT}] + [\text{pSTAT}] + 2[\text{pSTAT\_pSTAT}]\right) \\ y_3 &= g\,. \end{aligned}$$

The concentration of Epo receptors is modeled as time-dependent cubic spline function g with parameters $sp_1, \ldots, sp_5$, which are also estimated from the data. The parameters o_1 and o_2 define the offsets needed to model the background noise. The model comprises the parameters $\mathbf{q} = (p_1, p_2, p_3, p_4, sp_1, sp_2, sp_3, sp_4, sp_5, o_1, o_2, s_1, s_2, \sigma_1, \sigma_2, \sigma_3)^T$, for which $\boldsymbol{\theta} = (p_1, p_2, p_3, p_4, sp_1, sp_2, sp_3, sp_4, sp_5, o_1, o_2)$ was optimized in the outer optimization problem of the hierarchical approach. The scaling parameters $\mathbf{s} = (s_1, s_2)$ and noise parameters $\boldsymbol{\sigma} = (\sigma_1, \sigma_2, \sigma_3)$ for observables y_1, y_2, and y_3, respectively, were optimized in the inner optimization problem. The subscript for these parameters indicates the observable. We neglected indices r, e, and c_e, since only one experiment, replicate, and condition is considered. The parameter boundaries for the optimization are given by

$$\log_{10}(\mathbf{q})_{\text{lb}} = (-5, -3, -5, -3, -5, -5, -5, -5, -6, -5, -5, -5, -5, -5, -5, -5)^T$$

for the lower bound and

$$\log_{10}(\mathbf{q})_{\text{ub}} = (3, 6, 3, 6, 3, 3, 3, 3, 3, 3, 3, 3, 3, 3, 3, 3)^T\,.$$

for the upper bound (Maier et al., 2017). We performed 100 optimizations, starting from randomly drawn parameter values. The starting points for the dynamic parameters were the same for both optimization approaches. For a high fraction of the starts, the hierarchical approach was faster than the standard approach (Figure S1C).

The trajectories of the optimizer for the dynamic parameters differ between the standard and the hierarchical approach (Figure S2). For the example shown in Figure S2, the hierarchical approach needs less than half of the steps as the standard approach. Both approaches, however, yield the same parameter values. This is by

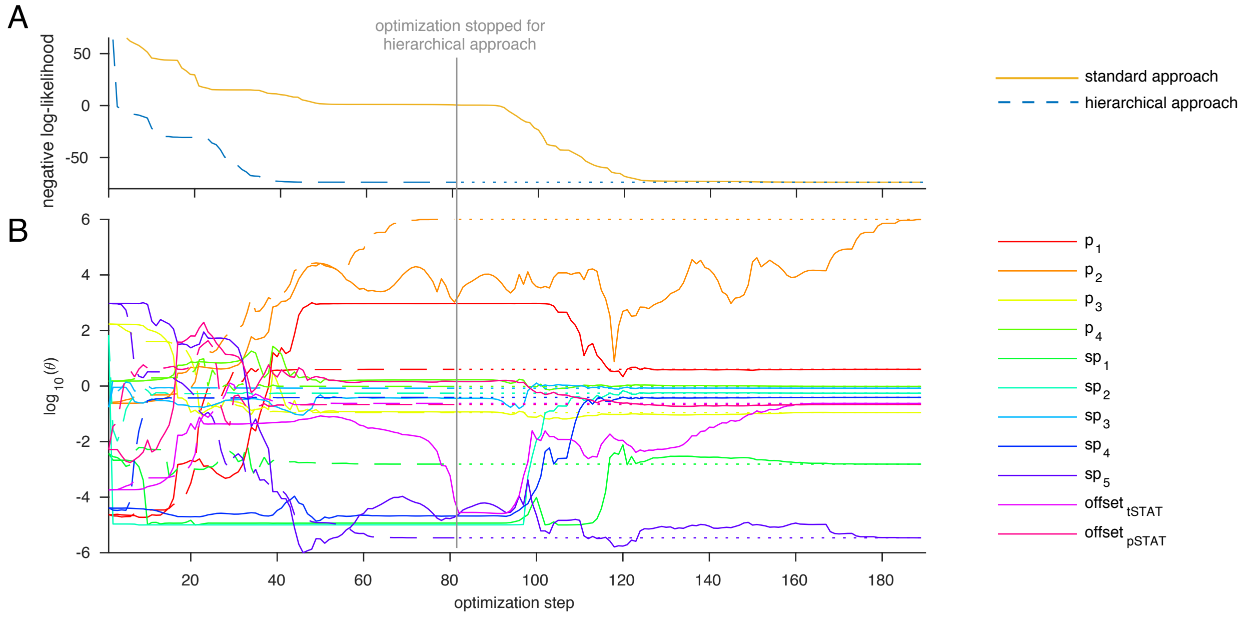

Supplementary Figure S2: Optimization paths for JAK-STAT signaling I. (A) Objective function values and (B) parameter values at each optimization step.

chance true for these runs, but more generally also for multi-start optimization and other global optimization methods.

To evaluate the possibility of using the hierarchical optimization also within global optimization, we repeated the analysis using an particle swarm algorithm (Vaz and Vicente, 2009), which does not need gradient information and thus might be more appropriate in case of the non-differentiability of Laplace noise. The waterfall plots are shown in Figure S3. Interestingly, only the hierarchical optimization for the Gaussian noise was able to find the same optimum as the deterministic optimization. For the other settings the convergence suffered. However, as for the optimization with `fmincon`, the hierarchical approach was superior to the standard approach and the Laplace noise fitted the data better than the Gaussian noise.

We evaluated the uncertainty of the parameters using profile likelihoods (Raue et al., 2009). Employing the standard and the hierarchical approach within the routine for the profile likelihood calculation showed that the profiles coincide for the Gaussian distribution (Figure S4A). While optimization worked well using the Laplace noise, the profile calculation for the Laplace noise for the models considered here showed difficulties, especially when using the standard approach (Figure S4B). Profile calculation for the Laplace noise with the standard method failed to determine the true profile. The profile dropped too early (as visible from the comparison of standard and hierarchical approach) and therefore underestimated the uncertainty. This demonstrates the relevance of the hierarchical approach. In Table S1 we report the maximum likelihood estimates and confidence intervals for both approaches. Further analysis and method development is required to enable a robust profile calculation with Laplace noise, however, employing the hierarchical approach for optimization is already an substantial improvement.

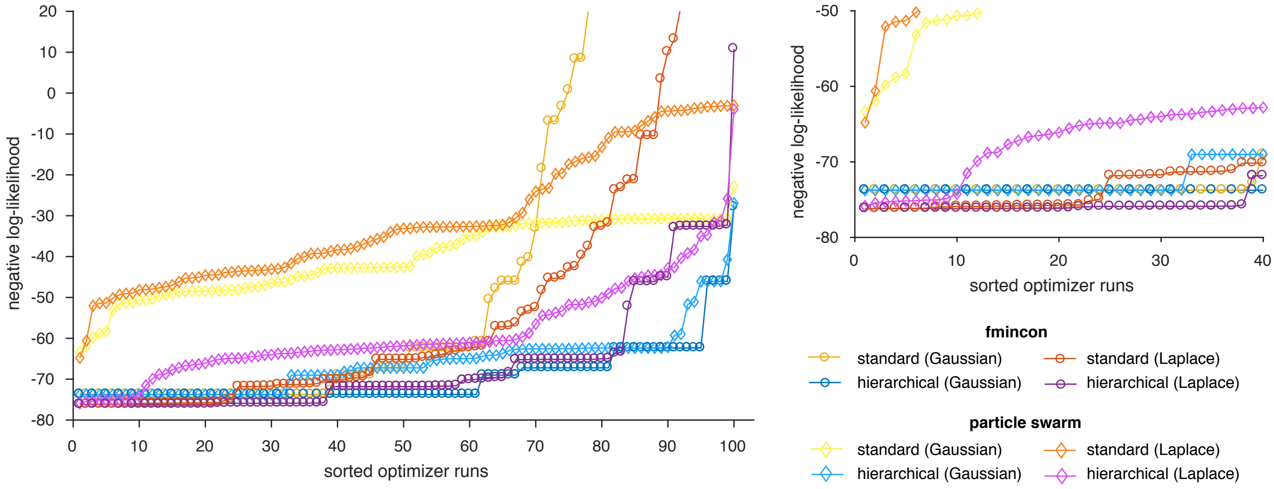

Supplementary Figure S3: Likelihood waterfall plot for JAK-STAT signaling I using particle swarm optimization.

Supplementary Table S1: **Optimization results for JAK-STAT signaling I.** The maximum likelihood estimates (MLE) and 95% confidence intervals (CI) are provided for the $\log_{10}$-parameter values for the standard (st.) and the hierarchical (hier.) approach.

parameter	Gaussian				Laplace			
	MLE $[\log_{10}]$		CI $[\log_{10}]$		MLE $[\log_{10}]$		CI $[\log_{10}]$	
	st.	hier.	st.	hier.	st.	hier.	st.	hier.
p_1	0.603	0.603	[0.327,0.906]	[0.327,0.908]	0.6	0.601	[0.526,0.65]	[0.517,0.675]
p_2	6	6	[-0.0335,>6]	[-0.04,>6]	4.33	5.06	[0.962,>6]	[1.1,>6]
p_3	-0.955	-0.955	[-1.13,-0.761]	[-1.13,-0.761]	-0.977	-0.978	[-1.06,-0.876]	[-1.07,-0.882]
p_4	-0.0111	-0.0111	[-0.107,0.136]	[-0.108,0.137]	0.027	0.0261	[-0.0718,0.078]	[-0.0653,0.0799]
sp_1	-2.81	-2.81	[-3.99,-2.02]	[-3.98,-2.02]	-2.64	-2.64	[-2.81,-2.49]	[-3.65,-2.28]
sp_2	-0.256	-0.256	[-0.355,-0.175]	[-0.355,-0.175]	-0.265	-0.266	[-0.32,-0.225]	[-0.325,-0.21]
sp_3	-0.0765	-0.0765	[-0.122,-0.0369]	[-0.122,-0.0373]	-0.0652	-0.0652	[-0.0842,-0.0402]	[-0.102,-0.0358]
sp_4	-0.407	-0.407	[-0.508,-0.322]	[-0.509,-0.322]	-0.422	-0.419	[-0.505,-0.391]	[-0.507,-0.36]
sp_5	-5.46	-5.46	[<-6,-2.23]	[<-6,-2.23]	-4.37	-4.49	[<-6,-2.69]	[<-6,-2.61]
$\text{offset}_{\text{tSTAT}}$	-0.623	-0.623	[<-5,-0.203]	[<-5,-0.2]	-1.11	-1.11	[<-5,-0.545]	[<-5,-0.518]
$\text{offset}_{\text{pSTAT}}$	-0.664	-0.664	[-0.93,-0.514]	[-0.937,-0.512]	-0.657	-0.656	[-0.778,-0.522]	[-0.74,-0.511]

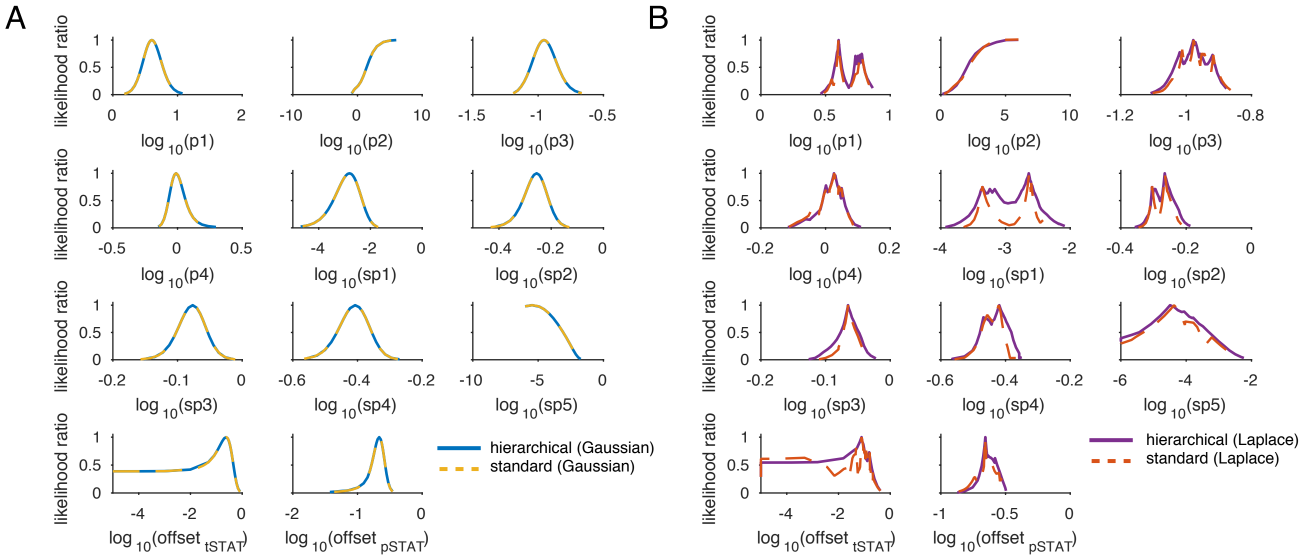

Supplementary Figure S4: Profile likelihoods for JAK-STAT signaling I for (A) Gaussian and (B) Laplace noise.

5.2 JAK-STAT signaling II

The ODE system for JAK-STAT signaling model II is given by (Bachmann et al., 2011)

$$\begin{aligned}
\frac{\partial[\text{EpoRJAK2}]}{\partial t} =& \; [\text{EpoRpJAK2}]\frac{\text{JAK2EpoRDeaSHP1}}{\text{init}_{\text{SHP1}}}[\text{SHP1Act}] \\
&+ \frac{\text{JAK2EpoRDeaSHP1}}{\text{init}_{\text{SHP1}}}[\text{SHP1Act}]\left([\text{p12EpoRpJAK2}] + [\text{p1EpoRpJAK2}] + [\text{p2EpoRpJAK2}]\right) \\
&- \frac{[\text{Epo}]\cdot[\text{EpoRJAK2}]\cdot\text{JAK2ActEpo}}{[\text{SOCS3}]\cdot\frac{\text{SOCS3Inh}}{\text{SOCS3Eqc}} + 1} \\
\frac{\partial[\text{EpoRpJAK2}]}{\partial t} =& \; \frac{[\text{Epo}]\,[\text{EpoRJAK2}]\,\text{JAK2ActEpo}}{[\text{SOCS3}]\frac{\text{SOCS3Inh}}{\text{SOCS3Eqc}} + 1} - \frac{[\text{EpoRpJAK2}]\text{EpoRActJAK2}}{[\text{SOCS3}]\frac{\text{SOCS3Inh}}{\text{SOCS3Eqc}} + 1} \\
&- \frac{3\cdot[\text{EpoRpJAK2}]\cdot\text{EpoRActJAK2}}{(\text{EpoRCISInh}\cdot[\text{EpoRJAK2}_{\text{CIS}}] + 1)\cdot([\text{SOCS3}]\frac{\text{SOCS3Inh}}{\text{SOCS3Eqc}} + 1)} \\
&- [\text{EpoRpJAK2}]\frac{\text{JAK2EpoRDeaSHP1}}{\text{init}_{\text{SHP1}}}[\text{SHP1Act}] \\
\frac{\partial[\text{p1EpoRpJAK2}]}{\partial t} =& \; \frac{[\text{EpoRpJAK2}]\cdot\text{EpoRActJAK2}}{[\text{SOCS3}]\frac{\text{SOCS3Inh}}{\text{SOCS3Eqc}} + 1} - \frac{\text{JAK2EpoRDeaSHP1}}{\text{init}_{\text{SHP1}}}[\text{SHP1Act}][\text{p1EpoRpJAK2}] \\
&- \frac{3\cdot\text{EpoRActJAK2}\cdot[\text{p1EpoRpJAK2}]}{(\text{EpoRCISInh}\cdot[\text{EpoRJAK2}_{\text{CIS}}] + 1)\cdot([\text{SOCS3}]\frac{\text{SOCS3Inh}}{\text{SOCS3Eqc}} + 1)} \\
\frac{\partial[\text{p2EpoRpJAK2}]}{\partial t} =& \; \frac{3\cdot[\text{EpoRpJAK2}]\cdot\text{EpoRActJAK2}}{(\text{EpoRCISInh}\cdot[\text{EpoRJAK2}_{\text{CIS}}] + 1)\cdot([\text{SOCS3}]\frac{\text{SOCS3Inh}}{\text{SOCS3Eqc}} + 1)} \\
&- \frac{\text{EpoRActJAK2}\cdot[\text{p2EpoRpJAK2}]}{[\text{SOCS3}]\frac{\text{SOCS3Inh}}{\text{SOCS3Eqc}} + 1} - \frac{\text{JAK2EpoRDeaSHP1}}{\text{init}_{\text{SHP1}}}[\text{SHP1Act}][\text{p2EpoRpJAK2}] \\
\frac{\partial[\text{p12EpoRpJAK2}]}{\partial t} =& \; \frac{\text{EpoRActJAK2}\cdot[\text{p2EpoRpJAK2}]}{[\text{SOCS3}]\frac{\text{SOCS3Inh}}{\text{SOCS3Eqc}} + 1} - \frac{\text{JAK2EpoRDeaSHP1}}{\text{init}_{\text{SHP1}}}\cdot[\text{SHP1Act}]\cdot[\text{p12EpoRpJAK2}] \\
&+ \frac{3\cdot\text{EpoRActJAK2}\cdot[\text{p1EpoRpJAK2}]}{(\text{EpoRCISInh}\cdot[\text{EpoRJAK2}_{\text{CIS}}] + 1)([\text{SOCS3}]\frac{\text{SOCS3Inh}}{\text{SOCS3Eqc}} + 1)}
\end{aligned}$$

$$
\begin{aligned}
\frac{\partial[\mathrm{EpoRJAK2_{CIS}}]}{\partial t} &= -[\mathrm{EpoRJAK2_{CIS}}]\cdot\frac{\mathrm{EpoRCISRemove}}{\mathrm{init_{EpoRJAK2}}}\,([\mathrm{p12EpoRpJAK2}]+[\mathrm{p1EpoRpJAK2}])\\
\frac{\partial[\mathrm{SHP1}]}{\partial t} &= -\mathrm{SHP1Dea}[\mathrm{SHP1Act}]-[\mathrm{SHP1}]\cdot\frac{\mathrm{SHP1ActEpoR}}{\mathrm{init_{EpoRJAK2}}}\\
&\quad\cdot\big([\mathrm{EpoRpJAK2}]+[\mathrm{p12EpoRpJAK2}]+[\mathrm{p1EpoRpJAK2}]+[\mathrm{p2EpoRpJAK2}]\big)\\
\frac{\partial[\mathrm{SHP1Act}]}{\partial t} &= -[\mathrm{SHP1}]\cdot\frac{\mathrm{SHP1ActEpoR}}{\mathrm{init_{EpoRJAK2}}}\cdot\big([\mathrm{EpoRpJAK2}]\\
&\quad+[\mathrm{p12EpoRpJAK2}]+[\mathrm{p1EpoRpJAK2}]+[\mathrm{p2EpoRpJAK2}]\big)-\mathrm{SHP1Dea}\cdot[\mathrm{SHP1Act}]\\
\frac{\partial[\mathrm{STAT5}]}{\partial t} &= -\frac{\mathrm{STAT5Exp}\cdot[\mathrm{npSTAT5}]\cdot 0.275}{0.4}\\
&\quad-\frac{[\mathrm{STAT5}]\cdot\frac{\mathrm{STAT5ActJAK2}}{\mathrm{init_{EpoRJAK2}}}}{[\mathrm{SOCS3}]\frac{\mathrm{SOCS3Inh}}{\mathrm{SOCS3Eqc}}+1}\\
&\quad\cdot([\mathrm{EpoRpJAK2}+[\mathrm{p12EpoRpJAK2}]+[\mathrm{p1EpoRpJAK2}]+[\mathrm{p2EpoRpJAK2}])\\
&\quad-[\mathrm{STAT5}]\frac{\mathrm{STAT5ActEpoR}}{\mathrm{init^2_{EpoRJAK2}}}\frac{([\mathrm{p12EpoRpJAK2}]+[\mathrm{p1EpoRpJAK2}])^2}{([\mathrm{CIS}]\frac{\mathrm{CISInh}}{\mathrm{CISEqc}}+1)([\mathrm{SOCS3}]\frac{\mathrm{SOCS3Inh}}{\mathrm{SOCS3Eqc}}+1)}\\
\frac{\partial[\mathrm{pSTAT5}]}{\partial t} &= \frac{[\mathrm{STAT5}]\frac{\mathrm{STAT5ActJAK2}}{\mathrm{init_{EpoRJAK2}}}\cdot([\mathrm{EpoRpJAK2}]+[\mathrm{p12EpoRpJAK2}]+[\mathrm{p1EpoRpJAK2}]+[\mathrm{p2EpoRpJAK2}])}{[\mathrm{SOCS3}]\frac{\mathrm{SOCS3Inh}}{\mathrm{SOCS3Eqc}}+1}\\
&\quad-\mathrm{STAT5Imp}\cdot[\mathrm{pSTAT5}]+[\mathrm{STAT5}]\frac{\mathrm{STAT5ActEpoR}}{\mathrm{init^2_{iEpoRJAK2}}}\cdot\frac{([\mathrm{p12EpoRpJAK2}]+[\mathrm{p1EpoRpJAK2}])^2}{([\mathrm{CIS}]\frac{\mathrm{CISInh}}{\mathrm{CISEqc}}+1)\cdot([\mathrm{SOCS3}]\frac{\mathrm{SOCS3Inh}}{\mathrm{SOCS3Eqc}}+1)}\\
\frac{\partial[\mathrm{npSTAT5}]}{\partial t} &= \frac{\mathrm{STAT5Imp}\cdot 0.4\cdot[\mathrm{pSTAT5}]}{0.275}-\mathrm{STAT5Exp}\cdot[\mathrm{npSTAT5}]\\
\frac{\partial[\mathrm{CISnRNA1}]}{\partial t} &= -[\mathrm{CISnRNA1}]\cdot\mathrm{CISRNADelay}-\frac{1}{\mathrm{init_{STAT5}}}\cdot\mathrm{CISRNATurn}\cdot[\mathrm{npSTAT5}]\cdot(\mathrm{ActD}-1)\\
\frac{\partial[\mathrm{CISnRNA2}]}{\partial t} &= [\mathrm{CISnRNA1}]\cdot\mathrm{CISRNADelay}-[\mathrm{CISnRNA2}]\cdot\mathrm{CISRNADelay}\\
\frac{\partial[\mathrm{CISnRNA3}]}{\partial t} &= [\mathrm{CISnRNA2}]\cdot\mathrm{CISRNADelay}-[\mathrm{CISnRNA3}]\cdot\mathrm{CISRNADelay}\\
\frac{\partial[\mathrm{CISnRNA4}]}{\partial t} &= [\mathrm{CISnRNA3}]\cdot\mathrm{CISRNADelay}-[\mathrm{CISnRNA4}]\cdot\mathrm{CISRNADelay}\\
\frac{\partial[\mathrm{CISnRNA5}]}{\partial t} &= [\mathrm{CISnRNA4}]\cdot\mathrm{CISRNADelay}-[\mathrm{CISnRNA5}]\cdot\mathrm{CISRNADelay}\\
\frac{\partial[\mathrm{CISRNA}]}{\partial t} &= \frac{[\mathrm{CISnRNA5}]\cdot\mathrm{CISRNADelay}\cdot 0.275}{0.4}-[\mathrm{CISRNA}]\cdot\mathrm{CISRNATurn}\\
\frac{\partial[\mathrm{CIS}]}{\partial t} &= [\mathrm{CISRNA}]\cdot\mathrm{CISEqc}\cdot\mathrm{CISTurn}-[\mathrm{CIS}]\cdot\mathrm{CISTurn}+\mathrm{CISoe}\cdot\mathrm{CISTurn}\cdot\mathrm{CISEqcOE}\cdot\mathrm{CISEqc}\\
\frac{\partial[\mathrm{SOCS3nRNA1}]}{\partial t} &= -[\mathrm{SOCS3nRNA1}]\cdot\mathrm{SOCS3RNADelay}-\frac{1}{\mathrm{init_{STAT5}}}\cdot\mathrm{SOCS3RNATurn}\cdot[\mathrm{npSTAT5}]\cdot(\mathrm{ActD}-1)\\
\frac{\partial[\mathrm{SOCS3nRNA2}]}{\partial t} &= [\mathrm{SOCS3nRNA1}]\cdot\mathrm{SOCS3RNADelay}-[\mathrm{SOCS3nRNA2}]\cdot\mathrm{SOCS3RNADelay}\\
\frac{\partial[\mathrm{SOCS3nRNA3}]}{\partial t} &= [\mathrm{SOCS3nRNA2}]\cdot\mathrm{SOCS3RNADelay}-[\mathrm{SOCS3nRNA3}]\cdot\mathrm{SOCS3RNADelay}\\
\frac{\partial[\mathrm{SOCS3nRNA4}]}{\partial t} &= [\mathrm{SOCS3nRNA3}]\cdot\mathrm{SOCS3RNADelay}-[\mathrm{SOCS3nRNA4}]\cdot\mathrm{SOCS3RNADelay}\\
\frac{\partial[\mathrm{SOCS3nRNA5}]}{\partial t} &= [\mathrm{SOCS3nRNA4}]\cdot\mathrm{SOCS3RNADelay}-[\mathrm{SOCS3nRNA5}]\cdot\mathrm{SOCS3RNADelay}\\
\frac{\partial[\mathrm{SOCS3RNA}]}{\partial t} &= \frac{[\mathrm{SOCSnRNA5}]\cdot\mathrm{SOCSRNADelay}\cdot 0.275}{0.4}-[\mathrm{SOCSRNA}]\cdot\mathrm{SOCSRNATurn}\\
\frac{\partial[\mathrm{SOCS3}]}{\partial t} &= [\mathrm{SOCS3RNA}]\cdot\mathrm{SOCS3Eqc}\cdot\mathrm{SOCS3Turn}-[\mathrm{SOCS3}]\cdot\mathrm{SOCS3Turn}\\
&\quad+\mathrm{SOCS3oe}\cdot\mathrm{SOCS3Turn}\cdot\mathrm{SOCS3EqcOE}\cdot\mathrm{SOCS3Eqc}\,,
\end{aligned}
$$

with condition-specific initial conditions (see Table S2) denoted by $x_{i,c_e}(0)$ for observable index i under condition indexed by c_e:

$$x_{1,c_e}(0) = \text{init}_{\text{EpoRJAK2}}, x_{9,c_e}(0) = \text{init}_{\text{STAT5}}, x_{i,c_e}(0) = 0, i = \{2,3,4,5,8,10,11,12,13,14,15,16,17,19,20,21,22,23,24\}, \forall c_e$$
$$x_{i,c_e}(0) = 0, i = \{6,18,25\}, c_e = \{1,2,3,4,5,6,15,\dots,36\}$$
$$x_{6,c_e}(0) = u_{c_e,2}, x_{18,c_e}(0) = u_{c_e,2} \cdot (\text{CISEqc} \cdot \text{CISEqcOE}), c_e = \{7,8,9,10\}$$
$$x_{7,c_e}(0) = \text{init}_{\text{SHP1}}, c_e = \{1,2,3,4,5,6,7,8,9,10,11,12,15,\dots,36\}$$
$$x_{7,c_e}(0) = (1 + u_{c_e,4} \cdot \text{SHP1ProOE}) \cdot \text{init}_{\text{SHP1}}, c_e = \{13,14\}$$
$$x_{6,c_e}(0) = 0, x_{18,c_e}(0) = 0, x_{25,c_e}(0) = u_{c_e,3} \cdot (\text{SOCS3Eqc} \cdot \text{SOCS3EqcOE}), c_e = \{11,12\}$$

The observables are given by

$$y_1 = \text{pJAK2}_{\text{au}} = s_{1,c_e} \cdot \Big(o_{1,c_e} + \frac{2}{\text{init}_{\text{EpoRJAK2}}} \cdot ([\text{EpoRpJAK2}] + [\text{p1EpoRpJAK2}] + [\text{p2EpoRpJAK2}] + [\text{p12EpoRpJAK2}])\Big)$$
$$y_2 = \text{pEpoR}_{\text{au}} = s_{2,c_e} \cdot \left(o_{2,c_e} + \frac{16}{\text{init}_{\text{EpoRJAK2}}} \cdot ([\text{p1EpoRpJAK2}] + [\text{p2EpoRpJAK2}] + [\text{p12EpoRpJAK2}])\right)$$
$$y_3 = \text{CIS}_{\text{au}} = s_{3,c_e} \cdot \left(o_{3,c_e} + \frac{[\text{CIS}]}{\text{CISEqc}}\right)$$
$$y_4 = \text{SOCS3}_{\text{au}} = s_{4,c_e} \cdot \left(o_{4,c_e} + \frac{[\text{SOCS3}]}{\text{SOCS3Eqc}}\right)$$
$$y_5 = \text{tSTAT5}_{\text{au}} = s_{5,c_e} \cdot \left(\frac{1}{\text{init}_{\text{STAT5}}}([\text{STAT5}] + [\text{pSTAT5}])\right)$$
$$y_6 = \text{pSTAT5}_{\text{au}} = s_{6,c_e} \cdot \left(o_{6,c_e} + \frac{1}{\text{init}_{\text{STAT5}}}[\text{pSTAT5}]\right)$$
$$y_7 = \text{STAT5}_{\text{abs}} = [\text{STAT5}]$$
$$y_8 = \text{SHP1}_{\text{abs}} = [\text{SHP1}] + [\text{SHP1Act}]$$
$$y_9 = \text{CIS}_{\text{abs}} = [\text{CIS}]$$
$$y_{10} = \text{SOCS3}_{\text{abs}} = [\text{SOCS3}]$$
$$y_{11} = \text{pSTAT5B}_{\text{rel}} = o_{11} + 100\frac{[\text{pSTAT5}]}{[\text{pSTAT5}] + [\text{STAT5}]}$$
$$y_{12} = \text{SOCS3RNA}_{\text{foldA}} = 1 + s_{12} \cdot [\text{SOCS3RNA}]$$
$$y_{13} = \text{SOCS3RNA}_{\text{foldB}} = 1 + s_{13} \cdot [\text{SOCS3RNA}]$$
$$y_{14} = \text{SOCS3RNA}_{\text{foldC}} = 1 + s_{14} \cdot [\text{SOCS3RNA}]$$
$$y_{15} = \text{CISRNA}_{\text{foldA}} = 1 + s_{15} \cdot [\text{CISRNA}]$$
$$y_{16} = \text{CISRNA}_{\text{foldB}} = 1 + s_{16} \cdot [\text{CISRNA}]$$
$$y_{17} = \text{CISRNA}_{\text{foldC}} = 1 + s_{17} \cdot [\text{CISRNA}]$$
$$y_{18} = \text{tSHP1}_{\text{au}} = s_{18} \cdot \left(\frac{1}{\text{init}_{\text{SHP1}}}([\text{SHP1}] + [\text{SHP1Act}])(1 + (\text{SHP1oe} \cdot \text{SHP1ProOE}))\right)$$
$$y_{19} = \text{CIS}_{\text{au1}} = s_{19} \cdot \frac{[\text{CIS}]}{\text{CISEqc}}$$
$$y_{20} = \text{CIS}_{\text{au2}} = s_{20} \cdot \frac{[\text{CIS}]}{\text{CISEqc}}\,.$$

The parameters $\boldsymbol{\theta}$ are

$$\boldsymbol{\theta} = (\text{CISEqc}, \text{CISEqcOE}, \text{CISInh}, \text{CISRNADelay}, \text{CISRNATurn}, \text{CISTurn}, \text{EpoRActJAK2}, \text{EpoRCISInh}, \text{EpoRCISRemove}, \text{JAK2ActEpo}, \text{JAK2EpoRDeaSHP1}, \text{SHP1ActEpoR}, \text{SHP1Dea}, \text{SHP1ProOE}, \text{SOCS3Eqc}, \text{SOCS3EqcOE}, \text{SOCS3Inh}, \text{SOCS3RNADelay}, \text{SOCS3RNATurn}, \text{SOCS3Turn}, \text{STAT5ActEpoR}, \text{STAT5ActJAK2}, \text{STAT5Exp}, \text{STAT5Imp}, \text{init}_{\text{EpoRJAK2}}, \text{init}_{\text{SHP1}}, \text{init}_{\text{STAT5}}\ o_{1,1}, o_{1,4}, o_{1,6}, o_{1,7}, o_{1,11}, o_{1,13}, o_{1,15}, o_{1,20}, o_{2,1}, o_{2,4}, o_{2,6}, o_{2,7}, o_{2,9}, o_{2,11}, o_{2,13}, o_{2,15}, o_{2,20}, o_{3,1}, o_{3,4}, o_{3,7}, o_{3,11}, o_{3,13}, o_{4,1}, o_{4,7}, o_{4,11}, o_{6,1}, o_{6,2}, o_{6,4}, o_{6,7}, o_{6,11}, o_{6,13})^T$$

with $n_\theta = 58$. For experiment SHP1oe ($e = 9$), the parameter $\text{init}_{\text{SHP1}}$ was replaced by $\text{init}_{\text{SHP1}} \cdot (1 + (\text{SHP1oe} \cdot \text{SHP1ProOE}))$ in the model equations. For the notation of the offset, scaling, and noise parameters, we neglected the index r, since these parameters are shared for the replicates. The first subscript indicates the observable, and the second the condition. However, all conditions belonging to the same experiment share the scaling and offset parameters and thus the parameters are only listed for the first condition of each experiment. The experiments and corresponding condition indices are summarized in Table S2. For simplicity, we note the scaling parameters as vector $\mathbf{s}$ which contains only the unique parameters s_{i,c_e} which need to be estimated from the data. Thus, it is

$$\mathbf{s} = (s_{1,1}, s_{1,4}, s_{1,6}, s_{1,7}, s_{1,11}, s_{1,15}, s_{1,20}, s_{2,1}, s_{2,4}, s_{2,5}, s_{2,7}, s_{2,9}, s_{2,11}, s_{2,13}, s_{2,15}, s_{2,20}, s_{3,1}, s_{3,4}, s_{3,7}, s_{3,11}, s_{3,13}, s_{4,1}, s_{4,7}, s_{4,11}, s_{5,1}, s_{5,4}, s_{5,13}, s_{6,1}, s_{6,4}, s_{6,7}, s_{6,11}, s_{6,13}, s_{6,26}, s_{12}, s_{13}, s_{14}, s_{15}, s_{16}, s_{17}, s_{18}, s_{19}, s_{20})^T$$

with $n_s = 42$. The noise parameters do not differ between experiments or replicates, thus, neglecting the subscripts for the experiment-specific condition index c_e and for the replicate index r, the noise parameters, which need to be estimated from the data are given by

$$\boldsymbol{\sigma} = (\sigma_1, \sigma_3, \sigma_4, \sigma_5, \sigma_7, \sigma_8, \sigma_9, \sigma_{10}, \sigma_{11}, \sigma_{12}, \sigma_{18})^T$$

with $n_\sigma = 11$. Some observables have the same noise parameters:

$$\sigma_1 = \sigma_2$$
$$\sigma_3 = \sigma_{19} = \sigma_{20}\,,$$
$$\sigma_5 = \sigma_6\,,$$
$$\sigma_{12} = \sigma_{13} = \sigma_{14} = \sigma_{15} = \sigma_{16} = \sigma_{17}\,.$$

A minor modification from the model proposed by Bachmann et al. (2011) is that the parameterization for the noise of $\text{pSTAT5B}_{\text{au}}$ does not include an additional parameter for the SOCS3oe experiment, and that the observables for RNA were fitted in linear space. The observable $\text{pSTAT5B}_{\text{rel}}$ was also fitted on a linear scale, while the other observables were compared at a $\log_{10}$ scale (as done by Bachmann et al. (2011)). In our setting, the offset parameters were also multiplied with the scaling parameters, which yielded different optimal values for the offset parameters compared to those found by Bachmann et al. (2011). We performed 100 multi-starts for Gaussian noise and 200 for Laplace noise for both optimization approaches. The parameter boundaries are $\log_{10}(\boldsymbol{\theta})_{\text{lb}} = -3$ and $\log_{10}(\boldsymbol{\theta})_{\text{ub}} = 3$, except for

$$\log_{10}(\text{CISEqc}, \text{CISInh}, \text{EpoRActJAK2}, \text{EpoRCISInh}, \text{JAK2ActEpo}, \text{JAK2EpoRDeaSHP1}, \text{SOCS3Turn})_{\text{ub}} = (4, 12, 5, 6, 9, 4, 4)^T$$
$$\log_{10}(o_{i,c_e})_{\text{lb}} = -5, \quad \log_{10}(o_{i,c_e})_{\text{ub}} = 3 \quad \forall i, c_e$$
$$\log_{10}(\mathbf{s})_{\text{lb}} = (-3, \ldots, -3)^T, \quad \log_{10}(\mathbf{s})_{\text{ub}} = (3, \ldots, 3)^T$$
$$\log_{10}(\boldsymbol{\sigma})_{\text{lb}} = (-3, \ldots, -3)^T, \quad \log_{10}(\boldsymbol{\sigma})_{\text{ub}} = (3, \ldots, 3)^T\,.$$

Supplementary Table S2: Overview for the experimental data of JAK-STAT signaling model II.

name	experiment index e	condition index	condition $\mathbf{u}$				
			ActD	CISoe	SOCS3oe	SHP1oe	$[\mathrm{Epo}]/10^{-6}$
Long	1	1	0	0	0	0	0.125
Concentration	2	2	0	0	0	0	0.125
RNA	3	3	0	0	0	0	0.125
ActD	4	4	0	0	0	0	0.125
		5	1	0	0	0	0.125
Fine	5	6	0	0	0	0	1.25
CISoe	6	7	0	0	0	0	0.125
		8	0	1	0	0	0.125
CISoe_pEpoR	7	9	0	0	0	0	0.125
		10	0	1	0	0	0.125
SOCS3oe	8	11	0	0	0	0	0.125
		12	0	0	1	0	0.125
SHP1oe	9	13	0	0	0	0	0.125
		14	0	0	0	1	0.125
dose response 7 min	10	15	0	0	0	0	0.0025
		16	0	0	0	0	0.025
		17	0	0	0	0	0.25
		18	0	0	0	0	2.5
		19	0	0	0	0	25
dose response 30 min	11	20	0	0	0	0	0.0025
		21	0	0	0	0	0.025
		22	0	0	0	0	0.125
		23	0	0	0	0	0.25
		24	0	0	0	0	1.25
		25	0	0	0	0	2.5
dose response 10 min	12	26	0	0	0	0	0.0025
		27	0	0	0	0	0.0125
		28	0	0	0	0	0.025
		29	0	0	0	0	0.125
		30	0	0	0	0	0.25
		31	0	0	0	0	2.5
dose response 90 min	13	32	0	0	0	0	0.0025
		33	0	0	0	0	0.1025
		34	0	0	0	0	0.125
		35	0	0	0	0	0.25
		36	0	0	0	0	2.5

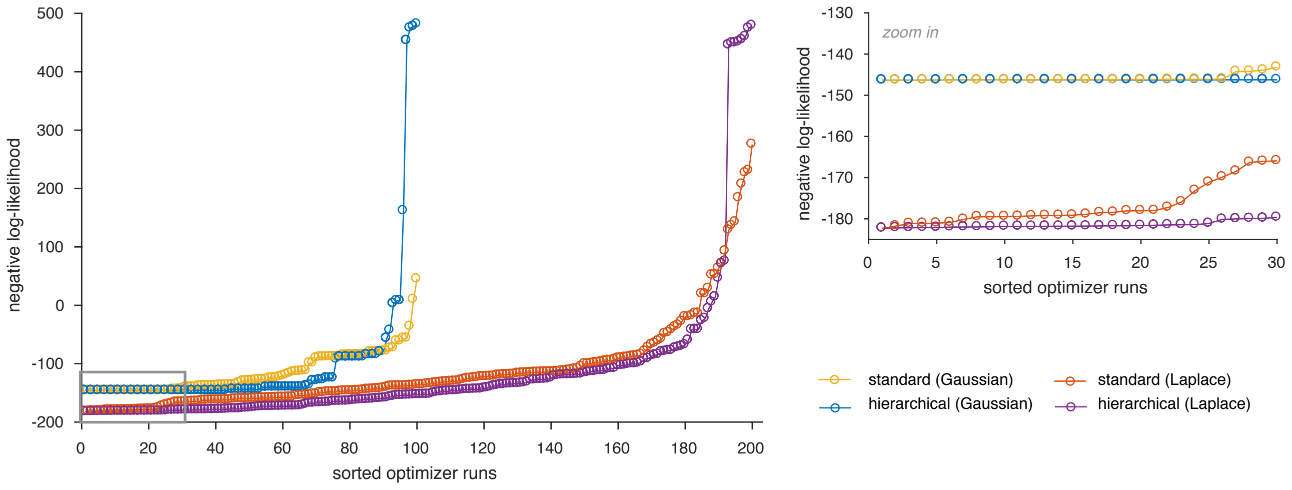

Supplementary Figure S5: Likelihood waterfall for the JAK-STAT signaling model II.

The fitted experimental data for the whole data set are shown in Figure S6.

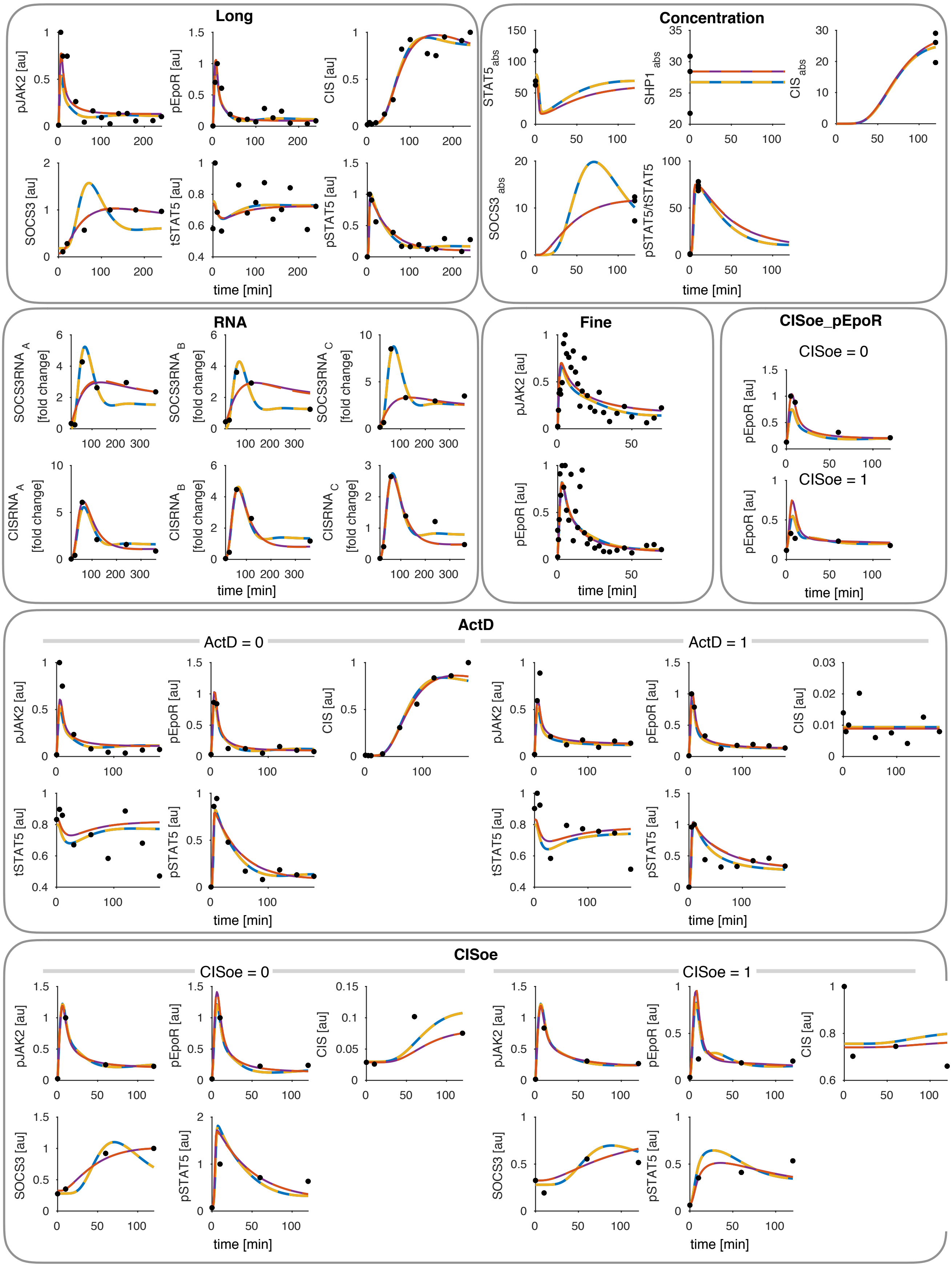
Long
Concentration
RNA
Fine
CISoe_pEpoR
CISoe = 0
CISoe = 1
ActD
ActD = 0
ActD = 1
CISoe
CISoe = 0
CISoe = 1
pJAK2 [au]
pEpoR [au]
CIS [au]
SOCS3 [au]
tSTAT5 [au]
pSTAT5 [au]
STAT5abs
SHP1abs
CISabs
SOCS3abs
pSTAT5/tSTAT5
SOCS3RNA A [fold change]
SOCS3RNA B [fold change]
SOCS3RNA C [fold change]
CISRNA A [fold change]
CISRNA B [fold change]
CISRNA C [fold change]
time [min]

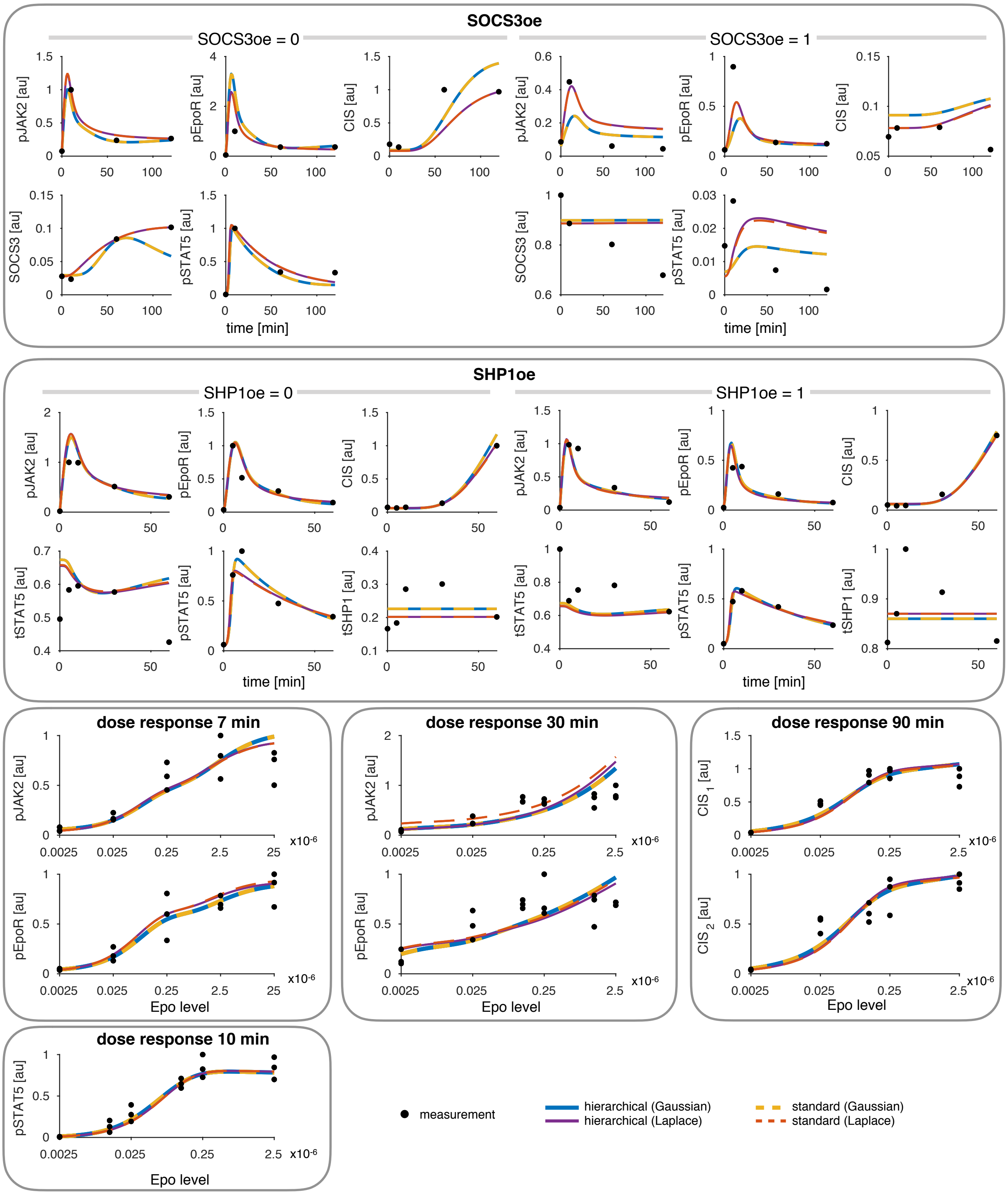

Supplementary Figure S6: Experimental data for JAK-STAT signaling model II. Boxes indicate different experiments. The lines highlight the different models (Gaussian and Laplace noise) and optimization approaches (standard and hierarchical).

Supplementary Table S3: **Optimization results for JAK-STAT signaling II.** The maximum likelihood estimates (MLE) are provided for the $\log_{10}$-parameter values.

parameter	Gaussian		Laplace	
	MLE [$\log_{10}$]		MLE [$\log_{10}$]	
	standard	hierarchical	standard	hierarchical
CISEqc	2.78	2.79	2.94	2.93
CISEqcOE	-0.423	-0.424	-0.338	-0.33
CISInh	8.69	8.69	10.3	10.2
CISRNADelay	-0.861	-0.861	-0.775	-0.785
CISRNATurn	2.74	2.69	-0.496	-0.418
CISTurn	-2.16	-2.16	-2.27	-2.27
EpoRActJAK2	-0.483	-0.483	-0.445	-0.489
EpoRCISInh	5.87	5.87	3.23	4.3
EpoRCISRemove	0.739	0.739	0.252	0.399
JAK2ActEpo	5.86	5.86	5.96	5.97
JAK2EpoRDeaSHP1	2.17	2.17	1.79	1.89
SHP1ActEpoR	-3	-3	-2.68	-2.79
SHP1Dea	-2.13	-2.13	-2.26	-2.23
SHP1ProOE	0.447	0.447	0.52	0.52
SOCS3Eqc	2.24	2.24	2.59	2.6
SOCS3EqcOE	0.248	0.248	-0.464	-0.475
SOCS3Inh	0.911	0.912	1.33	1.32
SOCS3RNADelay	-0.774	-0.774	0.744	1.24
SOCS3RNATurn	-1.02	-1.03	-2.33	-2.35
SOCS3Turn	2.93	2.96	1.22	1.2
STAT5ActEpoR	1.31	1.31	1.43	1.44
STAT5ActJAK2	-1.07	-1.07	-1.29	-1.3
STAT5Exp	-1	-1	-1.02	-1.02
STAT5Imp	-1.51	-1.51	-1.65	-1.64
$\text{init}_{\text{EpoRJAK2}}$	0.0868	-0.00905	0.00109	0.00854
$\text{init}_{\text{SHP1}}$	1.43	1.43	1.45	1.45
$\text{init}_{\text{STAT5}}$	1.9	1.9	1.84	1.84
$o_{3,4}$	-3.33	-3.33	-3.48	-3.47
$o_{3,7}$	-1.81	-1.81	-1.73	-1.72
$o_{3,1}$	-2.93	-2.93	-2.92	-2.91
$o_{3,13}$	-3.09	-3.09	-3.14	-3.14
$o_{3,11}$	-2.55	-2.55	-2.58	-2.57
$o_{4,7}$	-1.41	-1.41	-1.85	-1.86
$o_{4,1}$	-1.83	-1.83	-2.62	-2.66
$o_{4,11}$	-1.22	-1.22	-1.95	-1.96
$o_{2,4}$	-1.03	-1.03	-0.955	-0.993
$o_{2,7}$	-0.947	-0.947	-0.916	-0.911
$o_{2,9}$	-0.011	-0.0109	-0.126	-0.126
$o_{2,20}$	-3.6	-3.66	-0.672	-0.753
$o_{2,15}$	-0.557	-0.557	-0.662	-0.679
$o_{2,6}$	-0.02	-0.0199	-0.322	-0.307
$o_{2,1}$	-1.73	-1.73	-1.67	-1.67
$o_{2,13}$	-0.857	-0.856	-0.742	-0.741
$o_{2,11}$	-1.05	-1.05	-0.88	-0.876
$o_{1,4}$	-1.66	-1.66	-1.63	-1.62
$o_{1,7}$	-1.93	-1.93	-1.77	-1.75
$o_{1,20}$	-1.73	-1.73	-1.1	-3.12
$o_{1,15}$	-1.05	-1.05	-1.19	-1.18
$o_{1,6}$	-1.26	-1.26	-1.28	-1.27
$o_{1,1}$	-1.95	-1.95	-1.98	-1.96
$o_{1,13}$	-1.93	-1.93	-2.02	-2.01
o_{11}	-1.39	-1.39	-1.32	-1.31
$o_{6,4}$	-2.81	-2.81	-2.77	-2.77
$o_{6,7}$	-1.55	-1.55	-1.51	-1.52
$o_{6,2}$	-0.312	-0.311	-0.165	-0.165
$o_{6,1}$	-3.13	-3.13	-3.09	-3.09
$o_{6,13}$	-1.36	-1.36	-1.31	-1.32
$o_{6,11}$	-2.31	-2.31	-2.43	-2.43

5.3 RAF/MEK/ERK signaling

The ODE system for the RAF/MEK/ERK signaling model is given by

$$\begin{aligned}\frac{dx_1}{\partial t} &= k_{1,\max}(t)\frac{K_1}{K_1+[\text{pERK}]}(1-x_1)-k_2x_1\\ \frac{dx_2}{\partial t} &= \frac{k_3[\text{Raf}]_0K_2x_1}{K_2+[\text{sora}]}(1-x_2)-k_4x_2\\ \frac{dx_3}{\partial t} &= \frac{k_5[\text{MEK}]_0K_3x_2}{K_3+[\text{UO126}]}(1-x_3)-k_6x_3\end{aligned}$$

with states $x_1 = [\text{pRaf}]/[\text{Raf}]_0$, $x_2 = [\text{pMEK}]/[\text{MEK}]_0$, and $x_3 = [\text{pERK}]/[\text{ERK}]_0$, and

$$k_{1,\max}(t) = k_{1,0} + k_{1,1}\left(1-\exp\left(-\frac{t}{\tau_1}\right)\right)\exp\left(-\frac{t}{\tau_2}\right)$$

(see (Fiedler et al., 2016) for more details). The initial conditions were assumed to be the steady states reached without stimulation and for $k_{1,\max} = k_{1,0}$. Defining $\tilde{K}_1 = K_1/[\text{ERK}]_0$, $\tilde{k}_3 = k_3[\text{Raf}]_0$ and $\tilde{k}_5 = k_5[\text{MEK}]_0$, we obtain

$$\begin{aligned}x_1(0) = \Bigg(&\tilde{K}_1k_{1,0} + \Bigg(\tilde{K}_1^2k_{1,0}^2 + \frac{2\tilde{K}_1^2k_6k_{1,0}^2}{\tilde{k}_5} + \frac{\tilde{K}_1^2k_6^2k_{1,0}^2}{\tilde{k}_5^2} + \frac{\tilde{K}_1^2k_4^2k_6^2\left(k_{1,0}+k_2\right)^2}{(\tilde{k}_3\tilde{k}_5)^2} +\\ &\frac{2\tilde{K}_1^2k_4k_6^2k_{1,0}\left(k_{1,0}+k_2\right)}{\tilde{k}_3\tilde{k}_5^2} + \frac{2\tilde{K}_1^2k_4k_6k_{1,0}\left(k_{1,0}+k_2\right)}{\tilde{k}_3\tilde{k}_5} + \frac{4\tilde{K}_1k_2k_4k_6k_{1,0}}{\tilde{k}_3\tilde{k}_5}\Bigg)^{\frac{1}{2}} + \frac{\tilde{K}_1k_6k_{1,0}}{\tilde{k}_5} -\\ &\frac{\tilde{K}_1k_4k_6\left(k_{1,0}+k_2\right)}{\tilde{k}_3\tilde{k}_5}\Bigg) \Bigg/ \left(2\left(k_2+\tilde{K}_1k_{1,0}+\tilde{K}_1k_2+\frac{\tilde{K}_1k_2k_6}{\tilde{k}_5}+\frac{\tilde{K}_1k_6k_{1,0}}{\tilde{k}_5}\right)\right)\end{aligned}$$

$$\begin{aligned}x_2(0) = \Bigg(\Bigg(&\tilde{K}_1^2k_{1,0}^2 + \frac{2\tilde{K}_1^2k_6k_{1,0}^2}{\tilde{k}_5} + \frac{\tilde{K}_1^2k_6^2k_{1,0}^2}{\tilde{k}_5^2} + \frac{\tilde{K}_1^2k_4^2k_6^2\left(k_{1,0}+k_2\right)^2}{(\tilde{k}_3\tilde{k}_5)^2} +\\ &\frac{2\tilde{K}_1^2k_4k_6^2k_{1,0}\left(k_{1,0}+k_2\right)}{\tilde{k}_3\tilde{k}_5^2} + \frac{2\tilde{K}_1^2k_4k_6k_{1,0}\left(k_{1,0}+k_2\right)}{\tilde{k}_3\tilde{k}_5} + \frac{4\tilde{K}_1k_2k_4k_6k_{1,0}}{\tilde{k}_3\tilde{k}_5}\Bigg)^{\frac{1}{2}} +\\ &\tilde{K}_1k_{1,0} + \frac{\tilde{K}_1k_6k_{1,0}}{\tilde{k}_5} - \frac{\tilde{K}_1k_2k_4k_6}{\tilde{k}_3\tilde{k}_5} - \frac{\tilde{K}_1k_4k_6k_{1,0}}{\tilde{k}_3\tilde{k}_5}\Bigg) \Bigg/\\ &\Bigg(\Bigg(\tilde{K}_1^2k_{1,0}^2 + \frac{2\tilde{K}_1^2k_6k_{1,0}^2}{\tilde{k}_5} + \frac{\tilde{K}_1^2k_6^2k_{1,0}^2}{\tilde{k}_5^2} + \frac{\tilde{K}_1^2k_4^2k_6^2\left(k_{1,0}+k_2\right)^2}{(\tilde{k}_3\tilde{k}_5)^2} + \frac{2\tilde{K}_1^2k_4k_6^2k_{1,0}\left(k_{1,0}+k_2\right)}{\tilde{k}_3\tilde{k}_5^2} +\\ &\frac{2\tilde{K}_1^2k_4k_6k_{1,0}\left(k_{1,0}+k_2\right)}{\tilde{k}_3\tilde{k}_5} + \frac{4\tilde{K}_1k_2k_4k_6k_{1,0}}{\tilde{k}_3\tilde{k}_5}\Bigg)^{\frac{1}{2}} +\\ &\tilde{K}_1k_{1,0} + \frac{\tilde{K}_1k_6k_{1,0}}{\tilde{k}_5} + \frac{k_2k_4}{1\tilde{k}_3}\left(2\tilde{K}_1 + \frac{\tilde{K}_1k_6}{\tilde{k}_5} + 2\right) + \frac{\tilde{K}_1k_4k_{1,0}}{\tilde{k}_3}\left(\frac{k_6}{\tilde{k}_5} + 2\right)\Bigg)\end{aligned}$$

$$
\begin{aligned}
x_3(0) = \Bigg(\Bigg(& \tilde{K}_1^2 (k_{1,0})^2 + \frac{2\tilde{K}_1^2 k_6 k_{1,0}^2}{\tilde{k}_5} + \frac{\tilde{K}_1^2 k_6^2 k_{1,0}^2}{\tilde{k}_5^2} + \frac{\tilde{K}_1^2 k_4^2 k_6^2 (k_{1,0}+k_2)^2}{(\tilde{k}_3\tilde{k}_5)^2} + \\
& \frac{2\tilde{K}_1^2 k_4 k_6^2 k_{1,0} (k_{1,0}+k_2)}{\tilde{k}_3\tilde{k}_5^2} + \frac{2\tilde{K}_1^2 k_4 k_6 k_{1,0} (k_{1,0}+k_2)}{\tilde{k}_3\tilde{k}_5} \\
& + \frac{4\tilde{K}_1 k_2 k_4 k_6 k_{1,0}}{\tilde{k}_3\tilde{k}_5} \Bigg)^{\frac{1}{2}} + \tilde{K}_1 k_{1,0} + \frac{\tilde{K}_1 k_6 k_{1,0}}{\tilde{k}_5} - \frac{\tilde{K}_1 k_2 k_4 k_6}{1\tilde{k}_3\tilde{k}_5} \\
& - \frac{\tilde{K}_1 k_4 k_6 k_{1,0}}{\tilde{k}_3\tilde{k}_5} \Bigg) \Bigg/ \Bigg(\left(\frac{k_6}{\tilde{k}_5} + 1 \right) \Bigg(\tilde{K}_1^2 k_{1,0}^2 + \frac{2\tilde{K}_1^2 k_6 k_{1,0}^2}{\tilde{k}_5} + \frac{\tilde{K}_1^2 k_6^2 k_{1,0}^2}{\tilde{k}_5^2} + \\
& \frac{\tilde{K}_1^2 k_4^2 k_6^2 (k_{1,0}+k_2)^2}{(\tilde{k}_3\tilde{k}_5)^2} + \frac{2\tilde{K}_1^2 k_4 k_6^2 k_{1,0} (k_{1,0}+k_2)}{\tilde{k}_3\tilde{k}_5^2} + \\
& \frac{2\tilde{K}_1^2 k_4 k_6 k_{1,0} (k_{1,0}+k_2)}{\tilde{k}_3\tilde{k}_5} + \frac{4\tilde{K}_1 k_2 k_4 k_6 k_{1,0}}{\tilde{k}_3\tilde{k}_5} \Bigg)^{\frac{1}{2}} + \tilde{K}_1 k_{1,0} \left(\frac{k_6}{\tilde{k}_5} + 1 \right)^2 \\
& + \frac{k_2 k_4 k_6}{\tilde{k}_3\tilde{k}_5} \left(\tilde{K}_1 + \frac{\tilde{K}_1 k_6}{\tilde{k}_5} + 2 \right) + \frac{\tilde{K}_1 k_6 k_6 k_{1,0}}{\tilde{k}_3\tilde{k}_5} \left(\frac{k_6}{\tilde{k}_5} + 1 \right) \Bigg) .
\end{aligned}
$$

The observables are given by

$$
\begin{aligned}
y_{1,r} &= s_{1,r}[\text{pMEK}] \\
y_{2,r} &= s_{2,r}[\text{pERK}] ,
\end{aligned}
$$

for replicates $r = 1, \ldots, 4$. The indices for conditions and experiments are neglected, since the scaling and noise parameters do not differ for these. The input $\mathbf{u}$ describes the concentrations [sora] and [UO126] and the three different conditions are $u_1 = (0,0)^T$, $u_2 = (0,30)^T$, and $u_3 = (5,0)^T$. The parameters, which are estimated from the data, are

$$
\begin{aligned}
\mathbf{q} = \Bigg(& \frac{k_{1,0}}{k_{1,1}}, k_{1,1}, \tau_1, \tau_2, \frac{K_1}{[\text{ERK}]_0}, k_2, K_2, k_3[\text{Raf}]_0, K_3, k_4, k_5[\text{MEK}]_0, k_6, \\
& s_{1,2}, s_{1,3}, s_{1,4}, s_{2,1}, s_{2,2}, s_{2,3}, s_{2,4}, \sigma_{1,2}, \sigma_{1,3}, \sigma_{1,4}, \sigma_{2,1}, \sigma_{2,2}, \sigma_{2,3}, \sigma_{2,4} \Bigg)^T .
\end{aligned}
$$

with specific scaling and noise parameters for replicates and observables. The parameters boundaries for the optimization are

$$
\begin{aligned}
\log_{10}(\mathbf{q})_{\text{lb}} &= (-7, \ldots, -7)^T \\
\log_{10}(\mathbf{q})_{\text{ub}} &= (5, \ldots, 5)^T .
\end{aligned}
$$

We performed 500 multi-starts for the Gaussian and 1000 starts for the Laplace noise to obtain the optimal parameters. We calculated the profile likelihoods using the standard and the hierarchical approach (Figure S8). For this, we used higher upper boundaries (10^{10}) for the scaling and noise parameters in the standard approach. The standard approach underestimates the profiles due to convergence problems during optimization. The profiles lie under the profiles calculated by the hierarchical approach. A similar problem has been observed by Stapor et al. (2018a) when using only first-order derivative information. Interestingly, the improved convergence of the hierarchical approach allowed to calculate the profiles even without the employment of the Hessian. The resulting profiles are also in good agreement with the profiles calculated by Stapor et al. (2018a), proving again also numerically that the identifiability of the model is not influenced by the use of the hierarchical approach. For these calculations we employed the interior-point algorithm of fmincon, which provided for this model more reliable results than the trust-region algorithm. For Laplace noise, both approaches do not provide reliable profiles.

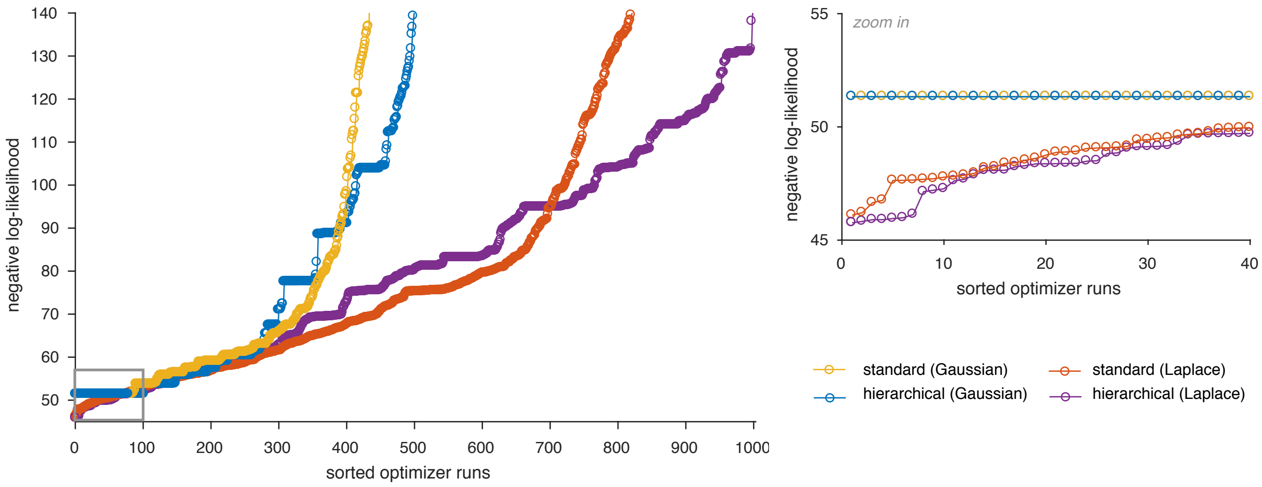

Supplementary Figure S7: Likelihood waterfall plot for RAF/MEK/ERK signaling.

Supplementary Table S4: **Optimization results for RAF/MEK/ERK signaling.** The maximum likelihood estimates (MLE) and 95% confidence interval (CI) are provided for the $\log_{10}$-parameter values for the standard (st.) and the hierarchical (hier.) approach.

parameter	Gaussian				Laplace			
	MLE [$\log_{10}$]		CI [$\log_{10}$]		MLE [$\log_{10}$]		CI [$\log_{10}$]	
	st.	hier.	st.	hier.	st.	hier.	st.	hier.
k_2	0.406	0.406	[0.338,0.565]	[0.0495,>5]	0.0101	-0.163	[-0.138,0.171]	[-0.365,0.369]
$k_{1,1}$	-1.59	-2.38	[-3.27,-1.58]	[<-7,>5]	2.38	-0.52	[2.11,2.93]	[<-7,4.65]
k_4	0.928	0.928	[0.906,1.27]	[0.151,>5]	0.57	0.73	[0.41,0.646]	[0.237,1.07]
$k_3[\mathrm{Raf}]_0$	3.13	1.13	[0.77,4.44]	[<-7,>5]	1.67	1.5	[0.253,2]	[<-7,>5]
k_6	-0.125	-0.125	[-0.148,-0.0991]	[-0.267,0.104]	1.26	1.23	[1.09,1.26]	[1.09,4.35]
$k_5[\mathrm{MEK}]_0$	1.67	4.46	[-0.381,1.71]	[-3.2,>5]	1.58	4.38	[1.42,1.86]	[-1.81,>5]
$\frac{K_1}{[\mathrm{ERK}]_0}$	-7	-7	[<-7,-3.2]	[<-7,-3.2]	-5.99	-6.98	[<-7,-4.57]	[-Inf,-3.72]
$\frac{k_{1,0}}{k_{1,1}}$	-1.44	-1.44	[-1.45,-1.42]	[-3.48,-1.32]	-3.6	-3.52	[-4.05,-3.56]	[-Inf,-2.79]
τ_1	-5.99	-6.35	[<-7,-2.64]	[<-7,1.35]	-3.4	-2.12	[-4.12,-2.19]	[<-7,2.14]
τ_2	0.163	0.163	[0.134,0.222]	[-0.102,0.272]	-2.45	-2.06	[-2.97,-2.2]	[-5.94,-1.58]
K_2	-0.0447	-0.0446	[-0.0823,0.0125]	[-0.292,0.18]	-0.102	-0.0946	[-0.223,-0.0495]	[-0.255,0.0732]
K_3	-1.25	-1.25	[-1.28,-1.21]	[-1.79,-1.03]	-0.608	-0.609	[-0.715,-0.588]	[-0.744,>5]

6 Normalization of relative data

An alternative approach to introduce scaling parameters in the model is to normalize the measurements $\hat{y}$ with respect to some reference/control measurements $\hat{y}_{\mathrm{ref}}$ and consider the observable

$$\bar{y} = \frac{\hat{y}}{\hat{y}_{\mathrm{ref}}}. \tag{13}$$

In the case of Gaussian distributed $\hat{y} \sim \mathcal{N}(\mu_{\hat{y}}, \sigma_{\hat{y}})$ and $\hat{y}_{\mathrm{ref}} \sim \mathcal{N}(\mu_{\hat{y}_{\mathrm{ref}}}, \sigma_{\hat{y}_{\mathrm{ref}}})$, Thomaseth and Radde (2016) showed that this ratio is distributed according to

$$p(\bar{y}) = \frac{b(\bar{y})d(\bar{y})}{a^3(\bar{y})\sqrt{2\pi}\sigma_{\hat{y}_{\mathrm{ref}}}\sigma_{\hat{y}}} \operatorname{erf}\left(\frac{b(\bar{y})}{\sqrt{2}a(\bar{y})}\right) + \frac{\exp\left(-\frac{c}{2}\right)}{a^2(\bar{y})\pi\sigma_{\hat{y}_{\mathrm{ref}}}\sigma_{\hat{y}}} \tag{14}$$

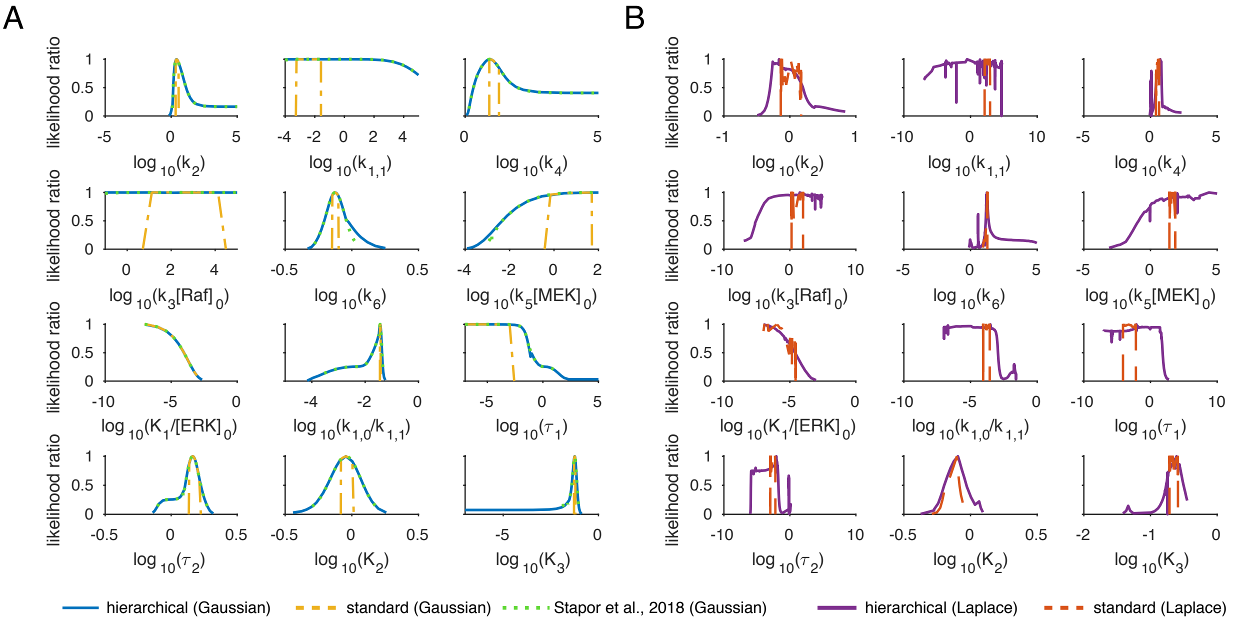

Supplementary Figure S8: Profile likelihoods for RAF/MEK/ERK signaling calculated for (A) Gaussian noise and (B) Laplace noise using the standard and hierarchical approach for optimization. For Gaussian noise also the results using hybrid profile calculation proposed by Stapor et al. (2018a) are shown.

with

$$a(\bar{y}) = \sqrt{\frac{1}{\sigma_{\hat{y}}^2}\bar{y}^2 + \frac{1}{\sigma_{\hat{y}_{\mathrm{ref}}}}}$$

$$b(\bar{y}) = \frac{\mu_{\hat{y}}}{\sigma_{\hat{y}}^2}\bar{y} + \frac{\mu_{\hat{y}_{\mathrm{ref}}}^2}{\sigma_{\hat{y}_{\mathrm{ref}}}^2}$$

$$c = \frac{\mu_{\hat{y}}^2}{\sigma_{\hat{y}}^2} + \frac{\mu_{\hat{y}_{\mathrm{ref}}}^2}{\sigma_{\hat{y}_{\mathrm{ref}}}}$$

$$d(\bar{y}) = \exp\left(\frac{b^2(\bar{y}) - ca^2(\bar{y})}{2a^2(\bar{y})}\right).$$

They also showed that the true mean can be approximated by

$$m_{\bar{y}} \approx \frac{\mu_{\hat{y}}}{\mu_{\hat{y}_{\mathrm{ref}}}} + \frac{\sigma_{\hat{y}_{\mathrm{ref}}}^2 \mu_{\hat{y}}}{\mu_{\hat{y}_{\mathrm{ref}}}^3}. \tag{15}$$

Due to the more complex structure of the noise distribution, it is often anyways assumed that (13) follows a Gaussian distribution (Degasperi et al., 2017). Here, we assessed the error made by this simplifying assumption, in particular the error of the obtained confidence intervals.

We generated 10^3 data sets of 10^2 samples of $\hat{y}$ and $\hat{y}_{\mathrm{ref}}$ as in (13), with $\mu_{\hat{y}} = 4, \sigma_{\hat{y}} = 1, \mu_{\hat{y}_{\mathrm{ref}}} = 4$, and, $\sigma_{\hat{y}_{\mathrm{ref}}} = 1$. We calculated the confidence intervals for $m_{\bar{y}}$ in (15), using (i) the ratio distribution defined in (14) and (ii) a Gaussian distribution

$$\bar{y} \sim \mathcal{N}(m_{\bar{y}}, \sigma_{\bar{y}}). \tag{16}$$

For case (i), we estimated the four parameters $\mu_{\hat{y}}, \sigma_{\hat{y}}, \mu_{\hat{y}_{\mathrm{ref}}}$, and, $\sigma_{\hat{y}_{\mathrm{ref}}}$ and calculated confidence intervals for (15) based on profile likelihoods and based on Markov chain Monte Carlo sampling for each of the 10^3 data sets. For case (ii), we

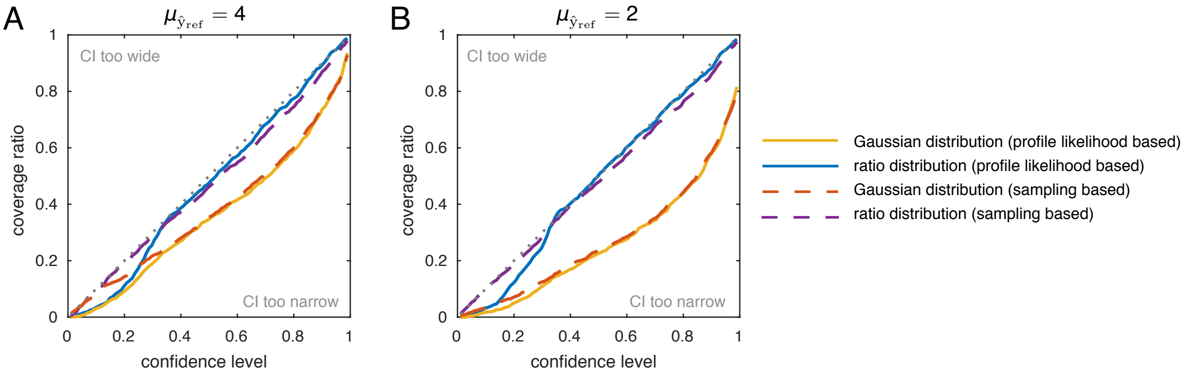

Supplementary Figure S9: Coverage ratio for the mean $m_{\bar{y}}$ of the ratio distribution evaluated using 10^3 data sets for the Gaussian distribution (16) and the ratio distribution (14) for (A) $\mu_{\hat{y}_{\rm ref}} = 4$ and (B) $\mu_{\hat{y}_{\rm ref}} = 2$.

estimated the two parameters $m_{\bar{y}}, \sigma_{\bar{y}}$ and also calculated confidence intervals for the mean based on profile likelihoods and sampling.

We assessed the appropriateness of the confidence intervals by computing the coverage ratio, which indicates for which fraction of the 10^3 data sets the true mean is within the boundaries of the confidence interval at a given confidence level (Figure S9A). The confidence intervals using the ratio distribution were more accurate, while the intervals obtained based on the Gaussian distribution were too narrow and underestimated the uncertainty. Repeating the procedure for $\mu_{\hat{y}_{\rm ref}} = 2$ even showed bigger differences when using the different likelihood functions (Figure S9B).

References

Bachmann, J., Raue, A., Schilling, M., Böhm, M. E., Kreutz, C., Kaschek, D., Busch, H., Gretz, N., Lehmann, W. D., Timmer, J., and Klingmüller, U. (2011). Division of labor by dual feedback regulators controls JAK2/STAT5 signaling over broad ligand range. *Mol. Syst. Biol.*, 7(1):516.

Degasperi, A., Fey, D., and Kholodenko, B. N. (2017). Performance of objective functions and optimisation procedures for parameter estimation in system biology models. *npj Syst Biol Appl*, 3(1):20.

Fiedler, A., Raeth, S., Theis, F. J., Hausser, A., and Hasenauer, J. (2016). Tailored parameter optimization methods for ordinary differential equation models with steady-state constraints. *BMC Syst. Biol.*, 10(80).

Fröhlich, F., Kaltenbacher, B., Theis, F. J., and Hasenauer, J. (2017). Scalable parameter estimation for genome-scale biochemical reaction networks. *PLoS Comput. Biol.*, 13(1):e1005331.

Maier, C., Loos, C., and Hasenauer, J. (2017). Robust parameter estimation for dynamical systems from outlier-corrupted data. *Bioinformatics*, 33(5):718–725.

Raue, A., Kreutz, C., Maiwald, T., Bachmann, J., Schilling, M., Klingmüller, U., and Timmer, J. (2009). Structural and practical identifiability analysis of partially observed dynamical models by exploiting the profile likelihood. *Bioinformatics*, 25(25):1923–1929.

Schelker, M., Raue, A., Timmer, J., and Kreutz, C. (2012). Comprehensive estimation of input signals and dynamics in biochemical reaction networks. *Bioinformatics*, 28(18):i529–i534.

Stapor, P., Fröhlich, F., and Hasenauer, J. (2018a). Optimization and profile calculation of ode models using second order adjoint sensitivity analysis. *Bioinformatics*, 34(13):i151–i159.

Stapor, P., Weindl, D., Ballnus, B., Hug, S., Loos, C., Fiedler, A., Krause, S., Hross, S., Fröhlich, F., and Hasenauer, J. (2018b). PESTO: Parameter EStimation TOolbox. *Bioinformatics*, 34(4):705–707.

Thomaseth, C. and Radde, N. (2016). Normalization of western blot data affects the statistics of estimators. *IFAC-PapersOnLine*, 49(26):56–62.

Vaz, A. I. F. and Vicente, L. N. (2009). PSwarm: A hybrid solver for linearly constrained global derivative-free optimization. *Optim. Method. Softw.*, 24(4-5):669–685.
