# Supplementary material for: Hierarchical optimization for the efficient parametrization of ODE models
Source: Bioinformatics. 2018 Jul 13;34(24):4266–73. doi: 10.1093/bioinformatics/bty514 (PMC6289139; doi:10.1093/bioinformatics/bty514)
Supplement: Supplementary Data [file bty514_supp.zip › bty514-suppl_data/PESTO.zip!/PESTO/bobyqa/Powell_Bobyqa.pdf]

DAMTP 2009/NA06

# The BOBYQA algorithm for bound constrained optimization without derivatives

M.J.D. Powell

**Abstract:** BOBYQA is an iterative algorithm for finding a minimum of a function $F(\underline{x})$, $\underline{x} \in \mathcal{R}^n$, subject to bounds $\underline{a} \leq \underline{x} \leq \underline{b}$ on the variables, $F$ being specified by a "black box" that returns the value $F(\underline{x})$ for any feasible $\underline{x}$. Each iteration employs a quadratic approximation $Q$ to $F$ that satisfies $Q(\underline{y}_j) = F(\underline{y}_j)$, $j = 1, 2, \ldots, m$, the interpolation points $\underline{y}_j$ being chosen and adjusted automatically, but $m$ is a prescribed constant, the value $m = 2n+1$ being typical. These conditions leave much freedom in $Q$, taken up when the model is updated by the highly successful technique of minimizing the Frobenius norm of the change to the second derivative matrix of $Q$. Thus no first derivatives of $F$ are required explicitly. Most changes to the variables are an approximate solution to a trust region subproblem, using the current quadratic model, with a lower bound on the trust region radius that is reduced cautiously, in order to keep the interpolation points well separated until late in the calculation, which lessens damage from computer rounding errors. Some other changes to the variables are designed to improve the model without reducing $F$. These techniques are described. Other topics include the starting procedure that is given an initial vector of variables, the value of $m$ and the initial trust region radius. There is also a new device called RESCUE that tries to restore normality if severe loss of accuracy occurs in the matrix calculations of the updating of the model. Numerical results are reported and discussed for two test problems, the numbers of variables being between 10 and 320.

Department of Applied Mathematics and Theoretical Physics,
Centre for Mathematical Sciences,
Wilberforce Road,
Cambridge CB3 0WA,
England.

August, 2009.

## 1. Introduction

BOBYQA is a package of Fortran subroutines that seeks the least value of an objective function $F(\underline{x})$, $\underline{x}\in\mathcal{R}^n$, subject to the simple bounds

$$a_i \le x_i \le b_i, \qquad i=1,2,\ldots,n, \tag{1.1}$$

on the components of $\underline{x}$. The user defines the objective function by another subroutine that returns the value $F(\underline{x})$ for any $\underline{x}$ in $\mathcal{R}^n$ that obeys the constraints (1.1). No derivatives of $F$ are required. The name BOBYQA is an acronym for Bound Optimization BY Quadratic Approximation.

The method of BOBYQA is iterative, $k$ and $n$ being reserved for the iteration number and the number of variables, respectively. Further, we reserve $m$ for the number of interpolation conditions that are imposed on a quadratic approximation $Q_k(\underline{x})$, $\underline{x}\in\mathcal{R}^n$, to $F(\underline{x})$, $\underline{x}\in\mathcal{R}^n$. The approximation is available at the beginning of the $k$-th iteration, the interpolation equations have the form

$$Q_k(\underline{y}_j) = F(\underline{y}_j), \qquad j=1,2,\ldots,m, \tag{1.2}$$

and $m$ is a constant integer from the interval $[n{+}2,\ \frac{1}{2}(n{+}1)(n{+}2)]$, chosen by the user of the software. We let $\underline{x}_k$ be the point in the set $\{\underline{y}_j : j=1,2,\ldots,m\}$ that has the property

$$F(\underline{x}_k) = \min\{F(\underline{y}_j) : j=1,2,\ldots,m\}, \tag{1.3}$$

any ties being broken by giving priority to an earlier evaluation of the least function value $F(\underline{x}_k)$. A positive number $\Delta_k$, called the "trust region radius", is also available at the beginning of the $k$-th iteration.

If certain conditions are achieved, as specified later, then termination occurs on the $k$-th iteration. Otherwise, a step $\underline{d}_k$ from $\underline{x}_k$ is constructed such that $\|\underline{d}_k\|\le\Delta_k$ holds, such that $\underline{x}=\underline{x}_k+\underline{d}_k$ is within the bounds (1.1), and such that $\underline{x}_k+\underline{d}_k$ is not one of the interpolation points $\underline{y}_j$, $j=1,2,\ldots,m$. Then the new function value $F(\underline{x}_k+\underline{d}_k)$ is calculated, and one of the interpolation points, $\underline{y}_t$ say, is replaced by $\underline{x}_k+\underline{d}_k$, where $\underline{y}_t$ is different from $\underline{x}_k$. It follows that $\underline{x}_{k+1}$ is defined by the formula

$$\underline{x}_{k+1} = \begin{cases} \underline{x}_k, & F(\underline{x}_k+\underline{d}_k) \ge F(\underline{x}_k), \\ \underline{x}_k+\underline{d}_k, & F(\underline{x}_k+\underline{d}_k) < F(\underline{x}_k). \end{cases} \tag{1.4}$$

Further, $\Delta_{k+1}$ and $Q_{k+1}$ are generated for the next iteration, $Q_{k+1}$ being subject to the constraints

$$Q_{k+1}(\widehat{\underline{y}}_j) = F(\widehat{\underline{y}}_j), \qquad j=1,2,\ldots,m, \tag{1.5}$$

at the new interpolation points

$$\widehat{\underline{y}}_j = \begin{cases} \underline{y}_j, & j\ne t, \\ \underline{x}_k+\underline{d}_k, & j=t, \end{cases} \qquad j=1,2,\ldots,m. \tag{1.6}$$

These features without the restrictions (1.1) are taken from the NEWUOA software (Powell, 2006) for unconstrained optimization without derivatives, as are several other features that receive attention later. We are trying to make the excellent efficiency of NEWUOA for large $n$ available to applications that include simple bounds on the variables. The use of quadratic models allows NEWUOA to provide high accuracy in many cases using far fewer than $\frac{1}{2}n^2$ function values altogether, although a quadratic function of $n$ variables has $\frac{1}{2}(n+1)(n+2)$ degrees of freedom. Let $\#F$ be the total number of calculations of values of $F$ and let $\underline{x}_*$ be the optimal vector of variables. The numerical results of Powell (2008) for NEWUOA, where the range of $n$ goes up to 320, show clearly that, if $m$ is set to $2n+1$, then often $\#F$ is only of magnitude $n$ or less, and it happens occasionally that smaller values of $m$ are even more efficient. In these cases, if $F$ is twice differentiable, one cannot expect $\nabla^2 Q_k$ to become a good approximation to $\nabla^2 F(\underline{x}_*)$, $\#F$ being far too small. Indeed, in a range of experiments when $F$ itself is quadratic, the final value of the Frobenius matrix norm $\|\nabla^2 F - \nabla^2 Q_k\|_F$ exceeds $\frac{1}{2}\|\nabla^2 F\|_F$, although $\|\underline{x}_k - \underline{x}_*\| \leq 10^{-6}\|\underline{x}_1 - \underline{x}_*\|$ is achieved at termination (Powell, 2009, to be published). We are employing the Frobenius matrix norm, because $Q_{k+1}$ is constructed from $Q_k$ by a version of the symmetric Broyden formula that has the property

$$\|\nabla^2 F - \nabla^2 Q_{k+1}\|_F \leq \|\nabla^2 F - \nabla^2 Q_k\|_F, \qquad k=1,2,3,\ldots, \tag{1.7}$$

when $F$ is quadratic.

The methods of NEWUOA and BOBYQA are the only algorithms known to the author for optimization without derivatives that employ quadratic models, and that take up the freedom in $Q_{k+1}$ by minimizing $\|\nabla^2 Q_{k+1} - \nabla^2 Q_k\|_F$, after satisfying the interpolation conditions (1.5) with $\nabla^2 Q_{k+1}$ symmetric. The reason for trying this technique originally was that the calculation of $Q_{k+1}$ from $Q_k$ requires only $\mathcal{O}(n^2)$ operations in the case $m = 2n+1$, but $\mathcal{O}(n^4)$ operations are needed if $Q_{k+1}$ is defined completely by the interpolation conditions (1.5), the value of $m$ being $\frac{1}{2}(n+1)(n+2)$. It was not expected then that the reduction in $m$ would provide the huge improvement to $\#F$ that is reported above. The author's knowledge of convergence theory had nothing to do with that success, and it remains unhelpful to the present work. Thus NEWUOA and BOBYQA provide a counter-example to the suggestion in Gould and Toint (2004) that theoretical insight is of vital importance to the development of good numerical methods. Some interesting work on the theory of quadratic models for minimization without derivatives can be found in Conn, Scheinberg and Vicente (2009), but it does not address algorithms that achieve high accuracy with $\#F$ substantially less than $\mathcal{O}(n^2)$.

The operations of BOBYQA that prepare for the first iteration are described in Section 2. They require the user to provide an initial vector of variables $\underline{x}_0 \in \mathcal{R}^n$ and the initial trust region radius $\Delta_1$, in addition to the bounds of expression (1.1) and the value of $m$. The choice of $\underline{d}_k$ is specified in Section 3. On "trust region"

iterations, $\underline{d}_k$ is a convenient estimate of the vector $\underline{d}$ that solves the subproblem

$$\left.\begin{array}{lll} \text{Minimize} & Q_k(\underline{x}_k+\underline{d}), & \underline{d}\in\mathcal{R}^n, \\ \text{subject to} & \underline{a} \le \underline{x}_k+\underline{d} \le \underline{b} \quad \text{and} & \|\underline{d}\| \le \Delta_k \end{array}\right\}. \tag{1.8}$$

There are also "alternative" iterations, however, and then $\underline{d}_k$ is chosen in a way that promotes good linear independence in the interpolation conditions (1.5).

Two updating procedures are addressed in Section 4, one of them being the calculation of $Q_{k+1}$ from $Q_k$. The change $Q_{k+1}-Q_k$ to the quadratic model is defined by an $(m+n+1)\times(m+n+1)$ system of linear equations, which is solved in only $\mathcal{O}(m^2)$ operations, due to the construction of the inverse of the matrix of this system from the previous inverse by the other updating procedure. Let $\Omega_k$ be the leading $m\times m$ submatrix of the inverse matrix. It is important to numerical stability that in theory $\Omega_k$ can be expressed as the product

$$\Omega_k = Z_k\, Z_k^T, \tag{1.9}$$

where $Z_k$ is a real matrix with $m$ rows but only $m$–$n$–1 columns. It is possible, and rare, for an accumulation of rounding errors to introduce a negative eigenvalue into $\Omega_{k+1}$. In this case NEWUOA would express $\Omega_{k+1}$ in the form $Z_{k+1}S_{k+1}Z_{k+1}^T$, $Z_{k+1}$ being $m\times(m–n–1)$ as usual, while $S_{k+1}$ is an $(m–n–1)\times(m–n–1)$ diagonal matrix with each diagonal element set to $-1$ or $+1$. The response of BOBYQA to a negative eigenvalue, however, is to move a few interpolation points if necessary to restore the factorization $\Omega_{k+1}=Z_{k+1}Z_{k+1}^T$. Details of this new device, which has the name RESCUE, are given in Section 5.

Several other subjects are considered briefly in Section 6. They include the selection of $t$ for formula (1.6), the calling of RESCUE, the adjustment of the trust region radius, the choice between "trust region" and "alternative" iterations, the conditions for termination, and shifts of the origin. Finally, Section 7 provides numerical results for two of the test problems in Powell (2008), namely "trigonometric sum of squares" and "points in square". The bounds (1.1) are irrelevant in the first example, its purpose being to demonstrate that $\#F$ can be $\mathcal{O}(n)$ for large $n$. The purpose of the second example is to show the robustness and some limitations of BOBYQA in a difficult case where $F$ has several local minima.

## 2. Preliminary calculations

It has been stated already that the user has to supply an initial vector of variables $\underline{x}_0\in\mathcal{R}^n$, the vectors $\underline{a}$ and $\underline{b}$ whose components are the bounds of expression (1.1), the initial trust region radius $\Delta_1$, and the number $m$ of interpolation conditions, where $n+2 \le m \le \frac{1}{2}(n+1)(n+2)$. A gradient of the first quadratic model is constructed from the changes that occur in $F$ when steps from $\underline{x}_0$ parallel to coordinate directions are taken in $\mathcal{R}^n$, the lengths of these steps being $\Delta_1$. When there are two such steps in the same direction, they provide a diagonal element of the

second derivative matrix $\nabla^2 Q_1$. Because room is required for these constructions, an error return is made immediately from BOBYQA if the bounds fail to satisfy the conditions

$$b_i \;\geq\; a_i + 2\,\Delta_1, \qquad i=1,2,\ldots,n. \tag{2.1}$$

The position of $\underline{x}_0$ also has to be suitable for these constructions, and if necessary it is altered automatically without an error return. Let $i$ run through the integers $1,2,\ldots,n$, and let $(\underline{x}_0)_i$ be the $i$-th component of the given vector $\underline{x}_0$. This component is overwritten by $a_i$ or $b_i$ in the case $(\underline{x}_0)_i < a_i$ or $(\underline{x}_0)_i > b_i$, respectively. Moreover, it is overwritten by $a_i+\Delta_1$ or $b_i-\Delta_1$ in the case $a_i < (\underline{x}_0)_i < a_i+\Delta_1$ or $b_i-\Delta_1 < (\underline{x}_0)_i < b_i$, respectively, In all other cases, the original value of $(\underline{x}_0)_i$ is retained.

We are now ready to specify the interpolation points $\underline{y}_j$, $j=1,2,\ldots,m$, of the first quadratic model, these points being the same as in NEWUOA if the current $\underline{x}_0$ is not on the boundary of any constraint. We set $\underline{y}_1=\underline{x}_0$, and, for $i=1,2,\ldots,n$, we define $\underline{y}_{i+1}$ and $\underline{y}_{n+i+1}$ by the formula

$$\left.\begin{array}{lllll}
\underline{y}_{i+1} = \underline{x}_0 + \Delta_1\,\underline{e}_i & \text{and} & \underline{y}_{n+i+1} = \underline{x}_0 - \Delta_1\,\underline{e}_i, & & a_i < (\underline{x}_0)_i < b_i \\
\underline{y}_{i+1} = \underline{x}_0 + \Delta_1\,\underline{e}_i & \text{and} & \underline{y}_{n+i+1} = \underline{x}_0 + 2\,\Delta_1\,\underline{e}_i, & & (\underline{x}_0)_i = a_i \\
\underline{y}_{i+1} = \underline{x}_0 - \Delta_1\,\underline{e}_i & \text{and} & \underline{y}_{n+i+1} = \underline{x}_0 - 2\,\Delta_1\,\underline{e}_i, & & (\underline{x}_0)_i = b_i
\end{array}\right\}, \tag{2.2}$$

where $\underline{e}_i$ is the $i$-th coordinate vector in $\mathcal{R}^n$. If $m \leq 2n+1$, then the interpolation points of the first quadratic model are $\underline{y}_j$, $j=1,2,\ldots,m$, the definitions of $\underline{y}_j$, $j>m$, being superfluous. The function values $F(\underline{y}_j)$, $j=1,2,\ldots,\min[m,\, 2n+1]$, are calculated.

When $m > 2n+1$, the points $\underline{y}_j$, $j=1,2,\ldots,2n+1$, are taken from the previous paragraph, but they may be reordered a little. Specifically, $\underline{y}_{i+1}$ and $F(\underline{y}_{i+1})$ are exchanged with $\underline{y}_{n+i+1}$ and $F(\underline{y}_{n+i+1})$ for all integers $i$ in $[1,n]$ that satisfy both $a_i < (\underline{x}_0)_i < b_i$ and $F(\underline{y}_{n+i+1}) < F(\underline{y}_{i+1})$, which provides a bias towards lower values of $F$ in the following construction. The last $m-2n-1$ interpolation points of $Q_1$ have the form

$$\underline{y}_j \;=\; \underline{y}_{p(j)+1} + \underline{y}_{q(j)+1} - \underline{x}_0, \qquad 2n+2 \leq j \leq m, \tag{2.3}$$

where $p(j)$ and $q(j)$ are different integers from $[1,n]$. Equations (2.2) and (2.3) show that the $p(j)$-th and $q(j)$-th components of $\underline{y}_j-\underline{x}_0$ have modulus $\Delta_1$, all the other components of $\underline{y}_j-\underline{x}_0$ being zero. The values of $p(j)$, $j \geq 2n+2$, are given by the formula

$$p(j) \;=\; \left\{\begin{array}{ll} j-2n-1, & 2n+2 \leq j \leq 3n+1, \\ p(j-n), & 3n+2 \leq j \leq m, \end{array}\right. \tag{2.4}$$

so they cycle through the sequence $\{1,2,\ldots,n\}$. Further, $q(j)$ is set to $p(j)+\ell$ or $p(j)+\ell-n$ during the $\ell$-th of these cycles, the choice between these alternatives

being settled by $1 \le q(j) \le n$. For example, if $n=5$ and $m=20$, there are 9 pairs $\{p(j), q(j)\}$, generated in the order $\{1,2\}$, $\{2,3\}$, $\{3,4\}$, $\{4,5\}$, $\{5,1\}$, $\{1,3\}$, $\{2,4\}$, $\{3,5\}$ and $\{4,1\}$, as mentioned in Powell (2006). The function values $F(\underline{y}_j)$, $j=2n+2, 2n+3, \ldots, m$, are also calculated.

We now have all the data for the interpolation equations (1.2) when $k=1$. In order to specify the $\frac{1}{2}(n+1)(n+2)-m$ remaining degrees of freedom in $Q_1$, we write the first quadratic model in the form

$$Q_1(\underline{x}_0+\underline{s}) \;=\; Q_1(\underline{x}_0) + \sum_{i=1}^{n} (\underline{g}_0)_i \, s_i + \tfrac{1}{2} \sum_{p=1}^{n} \sum_{q=1}^{n} (\nabla^2 Q_1)_{pq} \, s_p \, s_q, \qquad \underline{s} \in \mathcal{R}^n, \quad (2.5)$$

where $\underline{g}_0$ is the gradient $\underline{\nabla} Q_1(\underline{x}_0)$. The first interpolation condition with $\underline{y}_1=\underline{x}_0$ gives $Q_1(\underline{x}_0)=F(\underline{y}_1)$. Then formula (2.2) implies that, for every integer $i$ in the interval $[1, \min\{n, m-n-1\}]$, the coefficients $(\underline{g}_0)_i$ and $(\nabla^2 Q_1)_{ii}$ are defined by the conditions $Q_1(\underline{x}_0)=F(\underline{y}_1)$, $Q_1(\underline{y}_{i+1})=F(\underline{y}_{i+1})$ and $Q_1(\underline{y}_{n+i+1})=F(\underline{y}_{n+i+1})$. Further, if $m<2n+1$, then, for every integer $i$ in $[m-n, n]$, we set $(\nabla^2 Q_1)_{ii}=0$, so now $(\underline{g}_0)_i$ is defined by $Q_1(\underline{x}_0)=F(\underline{y}_1)$ and $Q_1(\underline{y}_{i+1})=F(\underline{y}_{i+1})$. On the other hand, if the interpolation point (2.3) is required due to $m \ge 2n+2$, and if we put $\underline{s}=\underline{y}_j-\underline{x}_0$ into expression (2.5), then all the nonzero terms on the right hand side of the expression are known, except for the contributions from $\{p,q\}=\{p(j), q(j)\}$. Thus the two second derivatives $(\nabla^2 Q_1)_{p(j)\,q(j)} = (\nabla^2 Q_1)_{q(j)\,p(j)}$ are derived from $Q_1(\underline{y}_j)=F(\underline{y}_j)$. The remaining off-diagonal elements $(\nabla^2 Q_1)_{pq}$, $p \ne q$, when $\{p,q\}$ is not one of the pairs $\{p(j), q(j)\}$, $2n+2 \le j \le m$, are set to zero, which completes the description of the first quadratic model. One can take the view that the freedom in $Q_1$ has been taken up by minimizing the Frobenius norm $\|\nabla^2 Q_1\|_F$.

We recall from Section 1 that the inverse of the matrix of a linear system of equations is employed on each iteration to assist the calculation of the next quadratic model $Q_{k+1}$ from $Q_k$. The construction of this inverse for the first iteration, which is also a part of the preliminary work, is described next. The linear system is square and has the partitioned form

$$\left(\begin{array}{c|c} A & Y^T \\ \hline Y & 0 \end{array}\right) \left(\begin{array}{c} \underline{\lambda} \\ \hline c \\ \underline{g} \end{array}\right) \;=\; \left(\begin{array}{c} \underline{r} \\ \hline 0 \end{array}\right) \quad \begin{array}{l} \updownarrow\ m \\ \updownarrow\ n+1\,, \end{array} \qquad (2.6)$$

as in expression (3.10) of Powell (2006). In this section we study the symmetric matrices

$$W \;=\; \left(\begin{array}{c|c} A & Y^T \\ \hline Y & 0 \end{array}\right) \qquad \text{and} \qquad H \;=\; W^{-1} \;=\; \left(\begin{array}{c|c} \Omega & \Xi^T \\ \hline \Xi & \Upsilon \end{array}\right), \qquad (2.7)$$

taking advantage of the structure that comes from the initial positions of the interpolation points. Further attention is given to the system (2.6) in Section 4. The $m \times m$ symmetric matrix $A$ has the elements

$$A_{ij} \;=\; \tfrac{1}{2} \, \{(\underline{y}_i - \underline{x}_0)^T (\underline{y}_j - \underline{x}_0)\}^2, \qquad 1 \le i, j \le m, \qquad (2.8)$$

while $Y$ is the $(n+1)\times m$ matrix

$$Y \;=\; \begin{pmatrix} 1 & 1 & \cdots & 1 \\ \underline{y}_1 - \underline{x}_0 & \underline{y}_2 - \underline{x}_0 & \cdots & \underline{y}_m - \underline{x}_0 \end{pmatrix}, \tag{2.9}$$

which completes the specification of the data that define the matrix $H = W^{-1}$ for the first iteration.

Similar interpolation points and matrices occur in the technique of Section 5, except that, for $i = 1, 2, \ldots, n$, the modulus of the nonzero component of $\underline{y}_{i+1} - \underline{x}_0$ may be different from $\Delta_k$. We make the present work relevant to Section 5 by writing expression (2.2) in the form

$$\underline{y}_{i+1} = \underline{x}_0 + \alpha_i\, \underline{e}_i \quad \text{and} \quad \underline{y}_{n+i+1} = \underline{x}_0 + \beta_i\, \underline{e}_i, \qquad i = 1, 2, \ldots, n, \tag{2.10}$$

where the multipliers $\alpha_i$ and $\beta_i$ are assumed to be any nonzero numbers that satisfy $\alpha_i \neq \beta_i$, except that $\beta_i$ and $\underline{y}_{n+i+1}$ are not required if $n+i+1$ exceeds $m$. This increase in generality preserves the validity of every statement in the paragraph that includes equation (2.5). We retain formula (2.3) without altering $p(j)$ and $q(j)$, and also we keep the definitions (2.8) and (2.9) of the submatrices $A$ and $Y$.

The elements of the submatrices $\Xi$ and $\Upsilon$, introduced in expression (2.7), are written down explicitly below, with the elements of an $m \times (m-n-1)$ matrix $Z$ such that $\Omega$ is the product $ZZ^T$. Checking the correctness of the given values is left as an exercise for the reader. These tasks are possible, because of the sparseness and structure that are provided by equations (2.10), (2.3), (2.8) and (2.9) with $\underline{y}_1 = \underline{x}_0$. In particular, $\underline{y}_1 - \underline{x}_0$ is the zero vector, and $\underline{y}_i - \underline{x}_0$ has only one or two nonzero components in the cases $2 \leq i \leq 2n+1$ or $i \geq 2n+2$, respectively.

The dimensions of $\Xi$ are $(n+1)\times m$. Its first row is the first coordinate vector in $\mathcal{R}^m$, because the first row of $W$ is the $(m+1)$-th coordinate vector of $\mathcal{R}^{m+n+1}$. For $1 \leq i \leq \min[n,\, m-n-1]$, the $(i+1)$-th row of $\Xi$ has exactly three nonzero elements that take the values

$$\Xi_{i+1\,1} = -\frac{1}{\alpha_i} - \frac{1}{\beta_i}, \quad \Xi_{i+1\,i+1} = \frac{\beta_i}{\alpha_i\,(\beta_i - \alpha_i)} \quad \text{and} \quad \Xi_{i+1\,n+i+1} = \frac{\alpha_i}{\beta_i\,(\alpha_i - \beta_i)}. \tag{2.11}$$

For $m \leq 2n$ and $m-n \leq i \leq n$, the $(i+1)$-th row of $\Xi$ has only the two nonzero elements

$$\Xi_{i+1\,1} = -\frac{1}{\alpha_i} \qquad \text{and} \qquad \Xi_{i+1\,i+1} = \frac{1}{\alpha_i}, \tag{2.12}$$

which completes the description of $\Xi$. All the elements of the initial $(n+1)\times(n+1)$ submatrix $\Upsilon$ are zero in the case $m \geq 2n+1$. Otherwise, $\Upsilon$ has only $2n-m+1$ nonzero elements, and they are the diagonal entries

$$\Upsilon_{i+1\,i+1} = -\tfrac{1}{2}\,\alpha_i^2, \qquad m-n \leq i \leq n. \tag{2.13}$$

The freedom to post-multiply $Z_k$ by any $(m-n-1)\times(m-n-1)$ orthogonal matrix in equation (1.9) allows the $m\times(m-n-1)$ matrix $Z$ of the first iteration

to have a convenient property. It is that the square matrix whose rows are the last $(m-n-1)$ rows of $Z$ is diagonal. Thus, for $1 \le \ell \le \min[n,\, m-n-1]$, there are exactly three nonzero entries in the $\ell$-th column of $Z$ with the values

$$Z_{1\,\ell} = \frac{\sqrt{2}}{\alpha_\ell\,\beta_\ell}, \quad Z_{\ell+1\,\ell} = \frac{\sqrt{2}}{\alpha_\ell\,(\beta_\ell - \alpha_\ell)} \quad \text{and} \quad Z_{n+\ell+1\,\ell} = \frac{\sqrt{2}}{\beta_\ell\,(\alpha_\ell - \beta_\ell)}. \tag{2.14}$$

Further, if $Z$ has more than $n$ columns, then, for $n+1 \le \ell \le m-n-1$, the $\ell$-th column of $Z$ has the four nonzero elements

$$Z_{1\,\ell} = Z_{n+\ell+1\,\ell} = \frac{1}{\alpha_{p(j)}\,\alpha_{q(j)}} \quad \text{and} \quad Z_{p(j)+1\,\ell} = Z_{q(j)+1\,\ell} = \frac{-1}{\alpha_{p(j)}\,\alpha_{q(j)}}, \tag{2.15}$$

the indices $p(j)$ and $q(j)$ being taken from equation (2.3) in the case $j=n+\ell+1$. The description of $Z$ is complete.

By applying the remarks of the last two paragraphs, it is straightforward to generate the required inverse matrix $W^{-1}$ for the first iteration, keeping the leading $m\times m$ submatrix $\Omega$ in the factored form (1.9).

## 3. The choice of $\underline{d}_k$

Both "trust region" and "alternative" iterations are mentioned in Section 1. The techniques they employ to construct the step $\underline{d}_k$ from $\underline{x}_k$ are different and are described in this section. In both cases, the step satisfies the constraints

$$\underline{a} \le \underline{x}_k + \underline{d}_k \le \underline{b} \qquad \text{and} \qquad \|\underline{d}_k\| \le \Delta_k. \tag{3.1}$$

One complication is that, if $\underline{d}_k$ is generated by the trust region procedure, and if $\|\underline{d}_k\|$ is less than $\frac{1}{2}\Delta_k$, then, instead of calculating $F(\underline{x}_k+\underline{d}_k)$, the current iteration may be replaced by an "alternative" iteration. Thus BOBYQA postpones the use of short steps, because smaller steplengths $\|\underline{d}_k\|$ in the conditions $Q_{k+1}(\underline{x}_k)=F(\underline{x}_k)$ and $Q_{k+1}(\underline{x}_k+\underline{d}_k)=F(\underline{x}_k+\underline{d}_k)$ tend to increase the damage to $Q_{k+1}$ from various possible errors. Further attention is given to switches between the two types of iteration in Section 6, so now only the two choices of $\underline{d}_k$ are described.

Another complication is shifts of origin. Often the interpolation points $\underline{y}_j$, $j=1,2,\ldots,m$, including $\underline{x}_k$, are in a cluster whose diameter is of magnitude $\Delta_k$. Then, in order to avoid much cancellation in the differences $\underline{y}_i-\underline{y}_j$, $i \ne j$, it is helpful if the distance from $\underline{x}_k$ to the origin has this magnitude too. In the usual case when $\Delta_k$ becomes small as the iterations proceed, however, we do not expect this property to hold, unless the position of the origin is adapted automatically to the progress of the iterations. We reserve $\underline{x}_0$ for the current position of the origin, which agrees with the preliminary calculations of Section 2, as shown in the definitions (2.8) and (2.9). Occasionally $\underline{x}_0$ becomes the current $\underline{x}_k$, but this change is made rarely, because the amount of computation of each shift of origin is $\mathcal{O}(m^2 n)$, as explained in Section 6. Advantage is taken of the shift by working

with $\underline{a}-\underline{x}_0$, $\underline{b}-\underline{x}_0$ and $\underline{x}_k-\underline{x}_0$, instead of with $\underline{a}$, $\underline{b}$ and $\underline{x}_k$, when $\underline{d}_k$ is calculated. It is important in practice to ensure that, if any of the constraints

$$\underline{a}-\underline{x}_0 \;\leq\; (\underline{x}_k-\underline{x}_0)+\underline{d}_k \;\leq\; \underline{b}-\underline{x}_0 \tag{3.2}$$

are satisfied as equations, then the corresponding constraints (3.1) hold also as equations. We simplify the remainder of this section by assuming $\underline{x}_0=0$, which does not lose generality, because the following descriptions are in terms of exact arithmetic.

The calculation of the "trust region" step $\underline{d}_k$ is done by subroutine TRSBOX of BOBYQA, the name being an acronym for Trust Region Step in the BOX defined by expression (3.2). The vector $\underline{d}$ of the subproblem (1.8) is adjusted by an active set version of the truncated conjugate gradient procedure, beginning at the centre $\underline{d}=0$ of the trust region $\{\underline{d} : \|\underline{d}\| \leq \Delta_k\}$, with a restart and an enlarged active set if $\underline{d}$ becomes restricted by an additional side of the box. There is no removal of indices from the active set of the current subproblem. If $\underline{d}$ reaches the boundary of the trust region, the alternative being termination of the conjugate gradient iterations with $\|\underline{d}\|<\Delta_k$, then further changes may be made to $\underline{d}$, staying on the boundary $\|\underline{d}\|=\Delta_k$. Let $\mathcal{I}$ contain the indices of the components of $\underline{d}$ that are fixed at bounds by the active set method, and, for any $\underline{v}$ in $\mathcal{R}^n$, let $\mathcal{P}_{\mathcal{I}}(\underline{v})$ be the vector in $\mathcal{R}^n$ whose $i$-th component, $1\leq i\leq n$, is $v_i$ or zero if $i\notin\mathcal{I}$ or $i\in\mathcal{I}$, respectively. Each further change to $\underline{d}$ on the boundary of the trust region is in the two dimensional space spanned by $\mathcal{P}_{\mathcal{I}}(\underline{d})$ and $\mathcal{P}_{\mathcal{I}}(\underline{\nabla} Q_k(\underline{x}_k+\underline{d}))$ for the current $\underline{d}$. Details of these constructions are given below.

Let $(\underline{x}_k)_i$ be at its lower bound $a_i$. If $\underline{d}$ becomes nonzero in $\underline{x}_k+\underline{d}$, only zero or positive values of the component $d_i$ are allowed. Further, $d_i>0$ provides a first order reduction in $Q_k(\underline{x}_k+\underline{d})$ if and only if $(\underline{g}_k)_i<0$ holds, where $\underline{g}_k=\underline{\nabla} Q_k(\underline{x}_k)$. Therefore, in the unfavourable case $(\underline{g}_k)_i\geq 0$, we fix $d_i$ at zero by putting the index $i$ into $\mathcal{I}$. Specifically, the initial active set $\mathcal{I}$ contains the integers $i$ in $[1,n]$ that have the properties

$$\left.\begin{array}{llll} \text{either} & (\underline{x}_k)_i = a_i & \text{and} & (\underline{g}_k)_i \geq 0 \\ \text{or} & (\underline{x}_k)_i = b_i & \text{and} & (\underline{g}_k)_i \leq 0 \end{array}\right\}. \tag{3.3}$$

Termination has to occur with $\underline{d}_k=0$ if every $i\in\{1,2,\ldots,n\}$ is in the initial active set. Usually, however, $\underline{s}=-\mathcal{P}_{\mathcal{I}}(\underline{g}_k)$ is nonzero, and it is chosen to be the first search direction of the conjugate gradient procedure.

On every step along a search direction by this procedure, the $\underline{d}$ at the beginning of the step is strictly inside the trust region and the bounds (1.1) are satisfied at $\underline{x}=\underline{x}_k+\underline{d}$. Further, the chosen search direction $\underline{s}$ has both the zero components $s_i=0$, $i\in\mathcal{I}$, and the descent property $\underline{s}^T\underline{\nabla} Q_k(\underline{x}_k+\underline{d})<0$. Let $\alpha_B$ be the largest number such that $\underline{a}\leq\underline{x}_k+\underline{d}+\alpha_B\underline{s}\leq\underline{b}$ holds, let $\alpha_\Delta$ be the largest number such that $\|\underline{d}+\alpha_\Delta\underline{s}\|\leq\Delta_k$ is retained, and let $\alpha_Q$ (which may be infinite) be the largest number such that $Q_k(\underline{x}_k+\underline{d}+\alpha\underline{s})$, $0\leq\alpha\leq\alpha_Q$, decreases monotonically. These numbers are calculated, the chosen steplength $\alpha$ being the least of them, and $\underline{d}$ is overwritten by $\underline{d}+\alpha\underline{s}$.

In the case $\alpha = \alpha_\Delta$, the trust region boundary has been reached, which completes the iterations of the conjugate gradient method; further changes may be made to $\underline{d}$ as mentioned already. In the case $\alpha < \alpha_\Delta$ and $\alpha = \alpha_B$, the current line search is restricted by a bound constraint. Its index is added to $\mathcal{I}$ so that subsequent choices of $\underline{x}_k+\underline{d}$ remain on the boundary of the additional active constraint. At this stage, $Q(\underline{x}_k)-Q(\underline{x}_k+\underline{d})$ is the total reduction in $Q_k$ that has occurred so far, and the product $\|\mathcal{P}_\mathcal{I}(\underline{\nabla} Q_k(\underline{x}_k+\underline{d}))\| \, \Delta_k$ is likely to be an upper bound on any further reductions. Therefore termination with $\underline{d}_k$ set to the current $\underline{d}$ occurs if the condition

$$\|\mathcal{P}_\mathcal{I}(\underline{\nabla} Q_k(\underline{x}_k+\underline{d}))\| \, \Delta_k \;\leq\; 0.01 \, \{Q_k(\underline{x}_k) - Q_k(\underline{x}_k+\underline{d})\} \tag{3.4}$$

is achieved, because the effort of more iterations does not seem to be worthwhile. Otherwise, the conjugate gradient method is restarted at the current point $\underline{x}_k+\underline{d}$ with $\underline{s} = -\mathcal{P}_\mathcal{I}(\underline{\nabla} Q_k(\underline{x}_k+\underline{d}))$ as the next search direction. In the remaining case $\alpha < \alpha_\Delta$, $\alpha < \alpha_B$ and $\alpha = \alpha_Q$, the change from $\underline{d}$ to $\underline{d}+\alpha\underline{s}$ is a full projected conjugate gradient step without any interference from constraints, which gives a strict reduction in $Q_k$. If this reduction is at most the right hand side of expression (3.4), or if inequality (3.4) holds at the new point $\underline{x}_k+\underline{d}$, then termination also occurs with $\underline{d}_k$ set to the current $\underline{d}$. The alternative is a line search from the new point along a direction $\underline{s}$, chosen in a way that is usual for the conjugate gradient method, and having the properties stated in the previous paragraph. Specifically, $\underline{s}$ is the projected steepest descent direction $-\mathcal{P}_\mathcal{I}(\underline{\nabla} Q_k(\underline{x}_k+\underline{d}))$ augmented by the multiple of the previous search direction that gives orthogonality to the change in $\underline{\nabla} Q_k$ that occurred on the previous iteration. The description of the conjugate gradient iterations is complete.

If $\underline{d}$ is going to be moved round the trust region boundary, the components $d_i$, $i \in \mathcal{I}$, remain fixed as usual, and a substantial first order reduction in $Q_k(\underline{x}_k+\underline{d})$ is possible if and only if both $\|\mathcal{P}_\mathcal{I}(\underline{\nabla} Q_k(\underline{x}_k+\underline{d}))\|$ and the angle between $\mathcal{P}_\mathcal{I}(\underline{d})$ and $-\mathcal{P}_\mathcal{I}(\underline{\nabla} Q_k(\underline{x}_k+\underline{d}))$ are sufficiently large. Therefore the current $\underline{d}$ is returned as the solution of subproblem (1.8) if it satisfies the termination condition

$$\begin{aligned} \|\mathcal{P}_\mathcal{I}(\underline{d})\|^2 \, \|\mathcal{P}_\mathcal{I}(\underline{\nabla} Q_k(\underline{x}_k+\underline{d}))\|^2 \;-\; \{\mathcal{P}_\mathcal{I}(\underline{d})^T \mathcal{P}_\mathcal{I}(\underline{\nabla} Q_k(\underline{x}_k+\underline{d}))\}^2 & \\ \leq\; 10^{-4} \, \{Q_k(\underline{x}_k) - Q_k(\underline{x}_k+\underline{d})\}^2. & \end{aligned} \tag{3.5}$$

Otherwise, $\underline{s}$ is set to the vector in the two dimensional linear space spanned by $\mathcal{P}_\mathcal{I}(\underline{d})$ and $\mathcal{P}_\mathcal{I}(\underline{\nabla} Q_k(\underline{x}_k+\underline{d}))$ that has the properties $\|\underline{s}\| = \|\mathcal{P}_\mathcal{I}(\underline{d})\|$, $\underline{s}^T \mathcal{P}_\mathcal{I}(\underline{d}) = 0$ and $\underline{s}^T \mathcal{P}_\mathcal{I}(\underline{\nabla} Q_k(\underline{x}_k+\underline{d})) < 0$. Then $\underline{d}$ is moved round the trust region boundary by letting $\theta$ become positive in the expression

$$\underline{d}(\theta) = \underline{d} - \mathcal{P}_\mathcal{I}(\underline{d}) + \cos\theta \, \mathcal{P}_\mathcal{I}(\underline{d}) + \sin\theta \, \underline{s}, \qquad 0 \leq \theta \leq \tfrac{1}{4}\pi, \tag{3.6}$$

the $\underline{d}$ on the right hand side being the one at the beginning of the move. The length $\|\underline{d}(\theta)\| = \Delta_k$ is preserved, because the definition of $\mathcal{P}_\mathcal{I}$ implies that both $\mathcal{P}_\mathcal{I}(\underline{d})$ and $\underline{s}$ are orthogonal to $\underline{d}-\mathcal{P}_\mathcal{I}(\underline{d})$. Let $\theta_B$ be the largest number in $[0, \frac{1}{4}\pi]$ such that

$\underline{a} \le \underline{x}_k + \underline{d}(\theta) \le \underline{b}$, $0 \le \theta \le \theta_B$, holds, and let $\theta_Q$ be the greatest value of $\theta$ in $[0, \frac{1}{4}\pi]$ such that $Q_k(\underline{x}_k + d(\theta))$, $0 \le \theta \le \theta_Q$, decreases monotonically. These numbers are found approximately, $\theta$ is set to the smaller of them, and $\underline{d}$ is overwritten by $\underline{d}(\theta)$. If this change to $\underline{d}$ is restricted by one of the bounds on the variables, the index of that bound is added to $\mathcal{I}$. Alternatively, when $\theta$ is an estimate of $\theta_Q$, termination occurs with $\underline{d}_k$ set to the current $\underline{d}$ if the reduction in $Q_k$ from this move round the trust region boundary is at most the right hand side of expression (3.4). The remaining possibility is another search for a better choice of $\underline{d}$, using the method described already, which begins by testing the termination condition (3.5).

Numerical experiments show that it is very unusual for subroutine TRSBOX to make more than ten changes to $\underline{d}$ when seeking an approximate solution to the subproblem (1.8), even if there are hundreds of variables. Further, the work of each change is only $\mathcal{O}(n)$, except for the task of multiplying $\underline{s}$ by $\nabla^2 Q_k$ whenever a change to $\underline{d}$ is under consideration. Thus the calculation of $\underline{d}_k$ by TRSBOX is within the target of $\mathcal{O}(n^2)$ operations per iteration.

A major difference between a "trust region" and an "alternative" iteration is that, in the latter case, the selection of $t$ for formula (1.6) is made before $\underline{d}_k$ is chosen. Specifically, $t$ is set to an integer in $[1, m]$ that satisfies the equation

$$\|\underline{y}_t - \underline{x}_k\| \;=\; \max\{\|\underline{y}_j - \underline{x}_k\| : j = 1, 2, \ldots, m\}, \tag{3.7}$$

which is helpful to the aim of clustering the interpolation points round $\underline{x}_k$ as the calculation proceeds, but the quadratic model $Q_k(\underline{x})$, $\underline{x} \in \mathcal{R}^n$, is ignored in the construction of $\underline{d}_k$ by an "alternative" iteration. Instead, attention is given to the updating of the inverse matrix $H$ of expression (2.7), the dependence on the interpolation points being through the definitions (2.8) and (2.9) of the submatrices $A$ and $Y$. Details are given in the next section, but a few of them are needed now. The key remark is that, assuming exact arithmetic, the change (1.6) causes the new conditions (1.5) to be linearly dependent (and probably contradictory) if and only if a division by zero occurs in the procedure for updating $H$.

Let $\Lambda_t(\underline{x})$, $\underline{x} \in \mathcal{R}^n$, be a quadratic function that satisfies the Lagrange interpolation conditions

$$\Lambda_t(\underline{y}_j) \;=\; \delta_{jt}, \qquad j = 1, 2, \ldots, m, \tag{3.8}$$

where $\delta_{jt}$ is the Kronecker delta, and let the freedom in $\Lambda_t$ be taken up by minimizing the Frobenius norm of the symmetric second derivative matrix $\nabla^2 \Lambda_t$. It is explained in the next section that the coefficients of $\Lambda_t$ are available in the $t$-th column of the inverse matrix $H$. The function $\Lambda_t$ is relevant, because the only denominator in the updating of $H$ is the expression

$$\sigma \;=\; H_{tt}\, \beta(\underline{x}_k + \underline{d}_k) + \{\Lambda_t(\underline{x}_k + \underline{d}_k)\}^2 \tag{3.9}$$

(Powell, 2006), where $\beta(\underline{x})$, $\underline{x} \in \mathcal{R}^n$, is a nonnegative quartic polynomial that satisfies $\beta(\underline{y}_j) = 0$, $j = 1, 2, \ldots, m$. Further, it is shown in the next section that $\beta$ has the property

$$0 \;\le\; \beta(\underline{x}_k + \alpha\, \{\underline{y}_j - \underline{x}_k\}) \;\le\; \tfrac{1}{2}\, \alpha^2\, (1-\alpha)^2\, \|\underline{y}_j - \underline{x}_k\|^4, \qquad \alpha \in \mathcal{R}, \tag{3.10}$$

for every interpolation point $\underline{y}_j$ that is different from $\underline{x}_k$. The diagonal matrix element $H_{tt}$ is also nonnegative, due to the factorization (1.9) of the leading $m{\times}m$ submatrix of $H$.

The denominator (3.9) is required to be substantial, and in theory is bounded below by $\{\Lambda_t(\underline{x}_k{+}\underline{d}_k)\}^2$. Therefore, on each "alternative" iteration of NEWUOA (Powell, 2006), $\underline{d}_k$ is set to an estimate of the vector $\underline{d}$ that maximizes $|\Lambda_t(\underline{x}_k{+}\underline{d})|$ subject to $\|\underline{d}\|\leq\Delta_k$. It is reported by Powell (2008), however, that some further constraints on $\underline{d}$ not only assist the calculation of $\underline{d}_k$ but also reduce $\#F$ in several experiments. Therefore, on the "alternative" iterations of BOBYQA, $\underline{x}_k+\underline{d}_k$ is selected usually from one of the $m-1$ straight lines in $\mathcal{R}^n$ through $\underline{x}_k$ and another interpolation point. Occasionally, this usual choice of $\underline{d}_k$ is replaced by a constrained Cauchy step of the function $|\Lambda_t(\underline{x}_k{+}\underline{d})|$, $\|\underline{d}\|\leq\Delta_k$, as explained later.

For every integer $j$ in $[1,m]$ such that $\underline{y}_j\neq\underline{x}_k$, let $\phi_j$ be the quadratic $\phi_j(\alpha)=\Lambda_t(\underline{x}_k{+}\alpha\{\underline{y}_j-\underline{x}_k\})$, $\alpha\in\mathcal{R}$, and let $\alpha_j$ be the value of $\alpha$ that maximizes $|\phi_j(\alpha)|$ subject to $\underline{a}\leq\underline{x}_k{+}\alpha(\underline{y}_j-\underline{x}_k)\leq\underline{b}$ and $\|\underline{x}_k{+}\alpha(\underline{y}_j-\underline{x}_k)\|\leq\Delta_k$. Further, from among these integers $j$, let $\ell$ be the one that maximizes the product

$$\{\phi_j(\alpha_j)\}^2\,[\,\tfrac{1}{2}\,H_{tt}\,\alpha_j^2\,(1-\alpha_j)^2\,\|\underline{y}_j-\,\underline{x}_k\|^4+\{\phi_j(\alpha_j)\}^2\,]\,. \tag{3.11}$$

The usual choice of $\underline{d}_k$ is $\alpha_\ell(\underline{y}_\ell-\underline{x}_k)$. Thus the denominator (3.9) is made substantial in a way that employs the bounds (3.10) on $\beta(\underline{x}_k{+}\alpha\{\underline{y}_j-\underline{x}_k\})$, instead of calculating $\beta(\underline{x}_k{+}\alpha\{\underline{y}_j-\underline{x}_k\})$ explicitly, because that would be too expensive for every $j$. The gradient $\underline{\nabla}\Lambda_t(\underline{x}_k)$ is formed in $\mathcal{O}(mn)$ operations before the cycle through $j$, in order that each quadratic function $\phi_j$ can be generated easily from the data $\phi_j(0)=0$, $\phi_j'(0)=(\underline{y}_j-\underline{x}_k)^T\underline{\nabla}\Lambda_k(\underline{x}_k)$ and $\phi_j(1)=\delta_{jt}$. Thus the total work of this part of an "alternative" iteration of BOBYQA is only $\mathcal{O}(mn)$.

There is a fundamental disadvantage, however, in choosing $\underline{d}_k$ to be a multiple of $\underline{y}_\ell-\underline{x}_k$. It is that, if the steps $\underline{y}_j-\underline{x}_k$, $j=1,2,\ldots,m$, fail to span $\mathcal{R}^n$, then, due to formula (1.6), this property is inherited by the new steps $\underline{\widehat{y}}_j-\underline{x}_k$, $j=1,2,\ldots,m$. Furthermore, a tendency towards this hypothetical property occurs in the usual situation when, for sufficiently large $k$, some of the bounds (1.1) are active on every "trust region" iteration. An important task of the "alternative" iterations is to pick steps that oppose this tendency, because the interpolation conditions (1.2) should supply useful estimates of $\underline{s}^T\underline{\nabla}F(\underline{x}_k)$ along all directions $\underline{s}$ in $\mathcal{R}^n$. Such directional derivatives are crucial to the decision whether or not to move away from the boundary of an active constraint. Let $\underline{c}_k$ be the Cauchy step of the maximization of $|\Lambda_k(\underline{x}_k{+}\underline{d})|$, $\underline{d}\in\mathcal{R}^n$, subject to the usual constraints, details being given below. The condition

$$\{\Lambda_t(\underline{x}_k{+}\underline{c}_k)\}^2\;>\;H_{tt}\,\beta(\underline{x}_k{+}\,\underline{d}_k)+\{\Lambda_t(\underline{x}_k{+}\,\underline{d}_k)\}^2 \tag{3.12}$$

is going to be tested for the choice of $\underline{d}_k$ in the previous paragraph. If it is satisfied, then $\underline{d}_k$ is replaced by $\underline{c}_k$, in order to increase the denominator in the procedure for updating the inverse matrix $H$, and in order to resist the possible tendencies towards degeneracy that have been mentioned.

Two Cauchy steps are generated, one being for the minimization of $\Lambda_t(\underline{x}_k+\underline{s})$, $\|\underline{s}\| \leq \Delta_k$, and the other one being for the minimization of $-\Lambda_t(\underline{x}_k+\underline{s})$, $\|\underline{s}\| \leq \Delta_k$, with the usual bounds $\underline{a} \leq \underline{x}_k+\underline{s} \leq \underline{b}$. We pick the Cauchy step that provides the larger value of $|\Lambda_t(\underline{x}_k+\underline{c}_k)|$. Only the first of these calculations is described below, the other one being similar. First the procedure of the next paragraph provides the exact solution, $\underline{s}=\underline{s}_k$ say, of the subproblem

$$\left.\begin{array}{lll} \text{Minimize} & \Lambda_t(\underline{x}_k) + \underline{s}^T \underline{\nabla} \Lambda_t(\underline{x}_k), & \underline{s} \in \mathcal{R}^n, \\ \text{subject to} & \|\underline{s}\| \leq \Delta_k \quad \text{and} & \underline{a} \leq \underline{x}_k + \underline{s} \leq \underline{b} \end{array}\right\}. \tag{3.13}$$

Then $\underline{c}_k$ is set to the multiple of $\underline{s}_k$ that minimizes $\Lambda_t(\underline{x}_k+\underline{c}_k)$ subject to $\|\underline{c}_k\| \leq \Delta_k$ and $\underline{a} \leq \underline{x}_k+\underline{c}_k \leq \underline{b}$. We call this technique a "Cauchy step", because the objective function of expression (3.13) is a linear approximation to $\Lambda_t(\underline{x}_k+\underline{s})$, $\underline{s} \in \mathcal{R}^n$, but the construction of $\underline{c}_k$ from $\underline{s}_k$ requires the curvature term $\underline{s}_k^T \nabla^2 \Lambda_t(\underline{x}_k) \underline{s}_k$, which is generated in $\mathcal{O}(mn)$ operations.

Let $\underline{g}$ be the gradient $\underline{\nabla} \Lambda_t(\underline{x}_k)$ throughout this paragraph, and let $\widehat{\underline{s}}$ be the vector with the components

$$\widehat{s}_i = \left\{ \begin{array}{cc} a_i - (\underline{x}_k)_i, & g_i > 0, \\ 0, & g_i = 0, \\ b_i - (\underline{x}_k)_i, & g_i < 0, \end{array} \right. \qquad i=1,2,\ldots,n. \tag{3.14}$$

If $\|\widehat{\underline{s}}\| \leq \Delta_k$ holds, then $\underline{s} = \widehat{\underline{s}}$ is the required solution of the linear programming subproblem (3.13). Otherwise, there is a subset $\mathcal{S}$ of the integers $\{1,2,\ldots,n\}$ and a multiplier $\mu > 0$ such that the required $\underline{s}$ has the components

$$s_i = \left\{ \begin{array}{cc} \widehat{s}_i, & i \in \mathcal{S}, \\ -\mu\, g_i, & i \notin \mathcal{S}, \end{array} \right. \qquad i=1,2,\ldots,n. \tag{3.15}$$

Further, when $\mathcal{S}$ becomes the required subset, then $\mu$ is defined by expression (3.15) and by the property $\|\underline{s}\| = \Delta_k$. BOBYQA constructs $\mathcal{S}$ by an iterative procedure that begins with $\mathcal{S} = \{i : \widehat{s}_i = 0\}$. Each iteration adds at least one element to $\mathcal{S}$ until termination. For each $\mathcal{S}$ that occurs, a positive value of $\mu$ is given by the equations (3.15) and by $\|\underline{s}\| = \Delta_k$. The calculation is complete if the components (3.15) satisfy the conditions $a_i \leq s_i + (\underline{x}_k)_i \leq b_i$, $i \notin \mathcal{S}$. Otherwise, all the integers $i \notin \mathcal{S}$ of failures of these conditions are added to $\mathcal{S}$ and a new iteration is begun. We note that at most $n-1$ iterations are required, that the work of each iteration is only $\mathcal{O}(n)$, and that the calculated values of $\mu$ increase strictly monotonically.

By employing the techniques in the second half of this section, subroutine ALTMOV of BOBYQA constructs both $\underline{d}_k$ and $\underline{c}_k$ on every "alternative" iteration. The decision whether to overwrite $\underline{d}_k$ by $\underline{c}_k$ is taken later, after the term $\beta(\underline{x}_k+\underline{d}_k)$ of the test (3.12) is calculated within the updating procedure of Section 4.

## 4. Updating procedures

Much of the material of this section can be found in several papers by the author, including Powell (2006). The bounds (1.1) are irrelevant, because we address the problem of calculating the new quadratic model $Q_{k+1}$ from $Q_k$, when $Q_{k+1}$ has to satisfy the interpolation conditions (1.5), and when the remaining freedom in $Q_{k+1}$ is taken up by requiring $\nabla^2 Q_{k+1}$ to be the symmetric matrix that minimizes the Frobenius norm $\|\nabla^2 Q_{k+1} - \nabla^2 Q_k\|_F$, the matrix $\nabla^2 Q_k$ being symmetric. This problem is expressed below as the solution of a linear system of equations that has the partitioned form (2.6).

The KKT conditions of the calculation of $Q_{k+1}$ provide a property that is highly useful when the number $m$ of interpolation conditions is much less than $\frac{1}{2}n^2$. It is that the change to the second derivative matrix of the model can be expressed as the sum

$$\nabla^2 Q_{k+1} - \nabla^2 Q_k \;=\; \textstyle\sum_{\ell=1}^m \lambda_\ell \, \underline{\widehat{y}}_\ell \, \underline{\widehat{y}}_\ell^T, \tag{4.1}$$

where the multipliers $\lambda_\ell$, $\ell = 1, 2, \ldots, m$, satisfy the equations

$$\textstyle\sum_{\ell=1}^m \lambda_\ell \;=\; 0 \quad \text{and} \quad \textstyle\sum_{\ell=1}^m \lambda_\ell \, \underline{\widehat{y}}_\ell \;=\; 0. \tag{4.2}$$

Let $c \in \mathcal{R}$ and $\underline{g} \in \mathcal{R}^n$ be the differences $Q_{k+1}(\underline{x}_0) - Q_k(\underline{x}_0)$ and $\underline{\nabla} Q_{k+1}(\underline{x}_0) - \underline{\nabla} Q_k(\underline{x}_0)$, respectively, where $\underline{x}_0$ is the current position of the origin, which is shifted occasionally as mentioned in the second paragraph of Section 3. We write $Q_{k+1}$ in the form

$$Q_{k+1}(\underline{x}) = Q_k(\underline{x}) + c + (\underline{x} - \underline{x}_0)^T \underline{g} + \tfrac{1}{2} \, (\underline{x} - \underline{x}_0)^T \Big\{ \sum_{\ell=1}^m \lambda_\ell \, \underline{\widehat{y}}_\ell \underline{\widehat{y}}_\ell^T \Big\} \, (\underline{x} - \underline{x}_0), \quad \underline{x} \in \mathcal{R}^n. \tag{4.3}$$

Thus the construction of $Q_{k+1}$ is reduced to the calculation of $m+n+1$ unknowns, namely $c \in \mathcal{R}$ and the components of $\underline{g} \in \mathcal{R}^n$ and $\underline{\lambda} \in \mathcal{R}^m$. The values of these unknowns are derived from equations (1.5) and (4.2). Moreover, the conditions (4.2) allow every $\underline{\widehat{y}}_\ell$ to be replaced by $\underline{\widehat{y}}_\ell - \underline{x}_0$ in expressions (4.1)–(4.3).

By making this replacement, and by letting $\underline{x}$ be $\underline{\widehat{y}}_j$ in the form (4.3), we find that the constraints (1.5) on $Q_{k+1}$ are the equations

$$c + (\underline{\widehat{y}}_j - \underline{x}_0)^T \underline{g} + \tfrac{1}{2} \sum_{\ell=1}^m \lambda_\ell \, \{ (\underline{\widehat{y}}_j - \underline{x}_0)^T (\underline{\widehat{y}}_\ell - \underline{x}_0) \}^2 \;=\; F(\underline{\widehat{y}}_j) - Q_k(\underline{\widehat{y}}_j), \quad j = 1, 2, \ldots, m. \tag{4.4}$$

It follows that, if we put hats on all the $\underline{y}$ vectors in the definitions (2.8) and (2.9) of $A$ and $Y$, and if we let $\underline{r} \in \mathcal{R}^m$ have the components $F(\underline{\widehat{y}}_j) - Q_k(\underline{\widehat{y}}_j)$, $j = 1, 2, \ldots, m$, then we have derived the first $m$ rows of the partitioned system (2.6). Furthermore, with this modification of $Y$, the conditions (4.2) supply the other $n+1$ rows of the partitioned system (2.6).

When the partitioned matrix $H$ in expression (2.7) is the inverse of the matrix of the linear system of the previous paragraph, then the solution of the system

is $H$ times the right hand side. Thus the coefficients $\lambda_\ell$, $\ell=1,2,\ldots,m$, of the form (4.3) are the components of $\Omega\,\underline{r}$, the coefficient $c$ is the first component of $\Xi\,\underline{r}\in\mathcal{R}^{n+1}$, and the components of $\underline{g}\in\mathcal{R}^n$ are the last $n$ components of $\Xi\,\underline{r}$. A further simplification comes from the observation that equations (1.6) and (1.2) imply the property

$$r_j = F(\underline{\widehat{y}}_j) - Q_k(\underline{\widehat{y}}_j) = F(\underline{y}_j) - Q_k(\underline{y}_j) = 0, \qquad j\in\{1,2,\ldots,m\}\backslash\{t\}. \tag{4.5}$$

Therefore $\Omega\,\underline{r}$ and $\Xi\,\underline{r}$ are multiples of the $t$-th column of $\Omega$ and $\Xi$, respectively, the multiplying factor being $F(\underline{\widehat{y}}_t)-Q_k(\underline{\widehat{y}}_t)$ in both cases. These remarks provide BOBYQA with the vectors $\underline{g}$ and $\underline{\lambda}$ for the next quadratic model (4.3), but the coefficient $c$ is not required.

A disadvantage of the form (4.3), however, is that, if $\nabla^2 Q_k$ is available, and if $\nabla^2 Q_{k+1}$ is stored explicitly, then the calculation of all its elements would take $\mathcal{O}(mn^2)$ operations. Instead, the work of a typical iteration of BOBYQA is kept within $\mathcal{O}(m^2)$ operations by writing second derivative matrices of quadratic models in the form

$$\nabla^2 Q \;=\; M + \textstyle\sum_{\ell=1}^m \mu_\ell\,(\underline{y}_\ell - \underline{x}_0)(\underline{y}_\ell - \underline{x}_0)^T. \tag{4.6}$$

A symmetric $n{\times}n$ matrix $M$, with the parameters $\mu_\ell$ that specify $\nabla^2 Q_k$, is known at the beginning of the $k$-th iteration. After choosing the integer $t$ of formula (1.6), the term $\mu_t(\underline{y}_t-\underline{x}_0)(\underline{y}_t-\underline{x}_0)^T$ of expression (4.6) is added to $M$ explicitly and $\mu_t$ is set to zero. It follows from equations (4.3) and (1.6) that the construction of $\nabla^2 Q_{k+1}$ from $\nabla^2 Q_k$ is completed by replacing $\mu_\ell$ by $\mu_\ell+\lambda_\ell$ for $\ell=1,2,\ldots,m$, no further change being made to $M$. Because the representation (4.6) allows any vector in $\mathcal{R}^n$ to be multiplied by $\nabla^2 Q$ in $\mathcal{O}(mn)$ operations, it is suitable for every calculation of $\underline{d}_k$ in Section 3.

A gradient of $Q_k$ is also required at the beginning of the $k$-th iteration. We let it be $\underline{g}_k=\underline{\nabla} Q_k(\underline{x}_k)$, because of the importance of $\underline{g}_k$ to the construction of $\underline{d}_k$ on a "trust region" iteration. This choice is also helpful to the preservation of accuracy in floating point arithmetic in the usual case when $\underline{g}_k$ becomes very small as $k$ increases. Therefore BOBYQA employs the form

$$Q_k(\underline{x}) = Q_k(\underline{x}_k) + (\underline{x}-\underline{x}_k)^T\underline{g}_k + \tfrac{1}{2}\,(\underline{x}-\underline{x}_k)^T\nabla^2 Q_k\,(\underline{x}-\underline{x}_k), \qquad \underline{x}\in\mathcal{R}^n. \tag{4.7}$$

We see that the coefficient $c$ of expression (4.3) is unnecessary, the values $Q_k(\underline{x}_k)= F(\underline{x}_k)$ and $Q_{k+1}(\underline{x}_{k+1})=F(\underline{x}_{k+1})$ being available, because $\underline{x}_k$ and $\underline{x}_{k+1}$ are interpolation points of $Q_k$ and $Q_{k+1}$, respectively. By differentiating the function (4.3), we obtain the formula

$$\underline{\nabla} Q_{k+1}(\underline{x}_k) \;=\; \underline{g}_k + \underline{g} + \sum_{\ell=1}^m \lambda_\ell\,\{(\underline{\widehat{y}}_\ell - \underline{x}_0)^T(\underline{x}_k - \underline{x}_0)\}\,(\underline{\widehat{y}}_\ell - \underline{x}_0). \tag{4.8}$$

It supplies the required gradient $\underline{g}_{k+1}=\underline{\nabla} Q_{k+1}(\underline{x}_{k+1})$ in the case $\underline{x}_{k+1}=\underline{x}_k$, the vectors $\underline{g}\in\mathcal{R}^n$ and $\underline{\lambda}\in\mathcal{R}^m$ being given by the system (2.6) as mentioned already. When formula (1.4) sets $\underline{x}_{k+1}=\underline{x}_k+\underline{d}_k$, however, then $\underline{g}_{k+1}$ is formed by adding

$\nabla^2 Q_{k+1}\,\underline{d}_k$ to $\underline{\nabla} Q_{k+1}(\underline{x}_k)$. The description of the updating of the quadratic model is complete.

At the beginning of the $k$-th iteration, the matrix $H = W^{-1}$ of expression (2.7) is known, with $\Omega$ in the factored form (1.9), the submatrices $A$ and $Y$ of $W$ being defined by equations (2.8) and (2.9). The points $\underline{y}_j$, $j=1,2,\ldots,m$, of these definitions are the interpolation points of the constraints (1.2) on $Q_k$. The construction of $Q_{k+1}$ from $Q_k$, however, is dependent on the new interpolation points $\widehat{\underline{y}}_j$, $j=1,2,\ldots,m$. In other words, it is dependent on the $H$ matrix that is going to be available at the beginning of the $(k+1)$-th iteration. Therefore BOBYQA derives the new $H$ matrix from the old one, details being given below, before applying the procedure in the previous two paragraphs for updating the quadratic model.

For fixed $\underline{x}_0$, let the change (1.6) be made to the interpolation points. We see that all changes to the elements (2.8) are confined to the $t$-th row and column of $A$, and that only the $t$-th column of $Y$ is altered in definition (2.9). Thus the changes to $W$ in expression (2.7) are also confined to its $t$-th row and column. Hence, assuming nonsingularity, the new $H$ matrix, $H_{\rm new}$ say, can be derived from the old one, $H$ say, and from the new $t$-th column of $W$, the new $t$-th row of $W$ being given by symmetry. Indeed, letting $W_{\rm old}$ be the inverse of $H$, we define $W_{\rm new}$ by overwriting the $t$-th column and row of $W_{\rm old}$ by the new ones, which implies $H_{\rm new} = W_{\rm new}^{-1}$. The calculation of $H_{\rm new}$ by BOBYQA is a version of this procedure, without the explicit calculation of $W_{\rm old}$ and $W_{\rm new}$, that takes only $\mathcal{O}(m^2)$ computer operations.

We find in Powell (2006) that $H_{\rm new}$ is given by the formula

$$\begin{aligned} H_{\rm new} \;=\; & H + \sigma^{-1} \Big[ \alpha\, (\underline{e}_t - H\,\underline{w})\, (\underline{e}_t - H\,\underline{w})^T - \beta\, H\,\underline{e}_t\, \underline{e}_t^T H \\ & \qquad + \tau \left\{ H\,\underline{e}_t\, (\underline{e}_t - H\,\underline{w})^T + (\underline{e}_t - H\,\underline{w})\, \underline{e}_t^T H \right\} \Big], \end{aligned} \qquad (4.9)$$

where $\underline{e}_t$ is the $t$-th coordinate vector in $\mathcal{R}^{m+n+1}$, where $\underline{w}$ has the components

$$\left. \begin{array}{ll} w_i \;=\; \frac{1}{2} \{ (\underline{y}_i - \underline{x}_0)^T (\underline{x}^+ - \underline{x}_0) \}^2, & i=1,2,\ldots,m \\ w_{m+1} = 1 \quad \text{and} \quad w_{i+m+1} = (\underline{x}^+ - \underline{x}_0)_i, & i=1,2,\ldots,n \end{array} \right\}, \qquad (4.10)$$

$\underline{x}^+$ being the vector $\underline{x}_k + \underline{d}_k$, and where the parameters take the values

$$\left. \begin{array}{ll} \alpha = \underline{e}_t^T H\, \underline{e}_t, & \beta = \frac{1}{2}\, \| \underline{x}^+ - \underline{x}_0 \|^4 - \underline{w}^T H\, \underline{w}, \\ \tau = \underline{e}_t^T H\, \underline{w} & \text{and} \quad \sigma = \alpha\, \beta + \tau^2. \end{array} \right\} \qquad (4.11)$$

There is another twist in the updating techniques of NEWUOA and BOBYQA, which is that the $(m+1)$-th column and row of each $H$ matrix are not retained. The absence of these elements does not matter in the updating of quadratic models, because the $(m+1)$-th component of the right hand side of the system (2.6) is zero, and because that updating does not require the system (2.6) to supply the value of $c$ for expression (4.3). Formula (4.9) is modified slightly below, however,

because $H\underline{w}$ includes a substantial contribution from the $(m{+}1)$-th column of $H$, due to $w_{m+1}=1$.

Let $s$ be the integer in $[1,m]$ such that $\underline{x}_k=\underline{y}_s$, and let $\underline{v}$ be the $s$-th column of $W=H^{-1}$, so it has the components

$$\left.\begin{array}{ll} v_i \;=\; \frac{1}{2}\,\{(\underline{y}_i-\underline{x}_0)^T(\underline{x}_k-\underline{x}_0)\}^2, & i=1,2,\ldots,m \\ v_{m+1} = 1 \quad \text{and} \quad v_{i+m+1} = (\underline{x}_k-\underline{x}_0)_i, & i=1,2,\ldots,n \end{array}\right\}. \tag{4.12}$$

We recall from Section 1 that $s$ is different from $t$. The equations $W=H^{-1}$ and $W\underline{e}_s=\underline{v}$ imply $H\underline{v}=\underline{e}_s$, giving the identities

$$\left.\begin{array}{rcl} H\underline{w} &=& H(\underline{w}-\underline{v}) + \underline{e}_s \\ \underline{w}^T H\underline{w} &=& (\underline{w}-\underline{v})^T H(\underline{w}-\underline{v}) + 2\,w_s - v_s \end{array}\right\}. \tag{4.13}$$

The modification of formula (4.9) is that the four occurrences of $\underline{e}_t - H\underline{w}$ are replaced by $\underline{e}_t-\underline{e}_s-H(\underline{w}-\underline{v})$. We also replace $\underline{e}_t^T H\underline{w}$ and $\underline{w}^T H\underline{w}$ by $\underline{e}_t^T H(\underline{w}-\underline{v})$ and by the second part of expression (4.13) in the definitions (4.11) of $\tau$ and $\beta$. Because the $(m{+}1)$-th component of $\underline{w}-\underline{v}$ is zero, it follows that the new formula provides the first $m$ and last $n$ columns and rows of $H_{\text{new}}$ as required when the $(m{+}1)$-th row and column of $H$ are not available.

This version of formula (4.9) is applied directly to update the $(m+n)\times n$ matrix that is called BMAT in the Fortran listing of BOBYQA. The first $m$ rows of BMAT are $\Xi^T$ without its first column, and the last $n$ rows of BMAT are $\Upsilon$ without its first row and column, the submatrices $\Xi^T$ and $\Upsilon$ being taken from expression (2.7). Furthermore, the $m\times(m-n-1)$ matrix that is called ZMAT in the Fortran listing is the matrix $Z$ of the factorization $\Omega = ZZ^T$ of the leading $m{\times}m$ submatrix of $H$. Of course $Z$ has to be updated too, so that $ZZ^T$ becomes a factorization of the leading $m{\times}m$ submatrix of $H_{\text{new}}$. There is a choice of updating procedures, due partly to the nonuniqueness of $Z$ mentioned in the penultimate paragraph of Section 3. BOBYQA employs the following one, which is also taken from Powell (2006).

The procedure begins by postmultiplying $Z$ by an $(m-n-1)\times(m-n-1)$ orthogonal matrix such that, after the multiplication, only the first component of the $t$-th row of $Z$ is nonzero, which preserves the factorization $\Omega = ZZ^T$. The advantage of this form is that the required new $Z$ matrix, $Z_{\text{new}}$ say, can be constructed by changing only the first column of $Z$. Specifically the new first column has the elements

$$(Z_{\text{new}})_{i1} \;=\; \sigma^{-1/2}\,[\,\tau Z_{i1} + (\underline{e}_t - \underline{e}_s - H\{\underline{w}-\underline{v}\})_i\, Z_{t1}\,], \qquad i=1,2\ldots,m, \tag{4.14}$$

the parameters $\sigma$ and $\tau$ and the vector $\underline{e}_t-\underline{e}_s-H(\underline{w}-\underline{v})$ being available from the updating of BMAT. Just one more feature of the updating calculations requires attention, namely the action that is taken to preserve $\sigma > 0$ in the presence of computer rounding errors. It is the subject of Section 5.

We complete this section by attending to the questions that are left open in the paragraph that includes expressions (3.8)–(3.10). Identifying a usable form of

the Lagrange function $\Lambda_t(\underline{x})$, $\underline{x}\in\mathcal{R}^n$, that satisfies the conditions (3.8) is closely related to the construction of $Q_{k+1}-Q_k$ at the beginning of this section, because in both cases, after satisfying interpolation conditions, the freedom in the required quadratic is taken up by minimizing the Frobenius norm of its symmetric second derivative matrix. Thus the parameters of the quadratics are defined by linear systems of the form (2.6), and in both cases $\underline{r}$ is a multiple of the coordinate vector $\underline{e}_t\in\mathcal{R}^m$. A difference, however, is that the interpolation points of $Q_{k+1}-Q_k$ and of $\Lambda_t$ are $\{\underline{\widehat{y}}_j : j=1,2,\ldots,m\}$ and $\{\underline{y}_j : j=1,2,\ldots,m\}$, respectively. Therefore the parameters of $Q_{k+1}-Q_k$ are elements of the $t$-th column of $H_{\rm new}$ multiplied by $F(\underline{\widehat{y}}_t)-Q_k(\underline{\widehat{y}}_t)$, as mentioned after equation (4.5), but the corresponding parameters of $\Lambda_t$ are elements of the $t$-th column of the $H$ matrix at the beginning of the $k$-th iteration.

Specifically, using the form (2.7) of this $H$ matrix, we now let $\lambda_\ell$, $\ell=1,2,\ldots,m$, be the components of $\Omega\,\underline{e}_t$, we let $c$ be the first component of $\Xi\,\underline{e}_t\in\mathcal{R}^{n+1}$, and we let the components of $\underline{g}\in\mathcal{R}^n$ be the other components of $\Xi\,\underline{e}_t$. It follows that $\Lambda_t$ is the function

$$\Lambda_t(\underline{x}) \;=\; c+(\underline{x}-\underline{x}_0)^T\underline{g}+\tfrac{1}{2}\,(\underline{x}-\underline{x}_0)^T\nabla^2\Lambda_t\,(\underline{x}-\underline{x}_0), \qquad \underline{x}\in\mathcal{R}^n, \tag{4.15}$$

with the second derivative matrix

$$\nabla^2\Lambda_t \;=\; \textstyle\sum_{\ell=1}^m \lambda_\ell\,(\underline{y}_\ell-\underline{x}_0)\,(\underline{y}_\ell-\underline{x}_0)^T, \tag{4.16}$$

whose elements are not found explicitly because that would be too expensive. We recall that $\underline{x}_k$ is an interpolation point different from $\underline{y}_t$, which provides $\Lambda_t(\underline{x}_k)=0$. Therefore, partly because $c$ is not available, we work with the form

$$\Lambda_t(\underline{x}) \;=\; (\underline{x}-\underline{x}_k)^T\underline{\nabla}\Lambda_t(\underline{x}_k)+\tfrac{1}{2}\,(\underline{x}-\underline{x}_k)^T\nabla^2\Lambda_t\,(\underline{x}-\underline{x}_k), \qquad \underline{x}\in\mathcal{R}^n, \tag{4.17}$$

after calculating $\underline{\nabla}\Lambda_t(\underline{x}_k)=\underline{g}+\nabla^2\Lambda_t\,(\underline{x}_k-\underline{x}_0)$ explicitly in $\mathcal{O}(mn)$ operations, as mentioned in Section 3.

Next we address the claim that expression (3.9) is the denominator of the updating formula (4.9) in the case $\underline{x}^+=\underline{x}_k+\underline{d}_k$. Because the parameters $\underline{\lambda}\in\mathcal{R}^m$, $c\in\mathcal{R}$ and $\underline{g}\in\mathcal{R}^n$ of $\Lambda_t$ are the elements of $H\underline{e}_t$, we can employ the notation

$$\begin{aligned}\tau \;=\; \underline{e}_t^T H\,\underline{w} \;&=\; \textstyle\sum_{\ell=1}^m \lambda_\ell\,w_\ell + c\,w_{m+1} + \sum_{i=1}^n w_{i+m+1}\,g_i \\ &=\; \tfrac{1}{2}\,(\underline{x}^+-\underline{x}_0)^T\nabla^2\Lambda_t\,(\underline{x}^+-\underline{x}_0)+c+(\underline{x}^+-\underline{x}_0)^T\underline{g},\end{aligned} \tag{4.18}$$

the last line being a conseqence of equations (4.10) and (4.16). Therefore expression (4.15) shows that $\tau$ is the function value $\tau=\Lambda_t(\underline{x}^+)=\Lambda_t(\underline{x}_k+\underline{d}_k)$. Moreover, $\alpha=\underline{e}_t^T H\underline{e}_t=H_{tt}$ holds because $\underline{e}_t$ is the $t$-th coordinate vector in $\mathcal{R}^{m+n+1}$, and we regard the equation

$$\beta(\underline{x}^+) \;=\; \tfrac{1}{2}\,\|\underline{x}^+-\underline{x}_0\|^4-\underline{w}^T H\,\underline{w}, \qquad \underline{x}^+\in\mathcal{R}^n, \tag{4.19}$$

where $\underline{w}$ has the components (4.10), as the definition of $\beta=\beta(\underline{x}^+)=\beta(\underline{x}_k+\underline{d}_k)$. Thus the values (3.9) and (4.11) of the denominator $\sigma$ are the same.

It remains to justify the bounds (3.10) on $\beta$. It is helpful to consider the dependence of the parameters (4.11) on the occasional shift of origin $\underline{x}_0$. Second derivatives of quadratic functions, for example the matrix (4.16), do not depend on the position of the origin, which is the reason for the last $n+1$ equations of the system (2.6). In theory, therefore, the $Z$ matrices of BOBYQA have this property too, including the updating formula (4.14). It follows that all the parameters (4.11) are also independent of $\underline{x}_0$. In particular, when investigating $\beta(\underline{x}^+)$, we may assume $\underline{x}_0 = \underline{x}^+$ in the definition (4.19), and then the components (4.10) supply the coordinate vector $\underline{w} = \underline{e}_{m+1} \in \mathcal{R}^{m+n+1}$. Further, the definition (4.19) provides $\beta(\underline{x}^+) = -H_{m+1\,m+1} = -\Upsilon_{11}$. The symmetric matrix $\Upsilon$ of expression (2.7) has no positive eigenvalues, because $W$ is nonsingular and because the elements (2.8) imply that the symmetric submatrix $A$ is positive definite or semi-definite. Thus the lower bound $\beta(\underline{x}^+) \geq 0$ is established in Powell (2004) for every choice of $\underline{x}^+ \in \mathcal{R}^n$.

In the upper bound (3.10) on $\beta(\underline{x}^+)$, however, $\underline{x}^+$ is on the straight line through the interpolation points $\underline{x}_k$ and $\underline{y}_j$. We continue to let $s$ be the integer in $[1, n]$ such that $\underline{x}_k = \underline{y}_s$, we assume $j \neq s$ in the equation

$$\underline{x}^+ \;=\; \underline{x}_k + \alpha\,\{\underline{y}_j - \underline{x}_k\} \;=\; \underline{y}_s + \alpha\,\{\underline{y}_j - \underline{y}_s\}, \qquad \alpha \in \mathcal{R}, \tag{4.20}$$

and we simplify the following analysis by assuming without loss of generality that, instead of $\underline{x}_0 = \underline{x}^+$, the origin has been shifted to $\underline{x}_0 = \underline{x}_k$. It follows from the definitions (2.8) and (2.9) that the vector (4.10) has the components

$$\left.\begin{array}{ll} w_i \;=\; \alpha^2 A_{ij}, & i=1,2,\ldots,m \\ w_{m+1} = 1 \quad \text{and} \quad w_{i+m+1} = \alpha\, Y_{i+1\,j}, & i=1,2,\ldots,n \end{array}\right\}, \tag{4.21}$$

which are required for the $\underline{w}^T H \underline{w}$ term of expression (4.19). Now the choice $\underline{x}_0 = \underline{x}_k = \underline{y}_s$ provides the $W$ matrix (2.7) with the property $W \underline{e}_s = \underline{e}_{m+1}$, so $H = W^{-1}$ satisfies $H \underline{e}_{m+1} = \underline{e}_s$. Therefore, in the contribution from $w_{m+1}$ to $\underline{w}^T H \underline{w}$, $w_{m+1}$ is multiplied by $w_s$. The choice $\underline{x}_0 = \underline{x}_s$, however, also provides $w_s = \alpha^2 A_{sj} = 0$. Therefore $\underline{w}^T H \underline{w} = \underline{u}^T H \underline{u}$ holds, where we let $\underline{u}$ have the same components as $\underline{w}$ except for $u_{m+1} = \alpha$.

It is helpful that the first $m$ and last $n+1$ components of $\underline{u} \in \mathcal{R}^{m+n+1}$ are those of the vectors $\alpha^2 A\,\underline{e}_j \in \mathcal{R}^m$ and $\alpha\, Y \underline{e}_j \in \mathcal{R}^{n+1}$. Indeed, the partition (2.10) gives the equation

$$\begin{aligned} \underline{w}^T H \underline{w} \;&=\; \underline{u}^T H \underline{u} \\ &=\; \alpha^4\, \underline{e}_j^T A\, \Omega\, A\, \underline{e}_j \;+\; 2\,\alpha^3\, \underline{e}_j^T A\, \Xi^T\, Y \underline{e}_j \;+\; \alpha^2\, \underline{e}_j^T\, Y^T\, \Upsilon\, Y \underline{e}_j. \end{aligned} \tag{4.22}$$

By employing the product $W H = I$ of the partitioned matrices (2.7), we find the relations $Y^T \Upsilon = -A\, \Xi^T$ and $\Xi^T Y = I - \Omega\, A$. Thus we eliminate $\Upsilon$ and then $\Xi^T$ from expression (4.22), the result being the formula

$$\underline{w}^T H \underline{w} \;=\; (\alpha^4 - 2\,\alpha^3 + \alpha^2)\, \underline{e}_j^T A\, \Omega\, A\, \underline{e}_j \;+\; (2\,\alpha^3 - \alpha^2)\, \underline{e}_j^T A\, \underline{e}_j. \tag{4.23}$$

Moreover, the definitions (2.8) and (4.20) with $\underline{x}_0 = \underline{x}_k$ yield the diagonal elements $\underline{e}_j^T A \, \underline{e}_j = \frac{1}{2} \, \|\underline{y}_j - \underline{x}_k\|^4$, $j = 1, 2, \ldots, m$, and the relation $\|\underline{x}^+ - \underline{x}_0\|^4 = \alpha^4 \, \|\underline{y}_j - \underline{x}_k\|^4$. It follows from equations (4.19) and (4.23) that $\beta$ does satisfy the required bound

$$\begin{aligned} \beta(\underline{x}^+) &= \tfrac{1}{2} \, \alpha^4 \, \|\underline{y}_j - \underline{x}_k\|^4 - \{ \alpha^2 (1 - \alpha^2) \, \underline{e}_j^T A \, \Omega \, A \, \underline{e}_j + (\alpha^3 - \tfrac{1}{2} \, \alpha^2) \, \|\underline{y}_j - \underline{x}_k\|^4 \} \\ &= \alpha^2 \, (1 - \alpha)^2 \, \{ \tfrac{1}{2} \, \|\underline{y}_j - \underline{x}_k\|^4 - \underline{e}_j^T A \, \Omega \, A \, \underline{e}_j \} \\ &\le \tfrac{1}{2} \, \alpha^2 \, (1 - \alpha)^2 \, \|\underline{y}_j - \underline{x}_k\|^4, \qquad \alpha \in \mathcal{R}, \end{aligned} \qquad (4.24)$$

the last line being due to the positive semi-definiteness of $\Omega = Z Z^T$.

## 5. The method of RESCUE

Computer rounding errors cause severe damage occasionally to the parameters (4.11) of formula (4.9), large reductions in $|\sigma|$ due to errors being very unwelcome. Further, a negative value of $\sigma$ would exclude the use of equation (4.14) for updating the first column of $Z$. Therefore, on the "trust region" iterations of BOBYQA, the freedom in the choice of $t$ helps to keep $|\tau| = |\underline{e}_t^T H \underline{w}|$ away from zero, details being given in Section 6. Moreover, we recall from Section 3 that the choice of the step $\underline{d}_k$ on an "alternative" iteration is designed to promote a relatively large value of $|\tau| = |\underline{e}_t^T H \underline{w}| = |\Lambda_t(\underline{x}_k + \underline{d}_k)|$. Nevertheless, the question is asked on every iteration of BOBYQA whether or not the calculated denominator $\sigma = \alpha\beta + \tau^2$ seems to be adequate.

The value of $\alpha$ is guaranteed to be nonnegative in practice by employing the equation

$$\alpha \;=\; H_{tt} \;=\; \underline{e}_t^T \Omega \, \underline{e}_t \;=\; \underline{e}_t^T Z \, Z^T \underline{e}_t \;=\; \|Z^T \underline{e}_t\|^2, \qquad (5.1)$$

where $\underline{e}_t$ is now the $t$-th coordinate vector in $\mathcal{R}^m$, and we recall that $\beta \ge 0$ holds in theory. Negative calculated values of $\beta$ are tolerable, however, provided that $|\alpha\beta|$ is substantially less than $\tau^2$ in the formula $\sigma = \alpha\beta + \tau^2$. They occur often when the number $m$ of interpolation points is at its maximum value $m = \frac{1}{2}(n+1)(n+2)$, because then in theory the function $\beta(\underline{x}^+)$, $\underline{x}^+ \in \mathcal{R}^n$, given in expression (4.19), is identically zero. Further, for general $m$, the bounds (3.10) provide the theoretical property $\beta(\underline{y}_j) = 0$, $j = 1, 2, \ldots, m$.

A strong disadvantage of the calculation of $\beta$ is that it includes the term $\frac{1}{2} \, \|\underline{x}^+ - \underline{x}_0\|^4$, as shown in equations (4.11) and (4.19), although in theory $\beta(\underline{x}^+)$ is independent of $\underline{x}_0$. Therefore the contribution from rounding errors to $\beta(\underline{x}^+)$ can be made arbitrarily large, by allowing the origin $\underline{x}_0$ to be sufficiently far from $\underline{x}^+$. To some extent, the disadvantage has to be tolerated, because shifting the origin is expensive, and rounding errors do not cause serious difficulties in most applications of BOBYQA. The level of tolerance on each iteration is that the updating procedures of Section 4 proceed as usual unless the calculated $\beta$ satisfies the condition

$$\sigma \;=\; \alpha \, \beta + \tau^2 \;\le\; \tfrac{1}{2} \, \tau^2. \qquad (5.2)$$

In the case (5.2), a subroutine that has the name RESCUE is called instead. It tries to provide a better denominator $\sigma$ in the following way.

When RESCUE is called, the current $H$ and $Z$ matrices of expressions (2.7) and (1.9) are rejected. The points $\underline{y}_j$ and the function values $F(\underline{y}_j)$, $j=1,2,\ldots,m$, are retained, however, and $\underline{x}_k$ is still the interpolation point that has the property (1.3). The current quadratic model $Q_k(\underline{x})$, $\underline{x}\in\mathcal{R}^n$, is also retained. Usually a few of the interpolation points are replaced, which requires some new values of $F$, and then $Q_k$ is updated to interpolate the new function values, but sometimes the only change at the return from RESCUE is that ZMAT and BMAT have been recalculated, these matrices being introduced in the complete paragraph between equations (4.13) and (4.14). The first task of RESCUE is to shift the origin to the position $\underline{x}_0=\underline{x}_k$, which is easy because we ignore the matrix $H$ that depends on $\underline{x}_0$. The representation (4.6) of $\nabla^2 Q_k$ is updated, however, in the way that does not alter the parameters $\mu_\ell$, $\ell=1,2,\ldots,m$. Indeed, the rank two matrix

$$\begin{aligned}\textstyle\sum_{\ell=1}^m \mu_\ell\,(\underline{y}_\ell-\underline{x}_0)\,(\underline{y}_\ell-\underline{x}_0)^T-\sum_{\ell=1}^m \mu_\ell\,(\underline{y}_\ell-\underline{x}_k)\,(\underline{y}_\ell-\underline{x}_k)^T\\ =\;(\underline{z}-\textstyle\sum_{\ell=1}^m \mu_\ell\,\underline{y}_\ell)\,(\underline{x}_0-\underline{x}_k)^T+(\underline{x}_0-\underline{x}_k)\,(\underline{z}-\sum_{\ell=1}^m \mu_\ell\,\underline{y}_\ell)^T\end{aligned}\qquad(5.3)$$

is added to $M$ in expression (4.6), where $\underline{z}$ is the vector $\frac{1}{2}\,(\sum_{\ell=1}^m \mu_\ell)\,(\underline{x}_0+\underline{x}_k)$.

Next we compare $\underline{x}_0=\underline{x}_k$ with the initial $\underline{x}_0$ in the formulae (2.2) and (2.3) that provide the interpolation points for the first iteration. A possible way of removing the errors in ZMAT and BMAT that caused the condition (5.2) is to replace the current points $\underline{y}_j$, $j=1,2,\ldots,m$, by $\underline{\gamma}_j$, $j=1,2,\ldots,m$, say, the new points being $\underline{x}_0=\underline{x}_k$ and $m-1$ other vectors that are analogous to the choices (2.2) and (2.3). These new points are crucial in the description of RESCUE. Specifically, after setting $\underline{\gamma}_1=\underline{x}_0=\underline{x}_k$, we let $\alpha_i$ and $\beta_i$ be nonzero multipliers that satisfy $\alpha_i\neq\beta_i$, $i=1,2,\ldots,n$, and we pick the points

$$\underline{\gamma}_{i+1}=\underline{x}_0+\alpha_i\,\underline{e}_i \quad\text{and}\quad \underline{\gamma}_{n+i+1}=\underline{x}_0+\beta_i\,\underline{e}_i,\qquad i=1,2,\ldots,n,\qquad(5.4)$$

as in the form (2.10). In the case $m\leq 2n+1$, the new points are $\underline{\gamma}_j$, $j=1,2,\ldots,m$, but, if $m>2n+1$ holds, the vectors

$$\underline{\gamma}_j\;=\;\underline{\gamma}_{p(j)+1}+\underline{\gamma}_{q(j)+1}-\underline{x}_0,\qquad 2n+2\leq j\leq m,\qquad(5.5)$$

supplement the choices (5.4), the integers $p(j)$ and $q(j)$ being the same as those of equation (2.3). The $Z$ and $H$ matrices that have been thrown away are always replaced by the $Z$ and $H$ matrices of these new points, their elements being given easily and accurately by the techniques of Section 2. We find after the next two paragraphs that the new $Z$ and $H$ matrices are going to be updated.

The choices of $\alpha_i$ and $\beta_i$, $i=1,2,\ldots,n$, for formula (5.4) require more care than the corresponding choices for expression (2.10), because now $\underline{x}_0=\underline{x}_k$ may be arbitrarily close to the boundary of the feasible region $\{\underline{x}:\underline{a}\leq\underline{x}\leq\underline{b}\}$. We pick $\alpha_i=\Delta_k$ and $\beta_i=-\Delta_k$ for all integers $i$ in $[1,n]$ such that both $\underline{x}_k+\Delta_k\underline{e}_i$ and

$\underline{x}_k - \Delta_k \underline{e}_i$ are feasible. Otherwise, $\alpha_i$ is set to $\Delta_k$ or $-\Delta_k$ in the case $(\underline{x}_k)_i + \Delta_k \le b_i$ or $(\underline{x}_k)_i - \Delta_k \ge a_i$, respectively, one of these inequalities being true due to $\Delta_k \le \Delta_1$ and the bounds (2.1). Further, for these troublesome integers $i$, we set $\beta_i$ to $a_i - (\underline{x}_k)_i$ or $b_i - (\underline{x}_k)_i$ in the case $\alpha_i > 0$ or $\alpha_i < 0$, respectively, except that, if this choice has the property $|\beta_i| < \frac{1}{2}\Delta_k$, then $\beta_i$ is replaced by $\frac{1}{2}\alpha_i$. It follows that all the points $\underline{\gamma}_i$, $i = 1, 2, \ldots, m$, are feasible, and that the parameters of expression (5.4) satisfy $|\alpha_i| = \Delta_k$, $\frac{1}{2}\Delta_k \le |\beta_i| \le \Delta_k$ and $|\alpha_i - \beta_i| \ge \frac{1}{2}\Delta_k$, $i = 1, 2, \ldots, n$. Thus the new set of interpolation points is suitable in practice for the method of RESCUE.

The points $\underline{\gamma}_j$, $j = 1, 2, \ldots, m$, have the disadvantage that the function values $F(\underline{\gamma}_j)$, $\underline{\gamma}_j \ne \underline{x}_k$, have not been calculated. On the other hand, inequality (5.2) suggests that the points $\underline{y}_j$, $j = 1, 2, \ldots, m$, may be such that there is degeneracy or near-degeneracy in the interpolation conditions (1.2). Furthermore, it would be wasteful to calculate $F$ at all the new points if some of them are sufficiently close to old points, or if the use of an old point instead of a new one seems to be harmless. Therefore RESCUE employs an iterative procedure that begins with the set $\{\underline{\widehat{y}}_j = \underline{\gamma}_j : j = 1, 2, \ldots, m\}$, composed of the old point $\underline{\gamma}_1 = \underline{x}_k$ and the $m-1$ new points of the form (5.4) or (5.5). A typical iteration of RESCUE picks an old point, $\underline{y}_\ell$ say, that is not in the set $\{\underline{\widehat{y}}_j : j = 1, 2, \ldots, m\}$, and then asks the following question for $t = 1, 2, \ldots, m$. If $\underline{\widehat{y}}_t$ is not one of the points $\{\underline{y}_j : j = 1, 2, \ldots, m\}$, how safe is it to replace $\underline{\widehat{y}}_t$ by $\underline{y}_\ell$ in the set $\{\underline{\widehat{y}}_j : j = 1, 2, \ldots, m\}$. A criterion for safety is given later, and we make the safest choice of $t$. Further, the question is asked whether this choice is safe enough. Usually the answer is affirmative, and then the replacement of $\underline{\widehat{y}}_t$ by $\underline{y}_\ell$ in the set $\{\underline{\widehat{y}}_j : j = 1, 2, \ldots, m\}$ is made. Otherwise, the same questions are asked for other values of $\ell$, which may lead to a different replacement. Thus every successful iteration of RESCUE increases the number of old interpolation points in the set $\{\underline{\widehat{y}}_j : j = 1, 2, \ldots, m\}$ by one. This procedure ends if $m$–1 iterations are successful, because then $\{\underline{\widehat{y}}_j : j = 1, 2, \ldots, m\}$ has become the set of old interpolation points $\{\underline{y}_j : j = 1, 2, \ldots, m\}$. This is the situation that has been mentioned already, where the only change at the return from RESCUE is that ZMAT and BMAT have been recalculated. Alternatively, the iterative procedure of RESCUE ends when a sufficiently safe replacement of $\underline{\widehat{y}}_t$ by $\underline{y}_\ell$ cannot be found. The final set $\{\underline{\widehat{y}}_j : j = 1, 2, \ldots, m\}$ is the new set of interpolation points chosen by RESCUE, the function value $F(\underline{\widehat{y}}_j)$ being calculated by RESCUE if and only if $\underline{\widehat{y}}_j$ is not in the old set $\{\underline{y}_j : j = 1, 2, \ldots, m\}$.

We recall that the $Z$ and $H$ matrices just before the first iteration of RESCUE are generated for the interpolation points $\underline{\widehat{y}}_j = \underline{\gamma}_j$, $j = 1, 2, \ldots, m$, by techniques from Section 2. These matrices are updated on every iteration of RESCUE so that they remain the $Z$ and $H$ matrices of the points $\{\underline{\widehat{y}}_j : j = 1, 2, \ldots, m\}$. For each set $\{\underline{\widehat{y}}_j : j = 1, 2, \ldots, m\}$, we let $H$ be the matrix (2.7) when there are hats on the interpolation points of the definitions (2.8) and (2.9), and we let $Z$ be an $m \times (m-n-1)$ matrix with the property $\Omega = ZZ^T$. The procedures for updating $Z$ and $H$ are taken from Section 4. Specifically, when $\underline{\widehat{y}}_t$ is replaced by $\underline{y}_\ell$ in the

set $\{\underline{\widehat{y}}_j : j=1,2,\ldots,m\}$, the vectors $\underline{y}_i$, $i=1,2,\ldots,m$, in the definitions (4.10) and (4.12) are the vectors $\underline{\widehat{y}}_i$, $i=1,2,\ldots,m$, before the replacement is made, and $\underline{x}^+$ in the definitions (4.10) and (4.11) is the vector $\underline{y}_\ell$ of the current iteration of RESCUE. The integer $t$ chosen by RESCUE is retained in formulae (4.9), (4.11) and (4.14), while $s$ in equations (4.13) and (4.14) is the integer in $[1,m]$ such that $\underline{x}_k$ is the point $\underline{\widehat{y}}_s$. All other features of the updating are as in Section 4, including the device that avoids the storage of the $(m+1)$-th row and column of $H$. Thus, at the return from RESCUE, the $Z$ and $H$ matrices are those of the final set $\{\underline{\widehat{y}}_j : j=1,2,\ldots,m\}$.

The criterion for safety when $t$ is selected is derived from the denominator $\sigma$ that is going to occur when the updating procedure of the previous paragraph is applied. Specifically, in order to avoid divisions by unnecessarily small denominators, the freedom in $t$ is taken up by maximizing the quantity

$$\sigma \;=\; \alpha\,\beta + \tau^2 \;=\; H_{tt}\,(\tfrac{1}{2}\,\|\underline{y}_\ell - \underline{x}_k\|^4 - \underline{w}^T H\,\underline{w}) + (\underline{e}_t^T H\,\underline{w})^2, \qquad (5.6)$$

the right hand side being taken from expression (4.11) with $\underline{x}_0 = \underline{x}_k$. A valuable feature of the definition (4.10) of $\underline{w} \in \mathcal{R}^{m+n+1}$ is that it is independent of $t$, and the definition (4.12) gives $\underline{v} = \underline{e}_{m+1} \in \mathcal{R}^{m+n+1}$. Therefore not only $\beta = \frac{1}{2}\,\|\underline{y}_\ell - \underline{x}_k\|^4 - \underline{w}^T H\,\underline{w}$ but also the terms $H\,\underline{w}$ and $\underline{w}^T H\,\underline{w}$ of expression (4.13) are calculated before the cycle through the possible values of $t$. Thus it becomes inexpensive to select the integer $t$ in $[1,m]$ that maximizes the quantity (5.6) subject to $\underline{\widehat{y}}_t \notin \{\underline{y}_i : i=1,2,\ldots,m\}$.

Let $\widehat{\sigma}$ be the denominator (5.6) of the $t$ that has been selected. The question whether or not the choice of $t$ is safe enough is posed as a comparison of $\widehat{\sigma}$ with denominators that are typical for the introduction of $\underline{y}_\ell$. Caution is particularly important when only a small fraction of the integers $t$ in $[1,m]$ satisfy $\underline{\widehat{y}}_t \notin \{\underline{y}_i : i=1,2,\ldots,m\}$, but there would be no need for this constraint on $t$ if a point were being deleted from the set $\{\underline{\widehat{y}}_j : j=1,2,\ldots,m\}$ only to make room for $\underline{y}_\ell$. Therefore the criterion for sufficient safety pays attention to the right hand sides (5.6) for all integers $t$ in the set $\{1,2,\ldots,m\}\backslash\{s\}$, where $\underline{\widehat{y}}_s = \underline{x}_k$. Further, we exclude the contribution from $\alpha\beta$ to the right hand sides, because rounding errors can cause huge relative errors in $\beta$. Specifically, BOBYQA replaces $\underline{\widehat{y}}_t$ by $\underline{y}_\ell$ in the set $\{\underline{\widehat{y}}_j : j=1,2,\ldots,m\}$ if and only if the condition

$$\widehat{\sigma} \;>\; 0.01\,\max\{(\underline{e}_j^T H\,\underline{w})^2 : j \in \{1,2,\ldots,m\}\backslash\{s\}\} \qquad (5.7)$$

is achieved, the multiplier 0.01 being included because it seems to be suitable in numerical experiments.

The right hand side of expression (5.7) is positive in practice, and the following argument shows that this happens also in theory. We recall from equations (4.15) and (4.18) that, for $j=1,2,\ldots,m$, the term $\underline{e}_j^T H\,\underline{w}$ is the value $\Lambda_j(\underline{x}^+) = \Lambda_j(\underline{y}_\ell)$ of a Lagrange function that satisfies $\Lambda_j(\underline{\widehat{y}}_i) = \delta_{ij}$, $1 \le i,j \le m$. Further, these Lagrange functions have the property

$$\textstyle\sum_{j=1}^m p(\underline{\widehat{y}}_j)\,\Lambda_j(\underline{x}) \;=\; p(\underline{x}), \qquad \underline{x} \in \mathcal{R}^n, \qquad (5.8)$$

where $p$ is any linear polynomial. This equation with the choice $\underline{x}=\underline{y}_\ell$ provides the relation

$$\textstyle\sum_{j=1}^m p(\underline{\widehat{y}}_j)\, \underline{e}_j^T H \underline{w} \;=\; p(\underline{y}_\ell), \tag{5.9}$$

and $p$ can satisfy both $p(\underline{\widehat{y}}_s)=0$ and $p(\underline{y}_\ell)\neq 0$. It follows as required that at least one of the terms $\underline{e}_j^T H \underline{w}$, $j\in\{1,2,\ldots,m\}\backslash\{s\}$ is nonzero.

The choices of $\ell$ on each iteration of RESCUE take two factors into consideration. Firstly, if the final set $\{\underline{\widehat{y}}_j : j=1,2,\ldots,m\}$ is without some of the original points $\underline{y}_i$, $i=1,2,\ldots,m$, we prefer the rejected points to be relatively far from $\underline{x}_k$. Secondly, if the failure of condition (5.7) excludes $\underline{y}_\ell$ on the current iteration of RESCUE, then the same $\underline{y}_\ell$ is given lower priority on future iterations, because it is unlikely that intermediate iterations will help the acceptance of $\underline{y}_\ell$. Therefore each point $\underline{y}_i$, $i=1,2,\ldots,m$, is given the score $\psi_i = \|\underline{y}_i - \underline{x}_k\|$ before the first iteration of RESCUE, and we let $\psi_*$ be the greatest of these scores. Whenever the test (5.7) fails for a choice of $\ell$, then the score of $\underline{y}_\ell$ is increased by adding $\psi_*$ to $\psi_\ell$, but, if the test (5.7) is satisfied, then $\psi_\ell$ is set to zero and $\underline{y}_\ell$ replaces $\underline{\widehat{y}}_t$ in the set $\{\underline{\widehat{y}}_j : j=1,2,\ldots,m\}$, by applying the procedure that has been described already. It follows that, at the beginning of each iteration of RESCUE, the point $\underline{y}_i$, $i=1,2,\ldots,m$, is in the set $\{\underline{\widehat{y}}_j : j=1,2,\ldots,m\}$ if and only if $\psi_i$ is zero. Every choice of $\ell$ by RESCUE is such that $\psi_\ell$ is the least positive score in the set $\{\psi_i : i=1,2,\ldots,m\}$. These choices continue on the current iteration until condition (5.7) is achieved, or until a new $\ell$ is the same as a choice that has been tried already on the current iteration of RESCUE. There are no more iterations in the latter case, the final set $\{\underline{\widehat{y}}_j : j=1,2,\ldots,m\}$ being the next set of interpolation points.

Let $\mathcal{T}$ be the subset of integers $t$ in the interval $[1,m]$ such that $\underline{\widehat{y}}_t$ is not in the original set $\{\underline{y}_i : i=1,2,\ldots,m\}$ after all the iterations of RESCUE. If $\mathcal{T}$ is empty, the work of RESCUE is complete, because the ordering of the points $\{\underline{\widehat{y}}_j : j=1,2,\ldots,m\}$ by RESCUE provides the property $\underline{\widehat{y}}_t=\underline{y}_t$, $t\notin\mathcal{T}$. Otherwise, the new interpolation conditions

$$Q_k(\underline{\widehat{y}}_t) = F(\underline{\widehat{y}}_t), \qquad t\in\mathcal{T}, \tag{5.10}$$

are satisfied by updating $Q_k$ in the following way. We retain the form (4.6) of $\nabla^2 Q$, but the points $\underline{y}_t$, $t\in\mathcal{T}$, are unwanted. Therefore $\sum_{\ell\in\mathcal{T}} \mu_\ell\, (\underline{y}_\ell - \underline{x}_0)\, (\underline{y}_\ell - \underline{x}_0)^T$ is added to the matrix $M$, in order that the parameters $\mu_\ell$, $\ell\in\mathcal{T}$, can be set to zero, with no change to $\mu_\ell$, $\ell\notin\mathcal{T}$. Thus, before it is updated, $Q_k$ has the second derivative matrix

$$\nabla^2 Q_k \;=\; M + \textstyle\sum_{\ell=1}^m \mu_\ell\, (\underline{\widehat{y}}_\ell - \underline{x}_0)\, (\underline{\widehat{y}}_\ell - \underline{x}_0)^T. \tag{5.11}$$

The current $H$ matrix provides the Lagrange functions $\Lambda_i(\underline{x})$, $\underline{x}\in\mathcal{R}^n$, that satisfy $\Lambda_i(\underline{\widehat{y}}_j)=\delta_{ij}$, $1\le i,j\le m$, the freedom in $\Lambda_i$ being taken up as usual by minimizing $\|\nabla^2\Lambda_i\|_F$. Therefore the procedure for updating $Q_k$ is as follows. For each integer $t$ in $\mathcal{T}$, we replace $Q_k$ by the quadratic function

$$Q_k(\underline{x}) + \{F(\underline{\widehat{y}}_t) - Q_k(\underline{\widehat{y}}_t)\}\, \Lambda_t(\underline{x}), \qquad \underline{x}\in\mathcal{R}^n, \tag{5.12}$$

this task being completed before beginning the updating of $Q_k$ for the next value of $t$. We keep $Q_k$ in the form (4.7), where $\underline{g}_k = \underline{\nabla} Q_k(\underline{x}_k)$, with the second derivative matrix (5.11). It follows from equation (4.3) that, for each $t \in \mathcal{T}$, the parameters $\mu_\ell$ of expression (5.11) are overwritten by $\mu_\ell + \lambda_\ell$, $\ell = 1, 2, \ldots, m$, where $\underline{\lambda} \in \mathcal{R}^m$, is the $t$-th column of $\Omega = ZZ^T$ multiplied by $\{F(\underline{\widehat{y}}_t) - Q_k(\underline{\widehat{y}}_t)\}$, but there is no change to the matrix $M$. The updating of $\underline{g}_k$ for each $t \in \mathcal{T}$ is taken from the right hand side of formula (4.8), but the sum over $\ell$ is zero due to $\underline{x}_0 = \underline{x}_k$. Therefore it is sufficient to add $\underline{g} = \{F(\underline{\widehat{y}}_t) - Q_k(\underline{\widehat{y}}_t)\} \, \underline{\nabla} \Lambda_t(\underline{x}_k)$ to $\underline{g}_k$. We recall from Section 4 that the components of $\underline{\nabla} \Lambda_t(\underline{x}_k)$ are the last $n$ elements of the $t$-th column of $H$, which are the elements of the $t$-th column of BMAT. The matrix $H$ remains fixed throughout the updating of $Q_k$. Thus RESCUE constructs a new quadratic model that would interpolate all the function values $F(\underline{\widehat{y}}_j)$, $j = 1, 2, \ldots, m$, in exact arithmetic.

We take the view that, if subroutine RESCUE is called on the $k$-th iteration of RESCUE, then all its work is a task within the $k$-th iteration to try to correct serious errors that have occurred in the matrix $H$. Therefore BOBYQA returns to the usual operations of the $k$-th iteration after the calculations of RESCUE are complete, although $|\mathcal{T}|$ new values of the objective function are required for the conditions (5.10). Therefore the chosen points $\underline{\widehat{y}}_j$ and their function values $F(\underline{\widehat{y}}_j)$, $j = 1, 2, \ldots, m$, replace the old values of $\underline{y}_j$ and $F(\underline{y}_j)$, $j = 1, 2, \ldots, m$, in agreement with the new matrix $H$. Further, $\underline{x}_k$ is shifted if necessary in order to preserve equation (1.3), with the corresponding change to $\underline{g}_k = \underline{\nabla} Q_k(\underline{x}_k)$. Calls of RESCUE are unusual unless unattainable accuracy is requested by the user of BOBYQA. They are expensive, because the construction of the inverse of a general $(m+n+1) \times (m+n+1)$ matrix requires $\mathcal{O}(m^3)$ operations. Some work and storage are saved by taking advantage of the property that the vectors $\underline{\gamma}_j - \underline{x}_0$, $j = 2, 3, \ldots, m$, have only one or two nonzero components, as shown in formulae (5.4) and (5.5).

## 6. Other features of BOBYQA

The first topic of this section continues the description of a "trust region" iteration of BOBYQA when the step $\underline{d}_k$ from $\underline{x}_k$, given by the procedure in the first half of Section 3, satisfies $\|\underline{d}_k\| \geq \frac{1}{2}\Delta_k$. Usually the function value $F(\underline{x}_k + \underline{d}_k)$ is calculated, and the change (1.6) is made to the interpolation points, the value of $t$ being specified below. It is possible, however, that condition (5.2) is going to invoke a call of RESCUE, because of severe errors in the matrix $H$, which is likely to modify $Q_k(\underline{x})$, $\underline{x} \in \mathcal{R}^n$, and then a new trust region step $\underline{d}_k$ would be constructed. In this case $F(\underline{x}_k + \underline{d}_k)$ would not be required for the first choice of $\underline{d}_k$. Therefore a feature of BOBYQA is that the decision whether or not to call RESCUE is taken before the calculation of $F(\underline{x}_k + \underline{d}_k)$.

The decision depends on a choice of $t$, made in a way that is similar to the one in the paragraph that includes equation (5.6). In RESCUE, however, the point

$\underline{\widehat{y}}_t$ that is dropped from the set $\{\underline{\widehat{y}}_j : j=1,2,\ldots,m\}$ to make room for $\underline{y}_\ell$ always has the property $\frac{1}{2}\Delta_k \le \|\underline{\widehat{y}}_t - \underline{x}_k\| \le \sqrt{2}\,\Delta_k$, due to formulae (5.4) and (5.5) and the values of $\alpha_i$ and $\beta_i$, but now some of the distances $\|\underline{y}_j - \underline{x}_k\|$, $j=1,2,\ldots,m$, may be much larger than $\Delta_k$. Priority is given to the deletion of an interpolation point that is relatively far from $\underline{x}_k$. Specifically, $t$ is set to the integer in the set $\{1,2,\ldots,m\}\backslash\{s\}$ that maximizes the weighted denominator

$$\begin{aligned} &\max[1,\ \|\underline{y}_t - \underline{x}_k\|^2/\Delta_k^2]\,\sigma \;=\; \max[1,\ \|\underline{y}_t - \underline{x}_k\|^2/\Delta_k^2]\,(\alpha\,\beta + \tau^2) \\ &=\; \max[1,\ \|\underline{y}_t - \underline{x}_k\|^2/\Delta_k^2]\,\left\{ H_{tt}\,(\tfrac{1}{2}\,\|\underline{x}^+ - \underline{x}_0\|^4 - \underline{w}^T H\,\underline{w}) + (\underline{e}_t^T H\,\underline{w})^2 \right\}, \qquad (6.1) \end{aligned}$$

where $s$ is defined as usual by $\underline{x}_k = \underline{y}_s$, where $\underline{x}^+ = \underline{x}_k + \underline{d}_k$, and where $\underline{w} \in \mathcal{R}^{m+n+1}$ has the components (4.10), so again $\underline{w}$ and $H\underline{w}$ are independent of $t$. Thus the selection of $t$ is straightforward, using the identities (4.13) because the $(m+1)$-th row and column of $H$ are not available.

Let $\alpha$, $\beta$, $\tau$ and $\sigma$ be the calculated values of the parameters (4.11) for the chosen integer $t$. Subroutine RESCUE is invoked if condition (5.2) holds, in order to try to correct the unacceptable errors in $H$. We recall that the current interpolation points $\underline{y}_i$, $i=1,2,\ldots,m$, are changed by RESCUE if and only if the set $\mathcal{T}$ of the constraints (5.10) is nonempty. At the return from RESCUE, there is a branch back to the beginning of the current iteration for the construction of another "trust region" step $\underline{d}_k$, which is followed automatically by the procedure of the previous paragraph that selects $t$. Hence there are going to be new values of $\alpha$, $\beta$, $\tau$ and $\sigma$, and then condition (5.2) is tested again. If it still holds, we ask whether $\mathcal{T}$ was nonempty on the previous call of RESCUE. There is another call of RESCUE if the answer is affirmative, but BOBYQA has to make an error return if $\mathcal{T}$ was empty, because then the results from another application of RESCUE would be the same as the results from the most recent call. This error return is very rare.

The cycle in the previous paragraph has to end, because, on every call of RESCUE except possibly the last one, there is an increase in the total number of calculations of $F$, and an upper bound on this number is supplied by the user of BOBYQA. We assume therefore that $\underline{d}_k$ and $t$ have been chosen with an acceptable denominator $\sigma$. At this stage $F(\underline{x}_k + \underline{d}_k)$ is calculated, and $\underline{x}_{k+1}$ is defined by equation (1.4). A complication arises in the case $F(\underline{x}_k + \underline{d}_k) < F(\underline{x}_k)$, because then the distance from $\underline{y}_t$ to $\underline{x}_{k+1}$ becomes more important than the distance from $\underline{y}_t$ to $\underline{x}_k$, $t \in \{1,2,\ldots,m\}\backslash\{s\}$. Therefore the procedure for selecting $t$ is repeated after replacing $\underline{x}_k$ by $\underline{x}_{k+1}$ in the weighted denominator (6.1). If the calculated parameters of the new $t$ satisfy $\sigma = \alpha\beta + \tau^2 > \frac{1}{2}\tau^2$, then $t$ is given its new value in the definition (1.6) of the interpolation points for the next iteration. Otherwise, and also in the case $F(\underline{x}_k + \underline{d}_k) \ge F(\underline{x}_k)$, we pick the $t$ that is known to provide an acceptable $\sigma$. The subsequent updatings of $Q(\underline{x})$, $\underline{x} \in \mathcal{R}^n$, and $H$ are taken from Section 4. The choice of $\Delta_{k+1}$ on a "trust region" iteration receives attention later.

Next the description of an "alternative" iteration is continued; it also includes some calls of RESCUE if $\sigma$ is too small. The integer $t$ of formula (1.6) is provided by equation (3.7), and then subroutine ALTMOV constructs the steps $\underline{d}_k$ and $\underline{c}_k$ that give relatively large values of the modulus of the function $\Lambda_t(\underline{x}_k+\underline{d})$, $\|\underline{d}\|\leq\Delta_k$, as described in Section 3. At the return from ALTMOV, it is assumed provisionally that equations (4.9)–(4.11), with $\underline{x}^+=\underline{x}_k+\underline{d}_k$, are going to be used to update $H$. Therefore the relevant parameters (4.11) are calculated by software that is shared with this part of a "trust region" iteration. The resultant values of $\alpha$, $\beta$ and $\tau=\underline{e}_t^T H\underline{w}$ are the terms $H_{tt}$, $\beta(\underline{x}_k+\underline{d}_k)$ and $\Lambda_t(\underline{x}_k+\underline{d}_k)$ on the right hand side of expression (3.12), and the term $\{\Lambda_t(\underline{x}_k+\underline{c}_k)\}^2$ on the left hand side is provided by ALTMOV. We recall that, if condition (3.12) holds, then $\underline{d}_k$ is replaced by $\underline{c}_k$; also the parameters $\alpha$, $\beta$ and $\tau$ are recalculated.

Usually the work of an "alternative" iteration is completed by generating the new function value $F(\underline{x}_k+\underline{d}_k)$, by making the change (1.6) to the interpolation points, by applying the updating procedures of Section 4 to $Q$ and $H$, and by making the choice (1.4) of $\underline{x}_{k+1}$. The iteration includes too the test (5.2) on the denominator $\sigma$, which is tried before $F(\underline{x}_k+\underline{d}_k)$ is calculated. If the test is satisfied, it is possible that RESCUE has been called already on the current "alternative" iteration, and then another call would not provide any new information, so BOBYQA makes the very rare error return mentioned earlier in this section. Otherwise condition (5.2) triggers a call of RESCUE, because the errors in $H$ are unacceptable.

On the return from RESCUE on an "alternative" iteration, one of the following two branches is taken. If the interpolation points $\underline{y}_i$, $i=1,2,\ldots,m$, have not been altered, the set $\mathcal{T}$ of the constraints (5.10) being empty, there is a branch back to the call of ALTMOV, which supplies $\underline{d}_k$ and $\underline{c}_k$ for the new matrix $H$, with no change to the integer $t$, because equation (3.7) remains valid. The other branch is to the beginning of a new "trust region" iteration, because the change to the interpolation points by RESCUE is assumed to have helped not only the accuracy of $Q$ and $H$, but also the linear independence of the conditions (1.2). Further, the values $F(\underline{y}_j)$ at all the new interpolation points have been found by RESCUE, the relevant updating of $Q$ and $H$ has been done, and $\underline{x}_k$ has been shifted if required by condition (1.3). Our remarks on the use of RESCUE by BOBYQA are complete.

A technique that helps to keep the interpolation points $\underline{y}_j$, $j=1,2,\ldots,m$, apart is taken from NEWUOA. It employs a lower bound, $\rho_k$ say, on $\Delta_k$ for every $k$. The sequence $\rho_k$, $k=1,2,3,\ldots$, decreases monotonically, with $\rho_{k+1}=\rho_k$ on most iterations. The decrease $\rho_{k+1}<\rho_k$ occurs only when it seems to be necessary for further progress, the usual decrease being $\rho_{k+1}=0.1\rho_k$. For example, trust region radii that satisfy $\Delta_k\geq 0.1$ may be suitable on the early iterations, but, in order to achieve the required accuracy, steps of length only $10^{-6}$ or less may have to be taken eventually. The purpose of the bound

$$\Delta_k \geq \rho_k, \qquad k=1,2,3,\ldots, \tag{6.2}$$

is to postpone the use of short steps until late in the calculation, as mentioned in the opening paragraph of Section 3. The user of BOBYQA has to supply the initial and final values of $\rho_k$, namely $\rho_{\rm beg}$ and $\rho_{\rm end}$, the initial trust region radius being $\Delta_1 = \rho_{\rm beg}$. In many numerical experiments, the distance from the final $\underline{x}_k$ to a local minimum of $F(\underline{x})$, $\underline{x} \in \mathcal{R}^n$, is less than $10\rho_{\rm end}$, unless $\rho_{\rm end}$ is so small that such accuracy is unattainable.

The reader of this report has not been troubled by $\rho_k$ so far, but it is relevant to the opening paragraph of Section 3. Indeed, although it is stated there that a "trust region" step $\underline{d}_k$ may be rejected if it satisfies $\|\underline{d}_k\| < \frac{1}{2}\Delta_k$, the rejection occurs only in the case $\|\underline{d}_k\| < \frac{1}{2}\rho_k$. Similarly, $\frac{1}{2}\Delta_k$ should be replaced by $\frac{1}{2}\rho_k$ in the first sentence of this section.

The choice of $\Delta_{k+1}$ on a "trust region" iteration that calculates $F(\underline{x}_k + \underline{d}_k)$ depends on the ratio

$$r_k = \frac{F(\underline{x}_k) - F(\underline{x}_k + \underline{d}_k)}{Q_k(\underline{x}_k) - Q_k(\underline{x}_k + \underline{d}_k)} = \frac{Q_k(\underline{x}_k) - F(\underline{x}_k + \underline{d}_k)}{Q_k(\underline{x}_k) - Q_k(\underline{x}_k + \underline{d}_k)}. \tag{6.3}$$

An error return occurs in the highly unusual case when the denominator of $r_k$ is not positive in practice. Otherwise, the trust region radius tends to be increased or decreased if the estimate $Q_k(\underline{x}_k + \underline{d}_k) \approx F(\underline{x}_k + \underline{d}_k)$ is favourable or unfavourable, respectively. Specifically, the formula

$$\Delta_{k+1} = \begin{cases} \min\,[\,\frac{1}{2}\,\Delta_k,\ \|\underline{d}_k\|\,], & r_k \le 0.1, \\ \max\,[\,\frac{1}{2}\,\Delta_k,\ \|\underline{d}_k\|\,], & 0.1 < r_k \le 0.7, \\ \max\,[\,\frac{1}{2}\,\Delta_k,\ 2\,\|\underline{d}_k\|\,], & r_k > 0.7, \end{cases} \tag{6.4}$$

is applied, except that $\Delta_{k+1}$ is set to $\rho_k$ if the value (6.4) is at most $1.5\rho_k$. Usually $\underline{d}_k$ is a step to the trust region boundary of the $k$-th iteration, and then $\|\underline{d}_k\|$ is the same as $\Delta_k$ in equation (6.4).

Only the last line of formula (6.4) can provide the increase $\Delta_{k+1} > \Delta_k$ in the trust region radius. The reduction $\Delta_{k+1} < \Delta_k$ can be made by this formula and in just two other situations, the choice $\Delta_{k+1} = \Delta_k$ being made automatically on all other iterations. One situation is when a "trust region" step $\underline{d}_k$ is rejected because it satisfies $\|\underline{d}_k\| < \frac{1}{2}\rho_k$. In this case the quantity

$$\delta_k = \max\,\{\|\underline{y}_j - \underline{x}_k\| : j = 1, 2, \ldots, m\} \tag{6.5}$$

is available, and $\Delta_k$ is overwritten by the term $\min\,[\frac{1}{10}\Delta_k, \frac{1}{2}\delta_k]$, except that $\Delta_k$ is set to $\rho_k$ if this term is at most $1.5\rho_k$. The other situation is when $\rho_{k+1} < \rho_k$ occurs in the bounds (6.2), the criteria for this reduction being given after the next paragraph. Then the formula $\Delta_{k+1} = \max\,[\frac{1}{2}\rho_k, \rho_{k+1}]$ is applied. Another refinement is that $\Delta_k$ may be reduced temporarily before a call of ALTMOV. Indeed, if the maximum distance (6.5) from $\underline{x}_k$ to an interpolation point is less than $10\Delta_k$, then $\Delta_k$ is reduced to $\max\,[0.1\delta_k, \rho_k]$ before the call of ALTMOV, the

old value of $\Delta_k$ being restored at the return. The specification of the sequence $\Delta_k$, $k=1,2,3,\ldots$, is complete.

If the $k$-th iteration is of "alternative" type, then the ($k$+1)-th iteration always calculates a "trust region" step with $\Delta_{k+1}=\Delta_k$ and $\rho_{k+1}=\rho_k$. Therefore the work with the current $\rho_k$ is found to be finished only if the current iteration is of "trust region" type. When a reduction in the right hand side of expression (6.2) is required, BOBYQA applies the formula

$$\rho_{k+1} = \begin{cases} \rho_{\rm end}, & \rho_k \le 16\,\rho_{\rm end}, \\ (\rho_k\,\rho_{\rm end})^{1/2}, & 16\,\rho_{\rm end} < \rho_k \le 250\,\rho_{\rm end}, \\ 0.1\,\rho_{\rm end}, & \rho_k > 250\,\rho_{\rm end}, \end{cases} \tag{6.6}$$

which is taken from NEWUOA (Powell, 2006). We see that, in the usual case $\rho_{\rm end} \le \frac{1}{4}\rho_{\rm beg}$, every application of equation (6.6) decreases the lower bound on the trust region radii by a factor from the interval $[4,16]$. The description of the sequence $\rho_k$, $k=1,2,3,\ldots$, until termination is also complete.

Next we address the case when $F(\underline{x}_k+\underline{d}_k)$ is calculated on a "trust region" iteration. If the strict reduction $F(\underline{x}_k+\underline{d}_k)<F(\underline{x}_k)$ is achieved, the work with the current $\rho_k$ is not yet finished. Further, the view is taken that "trust region" iterations are a good thing if the ratio (6.3) has the property $r_k\ge 0.1$, and then the next iteration is always of "trust region" type. Thus many consecutive "trust region" iterations may occur, which tends to stretch out the set of current interpolation points instead of forming a cluster. If $r_k<0.1$ holds, however, then BOBYQA has to decide whether the next iteration is going to be of "alternative" type in order to improve the quadratic model. At this stage, $\underline{x}_{k+1}$ and the new interpolation points $\underline{\widehat{y}}_j$, $j=1,2,\ldots,m$, have become available by applying formulae (1.4) and (1.6), and $\delta_{k+1}$ is set to the greatest of the distances $\|\underline{\widehat{y}}_j-\underline{x}_{k+1}\|$, $j=1,2,\ldots,m$, which agrees with the definition (6.5). The next iteration is of "alternative" type if and only if $\delta_{k+1}>\max\,[2\Delta_{k+1},10\rho_k]$ is satisfied, because then one (or more) of the new interpolation points is relatively far from $\underline{x}_{k+1}$. Otherwise, the next iteration is of "trust region" type with $\rho_{k+1}=\rho_k$, not only in the situation $F(\underline{x}_k+\underline{d}_k)<F(\underline{x}_k)$ that has been mentioned already, but also in the case $\max\,[\|\underline{d}_k\|,\Delta_{k+1}]>\rho_k$. The only remaining possibility, on a "trust region" iteration that calculates $F(\underline{x}_k+\underline{d}_k)$, is characterised by the conditions

$$\Delta_{k+1}=\rho_k, \quad \|\underline{d}_k\|\le\rho_k, \quad \delta_{k+1}\le 10\,\rho_k \quad \text{and} \quad r_k\le 0. \tag{6.7}$$

If they all hold, the time has come for the decrease $\rho_{k+1}<\rho_k$ or for termination of the iterations of BOBYQA, the latter option being taken if and only if $\rho_k$ has reached its lower bound $\rho_{\rm end}$.

We now turn to the case when a "trust region" step satisfies $\|\underline{d}_k\|<\frac{1}{2}\rho_k$. Then either $\underline{d}_k$ is replaced by the step of an "alternative" iteration, as indicated at the beginning of Section 3, or the work with the current value of $\rho_k$ is complete. The latter option is always taken if all the points $\underline{y}_j$, $j=1,2,\ldots,m$, are sufficiently close to $\underline{x}_k$. Specifically, corresponding to the third part of expression (6.7), if the

maximum distance (6.5) satisfies $\delta_k \leq 10\rho_k$, then, as at the end of the previous paragraph, the time has come for the decrease $\rho_{k+1} < \rho_k$ or for termination of the iterations of BOBYQA.

It is important to efficiency, however, to include a procedure for ending the work with the current $\rho_k$ when both $\|\underline{d}_k\| < \frac{1}{2}\rho_k$ and $\delta_k > 10\rho_k$ hold, because the following situation is not unusual. A quadratic model may have been so successful on a previous iteration that $\|\underline{x}_k - \underline{x}_*\|$ is now much less than $\rho_k$, where $\underline{x}_*$ is still the optimal vector of variables. Further, the models may continue to be so successful that, whenever $\underline{d}_k$ is a "trust region" step, the distance $\|\underline{x}_k + \underline{d}_k - \underline{x}_*\|$ is also much less than the current $\rho_k$. It follows from the triangle inequality $\|\underline{d}_k\| \leq \|\underline{x}_k - \underline{x}_*\| + \|\underline{x}_k + \underline{d}_k - \underline{x}_*\|$ that every "trust region" step may be excluded by the requirement $\|\underline{d}_k\| \geq \frac{1}{2}\rho_k$ until $\rho_k$ is reduced. Therefore BOBYQA includes the following technique for giving up the current value of $\rho_k$ when the models seem to be sufficiently accurate. It is an extension for the bounds (1.1) of a similar procedure in NEWUOA (Powell, 2006).

The technique employs a crude estimate of the accuracy of the approximation $Q_k(\underline{x}_k + \underline{d}) \approx F(\underline{x}_k + \underline{d})$, $\|\underline{d}\| \leq \rho_k$. Each estimate has the form

$$\varepsilon_{\max} = \max\{|F(\underline{x}_\ell + \underline{d}_\ell) - Q_\ell(\underline{x}_\ell + \underline{d}_\ell)| : \ell \in \{k-3, k-2, k-1\}\}, \tag{6.8}$$

where $F(\underline{x}_\ell + \underline{d}_\ell)$, $k-3 \leq \ell \leq k-1$, are the three most recently calculated values of the objective function. The notation $\underline{x}_\ell + \underline{d}_\ell$ indicates that this vector of variables is confined to the trust region of the $\ell$-th iteration, which has centre $\underline{x}_\ell$ and radius $\Delta_\ell$, say, giving the bound $\|\underline{d}_\ell\| \leq \Delta_\ell$. The meaning of $\underline{d}_\ell$ may have been changed during the $\ell$-th iteration, because it could begin as a "trust region" step that satisfies $\|\underline{d}_\ell\| < \frac{1}{2}\rho_\ell$, but then, because $F(\underline{x}_\ell + \underline{d}_\ell)$ is actually calculated, the step $\underline{d}_\ell$ must have been switched to one of "alternative" type. The value (6.8) is not available until the fourth iteration. Further, it is ignored if RESCUE is called on or after the $(k-3)$-rd iteration. Otherwise, we say that $\varepsilon_{\max}$ is "usable" if and only if the steps $\underline{d}_\ell$ in expression (6.8) have the property $\|\underline{d}_\ell\| \leq \rho_k$, $k-3 \leq \ell \leq k-1$.

Whenever $\|\underline{d}_k\| < \frac{1}{2}\rho_k$ occurs, $\underline{d}_k$ being a "trust region" step, the question is asked whether a "usable" $\varepsilon_{\max}$ exists. The question is irrelevant if $\delta_k \leq 10\rho_k$ prevails, because then, as mentioned already, the calculations with the current $\rho_k$ are complete. Moreover, if $\delta_k > 10\rho_k$ holds and if the answer to the question is negative, then the current iteration is switched to one of "alternative" type. In the remaining situation, characterised by $\|\underline{d}_k\| < \frac{1}{2}\rho_k$, $\delta_k > 10\rho_k$ and $\varepsilon_{\max}$ being "usable", we give up the current value of $\rho_k$ if and only if the following tests suggest that the main optimization calculation has a local minimum at a point $\underline{x}_*$ in the trust region $\{\underline{x} : \|\underline{x} - \underline{x}_k\| \leq \rho_k\}$.

The following criterion is taken from NEWUOA. When $\underline{d}_k$ is calculated by the conjugate gradient procedure of Section 3, we let $\mathcal{S}$ be the set of search directions such that the steps taken along these directions are not restricted by the bounds $\underline{a} \leq \underline{x} \leq \underline{b}$. The second derivative terms $\underline{s}^T \nabla^2 Q_k \underline{s}$, $\underline{s} \in \mathcal{S}$, are available and, if there is no interference later from restarts due to bounds becoming active, the conjugacy properties provide $\underline{s}^T \underline{\nabla} Q_k(\underline{x}_k + \underline{d}_k) = 0$, $\underline{s} \in \mathcal{S}$. In this case a move from

$\underline{x}_k+\underline{d}_k$ to $\underline{x}_k+\underline{d}_k+\theta\underline{s}$, where $\theta$ satisfies $\|\underline{d}_k+\theta\underline{s}\|=\rho_k$, yields the increase

$$\begin{aligned} Q_k(\underline{x}_k+\underline{d}_k+\theta\underline{s}) &= Q_k(\underline{x}_k+\underline{d}_k) + \tfrac{1}{2}\,\theta^2 \underline{s}^T \nabla^2 Q_k\, \underline{s} \\ &> Q_k(\underline{x}_k+\underline{d}_k) + \tfrac{1}{8}\,\rho_k^2\, \|\underline{s}\|^{-2}\, \underline{s}^T \nabla^2 Q_k\, \underline{s}, \qquad \underline{s}\in\mathcal{S}, \end{aligned} \tag{6.9}$$

the last line being due to the remark that $\|\underline{d}_k+\theta\underline{s}\|=\rho_k$ and $\|\underline{d}_k\|<\frac{1}{2}\rho_k$ imply $\|\theta\underline{s}\|>\frac{1}{2}\rho_k$. Changes to $Q_k$ provide guidance on changes to $F$ and we recall the definition (6.8). Indeed, if the inequalities

$$\varepsilon_{\max} \;\le\; \tfrac{1}{8}\,\rho_k^2\, \|\underline{s}\|^{-2}\, \underline{s}^T \nabla^2 Q_k\, \underline{s}, \qquad \underline{s}\in\mathcal{S}, \tag{6.10}$$

are satisfied, then the relations (6.9) provide some support for the suggestion that a move from $\underline{x}_k+\underline{d}_k$ to the trust region boundary is not going to reduce the objective function $F$.

Another criterion is also employed because of the constraints $\underline{a}\le\underline{x}\le\underline{b}$. We form a set $\mathcal{V}$ of multiples of coordinate directions, the vector $\rho\underline{e}_i$ or $-\rho\underline{e}_i$ being included in $\mathcal{V}$ if and only if the $i$-th component of $\underline{x}_k+\underline{d}_k$ is at its lower bound $a_i$ or upper bound $b_i$, respectively. All the points $\underline{x}=\underline{x}_k+\underline{d}_k+\underline{v}$, $\underline{v}\in\mathcal{V}$, satisfy the bound constraints, and usually the directional derivatives $\underline{v}^T\underline{\nabla}Q_k(\underline{x}_k+\underline{d}_k)$, $\underline{v}\in\mathcal{V}$, are positive. We take the view that the iterations with the current $\rho_k$ should continue if one (or more) of the differences $Q_k(\underline{x}_k+\underline{d}_k+\underline{v})-Q_k(\underline{x}_k+\underline{d}_k)$, $\underline{v}\in\mathcal{V}$, is less than $\varepsilon_{\max}$, except that we prefer to ignore second derivatives if the first order part of a difference is sufficiently large. Thus the new test on $\varepsilon_{\max}$ is the condition

$$\varepsilon_{\max} \;\le\; \max\,[\,\underline{v}^T\underline{\nabla}Q_k(\underline{x}_k+\underline{d}_k),\; \underline{v}^T\underline{\nabla}Q_k(\underline{x}_k+\underline{d}_k)+\tfrac{1}{2}\,\underline{v}^T\nabla^2 Q_k\,\underline{v}\,], \qquad \underline{v}\in\mathcal{V}. \tag{6.11}$$

When the length of a "trust region" step of BOBYQA is less than $\frac{1}{2}\rho_k$ and when a "usable" $\varepsilon_{\max}$ exists, it is decided that the current quadratic model is adequate not only in the case $\delta_k\le 10\rho_k$ but also if all the conditions (6.10) and (6.11) are achieved. Then $\rho_k$ is decreased or termination occurs, instead of a switch to an "alternative" iteration.

The first iteration at the beginning of the calculation and after every decrease in $\rho_k$ is always of "trust region" type. It happens often at termination that the final $\underline{d}_k$ is a "trust region" step that satisfies $\|\underline{d}_k\|<\frac{1}{2}\rho_{\text{end}}$. Then $\underline{x}_k+\underline{d}_k$ may be much closer than $\underline{x}_k$ to a minimum of the objective function; therefore the new function value $F(\underline{x}_k+\underline{d}_k)$ is calculated, in order that BOBYQA can return $\underline{x}_k$ or $\underline{x}_k+\underline{d}_k$ as the final vector of variables, in the case $F(\underline{x}_k+\underline{d}_k)\ge F(\underline{x}_k)$ or $F(\underline{x}_k+\underline{d}_k)<F(\underline{x}_k)$, respectively. The description of the choices that are made between "trust region" and "alternative" steps is complete.

The purpose of yet another technique of BOBYQA and NEWUOA is to avoid severe inefficiencies if the elements of $\nabla^2 Q_k$ are much too large. For example, large second derivatives may be inherited from the initial model $Q_1$ if $F(\underline{x})$, $\underline{x}\in\mathcal{R}^n$, increases at a fast exponential rate in some regions of $\mathcal{R}^n$, and if the initial vector of variables $\underline{x}_0$ is in such a region and far from a local minimum. Extra help may be needed to reduce $\|\nabla^2 Q_k\|$, because in general the change $\|\nabla^2 Q_{k+1}-\nabla^2 Q_k\|_F$ is

as small as possible subject to the new interpolation conditions (1.5). Therefore, after the calculation of $F(\underline{x}_k+\underline{d}_k)$ on a "trust region" iteration, and after the updating of Section 4 is complete, the new quadratic model $Q_{k+1}$ is compared with $Q_{k+1}^{\text{alt}}(\underline{x})$, $\underline{x}\in\mathcal{R}^n$, say, which is the quadratic that minimizes $\|\nabla^2 Q_{k+1}^{\text{alt}}\|_F$ subject to $Q_{k+1}^{\text{alt}}(\widehat{\underline{y}}_j)=F(\widehat{\underline{y}}_j)$, $j=1,2,\ldots,m$. By employing the current inverse matrix $H$ of expression (2.7), the parameters of $Q_{k+1}^{\text{alt}}$ are generated in only $\mathcal{O}(m^2)$ operations, including the gradient $\underline{\nabla} Q_{k+1}^{\text{alt}}(\underline{x}_{k+1})$. For any $\underline{g}\in\mathcal{R}^n$, let $\mathcal{P}\underline{g}$ be the vector in $\mathcal{R}^n$ that, for $i=1,2,\ldots,n$, has the $i$-th component $\min[0,g_i]$, $g_i$ or $\max[0,g_i]$ in the cases $(\underline{x}_{k+1})_i=a_i$, $a_i<(\underline{x}_{k+1})_i<b_i$ or $(\underline{x}_{k+1})_i=b_i$, respectively. We expect $\|\mathcal{P}\underline{\nabla}Q_{k+1}(\underline{x}_{k+1})\|$ to be much smaller than $\|\mathcal{P}\underline{\nabla}Q_{k+1}^{\text{alt}}(\underline{x}_{k+1})\|$ when the iterations are making good progress, but the ordering tends to be reversed if $\|\nabla^2 Q_{k+1}\|$ is huge. Therefore $Q_{k+1}$ is replaced by $Q_{k+1}^{\text{alt}}$ if the condition

$$\|\mathcal{P}\underline{\nabla}Q_{k+1}^{\text{alt}}(\underline{x}_{k+1})\|^2 \;\leq\; 0.1\,\|\mathcal{P}\underline{\nabla}Q_{k+1}(\underline{x}_{k+1})\|^2 \qquad (6.12)$$

holds on three consecutive iterations that take "trust region" steps, regardless of any intermediate "alternative" iterations. The factor 0.1 provides some reluctance to make the change, because it it is hardly ever worthwhile to interrupt the usual method of BOBYQA.

The importance of origin shifts to accuracy in practice can be deduced from the definition (4.11) of $\beta$ in the following setting. We assume that $\|\underline{x}_k-\underline{x}_0\|$ is much greater than the distances $\|\underline{x}^+-\underline{x}_k\|=\|\underline{d}_k\|$ and $\|\underline{y}_i-\underline{x}_k\|$, $i=1,2,\ldots,m$. It follows that the first $m$ components of expression (4.10) are about $w_i\approx\frac{1}{2}\,\|\underline{x}_k-\underline{x}_0\|^4$, $i=1,2,\ldots,m$. Moreover, we recall from the paragraph between equations (4.19) and (4.20) that in theory $\beta$ is independent of $\underline{x}_0$. Therefore the contribution from $\underline{x}_0$ to the first part of the formula $\beta=\frac{1}{2}\,\|\underline{x}^+-\underline{x}_0\|^4-\underline{w}^T H\,\underline{w}$ has to be annihilated by the other part of the formula, which includes the terms $w_i H_{ij} w_j\approx\frac{1}{4}\,\|\underline{x}_k-\underline{x}_0\|^8 H_{ij}$, $1\leq i,j\leq m$. The eighth power shows that huge damage from computer rounding errors would be likely in this hypothetical setting. BOBYQA restricts the amount of cancellation from $\underline{x}_0$ by considering the inequality

$$\|\underline{x}^+-\underline{x}_k\|^2 \;=\; \|\underline{d}_k\|^2 \;\leq\; 10^{-3}\,\|\underline{x}_k-\underline{x}_0\|^2 \qquad (6.13)$$

for every $\|\underline{d}_k\|$ that is at least $\frac{1}{2}\rho_k$. If condition (6.13) holds, then the position $\underline{x}_0$ of the origin is shifted immediately to $\underline{x}_k$.

The details of a shift are taken from NEWUOA (Powell, 2006). The $n\times m$ matrix $\Gamma$ that has the columns

$$\Gamma\underline{e}_j \;=\; \{\underline{s}^T(\underline{y}_j-\underline{x}_{\text{av}})\}\,(\underline{y}_j-\underline{x}_{\text{av}})+\tfrac{1}{4}\,\|\underline{s}\|^2\,\underline{s}, \qquad j=1,2,\ldots,m, \qquad (6.14)$$

is employed, where $\underline{s}$ and $\underline{x}_{\text{av}}$ are the shift $\underline{x}_k-\underline{x}_0$ and the mid-point $\frac{1}{2}(\underline{x}_0+\underline{x}_k)$, respectively. Corresponding to the second half of expression (2.7), we write the old $H$ without its $(m+1)$-th row and column in the partitioned form

$$H_{\text{red}} \;=\; \left(\begin{array}{c|c} \Omega & \Xi_{\text{red}}^T \\ \hline \Xi_{\text{red}} & \Upsilon_{\text{red}} \end{array}\right), \qquad (6.15)$$

$\Xi_{\rm red}$ being $\Xi$ without its first row and $\Upsilon_{\rm red}$ being $\Upsilon$ without its first row and column. The shift of origin requires $H_{\rm red}$ to be overwritten by the product

$$\left(\begin{array}{c|c} I & 0 \\ \hline \Gamma & I \end{array}\right) H_{\rm red} \left(\begin{array}{c|c} I & \Gamma^T \\ \hline 0 & I \end{array}\right). \tag{6.16}$$

In other words, $\Omega$ is unchanged as mentioned already, but the product $\Gamma\Omega$ and the sum of products $\Gamma\Xi_{\rm red}^T + \Xi_{\rm red}\Gamma^T + \Gamma\Omega\Gamma^T$ are added to $\Xi_{\rm red}$ and to $\Upsilon_{\rm red}$, respectively. Furthermore, the shift of origin implies a change to the representation (4.6) of $\nabla^2 Q_k$. As in the paragraph that includes equation (5.3), the old parameters $\mu_\ell$, $\ell = 1, 2, \ldots, m$, are retained, which requires the symmetric rank two matrix

$$\left\{ (\textstyle\sum_{\ell=1}^m \mu_\ell \underline{y}_\ell) - (\sum_{\ell=1}^m \mu_\ell)\, \underline{x}_{\rm av} \right\} \underline{s}^T + \underline{s} \left\{ (\textstyle\sum_{\ell=1}^m \mu_\ell \underline{y}_\ell) - (\sum_{\ell=1}^m \mu_\ell)\, \underline{x}_{\rm av} \right\}^T \tag{6.17}$$

to be added to the explicit part of $\nabla^2 Q_k$, namely $M$. The amount of work of these tasks is much greater than the routine work of a typical iteration, the number of computer operations being $\mathcal{O}(m^2 n)$ for every shift of origin. Therefore the frequency of shifts is one of the subjects of the numerical testing in the next section.

## 7. Numerical results

Some results when BOBYQA is applied to two test problems are presented and discussed in this section. The first problem is the minimization of the sum of squares

$$F(\underline{x}) = \sum_{i=1}^{2n} \left\{ f_i - \sum_{j=1}^{n} [\, S_{ij} \sin(x_j/\sigma_j) + C_{ij} \cos(x_j/\sigma_j) \,] \right\}^2, \qquad \underline{x} \in \mathcal{R}^n, \tag{7.1}$$

when the variables are unconstrained, the bounds of the constraints (1.1) being irrelevant because they are given the values $a_i = -10^{60}$ and $b_i = 10^{60}$, $i = 1, 2, \ldots, n$. The elements $S_{ij}$ and $C_{ij}$ are random integers from $[-100, 100]$, each $\sigma_j$ is chosen randomly from $[1, 10]$, and each $f_i$ is defined by $F(\underline{x}_*) = 0$, for a vector $\underline{x}_* \in \mathcal{R}^n$ that is also chosen randomly. Thus $F$ is periodic, with local maxima and saddle points and with a global minimum at $\underline{x} = \underline{x}_*$. The starting vector $\underline{x}_0$ is picked by letting the weighted differences $(\underline{x}_0 - \underline{x}_*)_j / \sigma_j$, $j = 1, 2, \ldots, n$, be random numbers from $[-\pi/10, \pi/10]$, and the values $\rho_{\rm beg} = 0.1$ and $\rho_{\rm end} = 10^{-6}$ are set. For each choice of $n$, five test problems are generated randomly. This description is taken from Powell (2008), and also we employ the same random numbers, but the switch from the NEWUOA to the BOBYQA software gives different numerical results, partly because of the major change to the construction of $\underline{d}_k$ on the "alternative" iterations.

The values of $\#F$ (total number of function evaluations), of $\|\underline{x}_f - \underline{x}_*\|_\infty$ where $\underline{x}_f$ is the final vector of variables, of #shifts (number of shifts of origin), and of

| $n$ | $m$ | Range of $\#F$ | $\|\underline{x}_f - \underline{x}_*\|_\infty$ | #shifts | #secs | #wkspace |
|---|---|---|---|---|---|---|
| 10 | 21 | 302–427 | $1.2\times10^{-6}$ | 7.8 | $3.6\times10^{-2}$ | 1,031 |
| 20 | 41 | 691–927 | $2.1\times10^{-6}$ | 12.8 | $3.3\times10^{-1}$ | 3,556 |
| 40 | 81 | 1681–2045 | $4.3\times10^{-6}$ | 19.0 | $2.9\times10^{0}$ | 13,106 |
| 80 | 161 | 3318–3609 | $5.5\times10^{-6}$ | 29.0 | $2.3\times10^{1}$ | 50,206 |
| 160 | 321 | 5570–6338 | $1.1\times10^{-5}$ | 51.4 | $1.9\times10^{2}$ | 196,406 |
| 320 | 641 | 11366–12047 | $1.9\times10^{-5}$ | 96.2 | $1.6\times10^{3}$ | 776,806 |

**Table 1:** BOBYQA applied to the test problem (7.1) with $m = 2n+1$

#secs (time measured by calling the Fortran procedure DTIME) are recorded for every test problem for every selection of $n$ and $m$. A summary of these results is presented in Tables 1–3, the three tables being for the three choices $m = 2n+1$, $m = n+6$ and $m = (n+1)(n+2)/2$, respectively. Each row of a table gives $n$, $m$, the least and greatest values of $\#F$, the greatest value of $\|\underline{x}_f - \underline{x}_*\|_\infty$, the average of #shifts and the average of #secs throughout the set of five test problems that is generated by different random numbers for the current $n$; the last figure in the row is the number of storage locations required by BOBYQA for working space. The third column of Table 1 is in very close agreement with the entries in Table 1 of Powell (2008). Indeed, when NEWUOA minimizes the objective function (7.1) with $m = 2n+1$ and with the same values of all the parameters, the ranges of $\#F$ over the five cases are 319–446, 780–999, 1629–2114, 3172–3497, 5589–6492 and 11391–12042 for $n = 10$, 20, 40, 80, 160 and 320, respectively.

The other information in Tables 1–3 is also typical of the NEWUOA software, the huge gain in efficiency for large $n$ when $m$ is reduced from $(n+1)(n+2)/2$ to $2n+1$ being known for many years. The greatest value of $n$ in Table 3 is only $n = 80$, because the amount of working space is $\mathcal{O}(n^4)$, and, if enough space were available, a calculation with $n = 160$ would take about 3 days. The improvement in the $\|\underline{x}_f - \underline{x}_*\|_\infty$ column of Table 3 over Tables 1 and 2 can be gained for the smaller values of $m$ by reducing the parameter $\rho_{\text{end}}$, which is going to be demonstrated in the other test problem of this section. All the entries in the #shifts column are tolerable, but the increase in the number of shifts when $m$ is reduced was unexpected. Several other experiments by the author have shown that the present advantages in Table 1 over Table 2, in particular the numbers of function evaluations, are usual but not general. Efficient choices of $m$ may be exposed by the question "how much second derivative information is needed in order to achieve a good rate of convergence". Successes with $m = n+6$ are remarkable, because then the conditions $Q_k(\underline{y}_j) = F(\underline{y}_j)$, $j = 1, 2, \ldots, m$, include only five independent data that are relevant to the second derivatives of the model.

The second test problem, which also receives attention in Powell (2008), seeks positions of a given number of points in the unit square $[0,1] \times [0,1] \subset \mathcal{R}^2$ that avoid as far as possible small distances between pairs of points. The number of

| $n$ | $m$ | Range of $\#F$ | $\Vert\underline{x}_f-\underline{x}_*\Vert_\infty$ | #shifts | #secs | #wkspace |
|---|---|---|---|---|---|---|
| 10 | 16 | 373–637 | $7.6\times10^{-6}$ | 15.2 | $3.6\times10^{-2}$ | 771 |
| 20 | 26 | 1499–1706 | $1.9\times10^{-5}$ | 32.6 | $3.8\times10^{-1}$ | 2,176 |
| 40 | 46 | 3490–4317 | $2.9\times10^{-5}$ | 63.2 | $3.3\times10^{0}$ | 7,086 |
| 80 | 86 | 8993–10079 | $3.9\times10^{-5}$ | 116.4 | $3.0\times10^{1}$ | 25,306 |
| 160 | 166 | 19074–21935 | $6.7\times10^{-5}$ | 213.0 | $2.9\times10^{2}$ | 95,346 |
| 320 | 326 | 43967–50144 | $1.4\times10^{-4}$ | 419.8 | $2.8\times10^{3}$ | 369,826 |

**Table 2:** BOBYQA applied to the test problem (7.1) with $m=n+6$

| $n$ | $m$ | Range of $\#F$ | $\Vert\underline{x}_f-\underline{x}_*\Vert_\infty$ | #shifts | #secs | #wkspace |
|---|---|---|---|---|---|---|
| 10 | 66 | 218–254 | $1.1\times10^{-7}$ | 3.8 | $9.6\times10^{-2}$ | 5,621 |
| 20 | 231 | 737–853 | $1.5\times10^{-7}$ | 5.0 | $3.8\times10^{0}$ | 59,986 |
| 40 | 861 | 2017–2222 | $8.5\times10^{-7}$ | 6.4 | $1.2\times10^{2}$ | 782,966 |
| 80 | 3321 | 7384–7578 | $1.9\times10^{-6}$ | 7.0 | $6.0\times10^{3}$ | 11,321,926 |

**Table 3:** BOBYQA applied to the test problem (7.1) with $m=(n+1)(n+2)/2$

variables $n$ is twice the number of points, the points being the vectors

$$\underline{p}_j = \begin{pmatrix} x_{2j-1} \\ x_{2j} \end{pmatrix}, \qquad j=1,2,\ldots,n/2. \tag{7.2}$$

The points are kept apart by trying to minimize the objective function

$$F(\underline{x}) = \textstyle\sum_{i=2}^{n/2} \sum_{j=1}^{i-1} \min\,[\,\Vert\underline{p}_i-\underline{p}_j\Vert^{-1},\, 10^3], \qquad \underline{x}\in\mathcal{R}^n, \tag{7.3}$$

each distance $\Vert\underline{p}_i-\underline{p}_j\Vert$ being Euclidean, and the points are confined to the unit square by the constraints

$$0 \le x_i \le 1, \qquad i=1,2,\ldots,n. \tag{7.4}$$

We call this problem "points in square".

It has many different local minima due to the following property. Let $\underline{p}_\ell$, say, be on an edge of the square. We consider a change to the current variables that moves $\underline{p}_\ell$ along the line perpendicular to the edge, the direction of the move being the one allowed by the bounds (7.4), but all the other points remain fixed. Let $\underline{p}_i$ be any one of these fixed points. At the beginning of the move, the first order change to $\Vert\underline{p}_i-\underline{p}_\ell\Vert^{-1}$ is always a strict increase if $\underline{p}_i$ is not on the edge under consideration, and in the alternative situation the initial first order change to $\Vert\underline{p}_i-\underline{p}_\ell\Vert^{-1}$ is zero. Therefore, unless the current points are all on the relevant edge of the square or are within distance $10^{-3}$ of $\underline{p}_\ell$, the initial directional derivative of

| $n$ | $m$ | Numbers of calculations of $F$ ($\#F$) Case 1 | Case 2 | Case 3 | Case $\widetilde{1}$ | Case $\widehat{1}$ | #shifts | #secs |
|---|---|---|---|---|---|---|---|---|
| 20 | 41 | 938 | 881 | 1115 | 953 | 871 | 15.2 | $2.8\times10^{-1}$ |
| 40 | 81 | 1672 | 6452 | 4519 | 1780 | 1744 | 39.8 | $4.1\times10^{0}$ |
| 80 | 161 | 7888 | 24220 | 45838 | 9012 | 6785 | 222.2 | $1.1\times10^{2}$ |
| 160 | 321 | 73776 | 71195 | 34018 | 46790 | 37210 | 543.0 | $1.5\times10^{3}$ |

**Table 4:** BOBYQA applied to "points in square" with $\rho_{\text{end}}=10^{-6}$ and $m=2n+1$

| $n$ | $m$ | Numbers of calculations of $F$ ($\#F$) Case 1 | Case 2 | Case 3 | Case $\widetilde{1}$ | Case $\widehat{1}$ | #shifts | #secs |
|---|---|---|---|---|---|---|---|---|
| 20 | 26 | 1667 | 1818 | 680 | 1683 | 745 | 31.4 | $2.2\times10^{-1}$ |
| 40 | 46 | 6870 | 2992 | 3706 | 2179 | 2011 | 69.2 | $1.9\times10^{0}$ |
| 80 | 86 | 14368 | 12859 | 13087 | 12346 | 8931 | 192.8 | $2.4\times10^{1}$ |
| 160 | 166 | 38581 | 44597 | 60387 | 34907 | 38545 | 514.4 | $3.4\times10^{2}$ |

**Table 5:** BOBYQA applied to "points in square" with $\rho_{\text{end}}=10^{-6}$ and $m=n+6$

$F(\underline{x})$, $\underline{x}\in\mathcal{R}^n$, for the change of variables is uphill. On the other hand, a positive steplength along this search direction may reduce the objective function, by taking $\underline{p}_\ell$ to a position that is relatively far from the other points. This construction with some other conditions gives a large source of local minima that are not global.

The number of variables $n$ is given the values 20, 40, 80 and 160 in the "points in square" testing. Five different problems are generated for each $n$ by different choices of the initial vector of variables $\underline{x}_0$. A random number generator is applied in three of these cases, namely Case 1, Case 2 and Case 3, each component of $\underline{x}_0$ being sampled independently from the uniform distribution on $[0,1]$, except that this construction is restarted if necessary so that the initial points (7.2) satisfy the condition

$$\min\{\,\|\underline{p}_i-\underline{p}_j\|:1\le j<i\le n/2\,\}\;\ge\;0.2\sqrt{2/n}, \qquad (7.5)$$

which provides a helpful restriction on $F(\underline{x}_0)$. The other cases are called Case $\widetilde{1}$ and Case $\widehat{1}$, because their initial vectors are chosen to be $(1-10^{-6})\underline{x}_0^{(1)}$ and $(1-10^{-6})\underline{x}_0^{(1)}+10^{-6}\underline{e}$, respectively, $\underline{x}_0^{(1)}$ being the initial vector of Case 1 and $\underline{e}$ being the vector in $\mathcal{R}^n$ whose components are all 1. Thus we investigate the sensitivity of the calculations to small perturbations of the data. We compare only $m=n+6$ with $m=2n+1$, because, as in Table 3, the choice $m=(n+1)(n+2)/2$ is unsuitable for large $n$. We pick $\rho_{\text{beg}}=0.1$ and $\rho_{\text{end}}=10^{-4}$, $10^{-6}$ or $10^{-8}$.

The values of $\#F$ for all of these "points in square" test problems are presented in Tables 4 and 5 for $\rho_{\text{end}}=10^{-6}$. We see that, for every $\{n,m\}$ pair, there are

large variations in $\#F$ across the five cases, even if one compares only Cases 1, $\widetilde{1}$ and $\widehat{1}$. Moreover, the final values $F(\underline{x}_f)$ of the objective function in the ten experiments with $n=160$, for instance, are the different numbers (in ascending order) 6850.0, 6853.5, 6855.2, 6857.9, 6861.3, 6863.9, 6864.0, 6870.0, 6870.1 and 6876.1. Thus the question arises whether ten different local minima have been found or whether some of the differences are due to the limited precision of the computer arithmetic. This question is answered later by the experiments with $\rho_{\text{end}}=10^{-8}$. A comparison of the last two columns of Tables 4 and 5 with the corresponding columns of Tables 1–3 shows that the numbers of shifts of origin remain tolerable, but now the decrease from $m=2n+1$ to $m=n+6$ provides a substantial reduction in the running times of the experiments.

The accuracy of the "points in square" calculations is estimated by considering the first order conditions for a local minimum. At every final vector of variables $\underline{x}_f$, all the terms $\|\underline{p}_i-\underline{p}_j\|^{-1}$ of expression (7.3) are less than $10^3$, and then $F(\underline{x})$, $\underline{x}\in\mathcal{R}^n$, has the first derivatives

$$\left.\begin{array}{lcl} dF/dx_{2i-1} & = & \sum_{j=1,\,j\neq i}^{n/2} (x_{2j-1}-x_{2i-1})\,\|\underline{p}_i-\underline{p}_j\|^{-3} \\ dF/dx_{2i} & = & \sum_{j=1,\,j\neq i}^{n/2} (x_{2j}-x_{2i})\,\|\underline{p}_i-\underline{p}_j\|^{-3} \end{array}\right\}, \quad i=1,2,\ldots,n/2. \qquad (7.6)$$

We prefer to study relative errors in first order conditions, so we let $\widehat{\underline{g}}(\underline{x})\in\mathcal{R}^n$ have the components

$$\left.\begin{array}{lcl} \widehat{g}_{2i-1} & = & \sum_{j=1,\,j\neq i}^{n/2} U_{ij} \Big/ \sum_{j=1,\,j\neq i}^{n/2} |U_{ij}| \\ \widehat{g}_{2i} & = & \sum_{j=1,\,j\neq i}^{n/2} V_{ij} \Big/ \sum_{j=1,\,j\neq i}^{n/2} |V_{ij}| \end{array}\right\}, \qquad i=1,2,\ldots,n/2, \qquad (7.7)$$

where $U_{ij}$ and $V_{ij}$ are the terms $(x_{2j-1}-x_{2i-1})\|\underline{p}_i-\underline{p}_j\|^{-3}$ and $(x_{2j}-x_{2i})\|\underline{p}_i-\underline{p}_j\|^{-3}$ of expression (7.6), respectively. Furthermore, we take account of the bounds (7.4), which must be satisfied, by setting the components of $\check{\underline{g}}(\underline{x})\in\mathcal{R}^n$ to the values

$$\check{g}_\ell = \left\{\begin{array}{ccc} \min\,[0,\widehat{g}_\ell] & \text{if} & x_\ell=0 \\ \widehat{g}_\ell & \text{if} & 0<x_\ell<1 \\ \max\,[0,\widehat{g}_\ell] & \text{if} & x_\ell=1 \end{array}\right\}, \qquad \ell=1,2,\ldots,n. \qquad (7.8)$$

It is elementary that the first order conditions for a local minimum of "points in square" are achieved at $\underline{x}$ if and only $\check{\underline{g}}(\underline{x})$ is zero.

The $\rho_{\text{end}}=10^{-6}$ columns of Tables 6 and 7 present the averages of the five values of $\#F$ in the rows of Tables 4 and 5 and also the greatest values of $\|\check{\underline{g}}(\underline{x}_f)\|_\infty$ from the five cases of each row. The other columns provide the corresponding results for the choices $\rho_{\text{end}}=10^{-4}$ and $\rho_{\text{end}}=10^{-8}$, keeping $\rho_{\text{beg}}=0.1$ as mentioned already. Of course the smaller values of $\rho_{\text{end}}$ cause increases in $\#F$, while the $\|\check{\underline{g}}(\underline{x}_f)\|_\infty$ figures show clearly that reductions in $\rho_{\text{end}}$ yield better accuracy. The "Average $\#F$" entries in Tables 6 and 7 are a triumph for $m=n+6$, which may be due to the possibility that second derivative information is less helpful when there are many local minima.

| $n$ | $m$ | Average $\#F$ / Greatest $\|\underline{\breve{g}}(\underline{x}_f)\|_\infty$ | | |
|---|---|---|---|---|
| | | $\rho_{\rm end}=10^{-4}$ | $\rho_{\rm end}=10^{-6}$ | $\rho_{\rm end}=10^{-8}$ |
| 20 | 41 | 835.0 / $4.3\times10^{-4}$ | 951.6 / $2.0\times10^{-6}$ | 1052.2 / $6.1\times10^{-8}$ |
| 40 | 81 | 2242.8 / $1.2\times10^{-3}$ | 3233.4 / $1.3\times10^{-5}$ | 3718.6 / $4.9\times10^{-7}$ |
| 80 | 161 | 7096.2 / $3.8\times10^{-3}$ | 18748.6 / $3.0\times10^{-5}$ | 20864.6 / $1.5\times10^{-6}$ |
| 160 | 321 | 23431.2 / $5.6\times10^{-3}$ | 52597.8 / $3.3\times10^{-5}$ | 67024.4 / $2.7\times10^{-6}$ |

**Table 6:** A comparison of 3 values of $\rho_{\rm end}$ for "points in square" with $m=2n+1$

| $n$ | $m$ | Average $\#F$ / Greatest $\|\underline{\breve{g}}(\underline{x}_f)\|_\infty$ | | |
|---|---|---|---|---|
| | | $\rho_{\rm end}=10^{-4}$ | $\rho_{\rm end}=10^{-6}$ | $\rho_{\rm end}=10^{-8}$ |
| 20 | 26 | 1154.8 / $2.9\times10^{-3}$ | 1318.6 / $1.9\times10^{-5}$ | 1471.8 / $2.1\times10^{-7}$ |
| 40 | 46 | 2317.0 / $2.2\times10^{-3}$ | 3551.6 / $4.2\times10^{-5}$ | 4294.6 / $8.5\times10^{-7}$ |
| 80 | 86 | 6311.8 / $4.8\times10^{-3}$ | 12318.2 / $6.4\times10^{-5}$ | 15353.6 / $1.8\times10^{-6}$ |
| 160 | 166 | 15138.6 / $3.1\times10^{-3}$ | 43403.4 / $5.6\times10^{-5}$ | 52791.0 / $3.9\times10^{-6}$ |

**Table 7:** A comparison of 3 values of $\rho_{\rm end}$ for "points in square" with $m=n+6$

It has been noted that the final values of the objective function in the ten cases with $n=160$ and $\rho_{\rm end}=10^{-6}$ include 6863.9, 6864.0, 6870.0 and 6870.1, and now we address the question whether the differences of about 0.1 are due to the finite precision of the computer arithmetic or to the plethora of local minima. The $\|\underline{\breve{g}}(\underline{x}_f)\|_\infty$ entries in Tables 6 and 7 suggest that every calculated $\underline{x}_f$ is close to a local minimum, at $\underline{x}_* \in \mathcal{R}^n$ say. Further, because of the substantial decreases in $\|\underline{\breve{g}}(\underline{x}_f)\|_\infty$ when $\rho_{\rm end}$ is reduced from $10^{-6}$ to $10^{-8}$, we expect the $\rho_{\rm end}=10^{-6}$ values of $F(\underline{x}_f)-F(\underline{x}_*)$ to agree closely with the differences between the $\rho_{\rm end}=10^{-6}$ and $\rho_{\rm end}=10^{-8}$ values of $F(\underline{x}_f)$. The greatest of these differences throughout the $n=160$ calculations of Tables 6 and 7 is only 0.000004. Thus we conclude that the values of $F(\underline{x}_f)$, given above to one decimal place, belong to four different local minima. These remarks illustrate not only the difficulty of the "points in square" test problem but also the success of BOBYQA in finding local minima to high accuracy.

There were no calls of the RESCUE procedure of Section 5 throughout the numerical experiments that produced Tables 1–7. Therefore losses of precision that cause error returns from BOBYQA are unusual. On the other hand, computer rounding errors may be contributing strongly to the wideness of the range of $\#F$ in the rows of Tables 4 and 5, this suggestion being made because of a similar situation in the early development of the NEWUOA software for unconstrained optimization. Indeed, when the $\Omega$ submatrix of expression (2.7) was stored and updated explicitly, instead of employing the factorization $\Omega=ZZ^T$ where $Z$ has

only $m-n-1$ columns, the ranges of $\#F$ for the test problem (7.1) were as chaotic as the ranges in Tables 4 and 5. The factorization provided the stability that is shown in the "Range of $\#F$" columns of Tables 1 and 2.

It was not easy to decide to release the Fortran software for general use, instead of seeking further improvements. It was hoped that BOBYQA would become more efficient than NEWUOA for unconstrained calculations, but there is no clear winner. Two techniques that may reduce $\#F$ in the future are automatic adjustments of $m$ (the number of interpolation points), and taking up the freedom in $Q_{k+1}$ by minimizing a combination of $\|\nabla^2 Q_{k+1} - \nabla^2 Q_k\|_F$ with a term that includes some changes to first derivatives of the current quadratic model. Research on these questions is not needed urgently, because the present version of BOBYQA can provide local minima of a wide range of functions of hundreds of variables subject to simple bound constraints. The Fortran listing of BOBYQA is available free of charge from the author at the e-mail address mjdp@cam.ac.uk.

**Acknowledgement**

Parts of the development of BOBYQA were made during two visits by the author to the Liu Bie Ju Centre for Mathematical Sciences at the City University of Hong Kong. The excellent encouragement and support that I received there were very welcome and helpful.

**References**

A.R. Conn, K. Scheinberg and L.N. Vicente (2009), *Introduction to Derivative-Free Optimization*, SIAM Publications (Philadelphia).

N.I.M. Gould and Ph.L. Toint (2004), "How mature is nonlinear optimization?", in *Applied Mathematics Entering the 21st Century: Invited Talks from the ICIAM 2003 Congress*, editors J.M. Hill and R. Moore, SIAM Publications (Philadelphia), pp. 141–161.

M.J.D. Powell (2004), "Least Frobenius norm updating of quadratic models that satisfy interpolation conditions", *Math. Programming B*, Vol. 100, pp. 183–215.

M.J.D. Powell (2006), "The NEWUOA software for unconstrained optimization without derivatives", in *Large-Scale Optimization*, editors G. Di Pillo and M. Roma, Springer (New York), pp. 255–297.

M.J.D. Powell (2008), "Developments of NEWUOA for minimization without derivatives", *IMA J. Numer. Anal.*, Vol. 28, pp. 649–664.
